# End game strategies towards the total synthesis of vibsanin E, 3-hydroxyvibsanin E, furanovibsanin A, and 3-*O*-methylfuranovibsanin A

**DOI:** 10.3762/bjoc.4.34

**Published:** 2008-10-08

**Authors:** Brett D Schwartz, Craig M Williams, Paul V Bernhardt

**Affiliations:** 1 School of Molecular and Microbial Sciences, University of Queensland, Brisbane, 4072, Queensland, Australia

**Keywords:** diterpenes, furanovibsanin A, 3-hydroxyvibsanin E, 3-*O*-methylfuranovibsanin A, natural products, terpenoids, vibsane, vibsanin E, Viburnum

## Abstract

End game synthetic strategy studies towards the total synthesis of the vibsanin type diterpenes, vibsanin E, 3-hydroxyvibsanin E, furanovibsanin A, and 3-*O*-methylfuranovibsanin A are discussed, with focus on construction of the side chain and peripheral functionality associated with this group of natural products is the current focus of this report.

## Introduction

**Figure 1:**
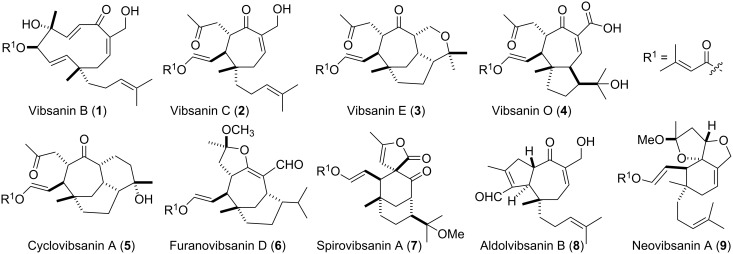
A collection of the structural diversity seen in the vibsanin type diterpene family.

Vibsane-type diterpenes occur exclusively in *Viburnum* species such as *V. awabuki* [1], *V. odoratissimum* [2] and *V. suspensum* [3], and can be regarded as quite rare natural products. Nine structure subtypes have so far been isolated from this family, for example, vibsanin B (**1**) [1], vibsanin C (**2**) [1], vibsanin E (**3**) [1], vibsanin O (**4**) [4], cyclovibsanin A (**5**) [5], furanovibsanin D (**6**) [6], spirovibsanin A (**7**) [7], aldolvibsanin B (**8**) [8], and neovibsanin A (**9**) [9] (Figure 1).

**Figure 2:**
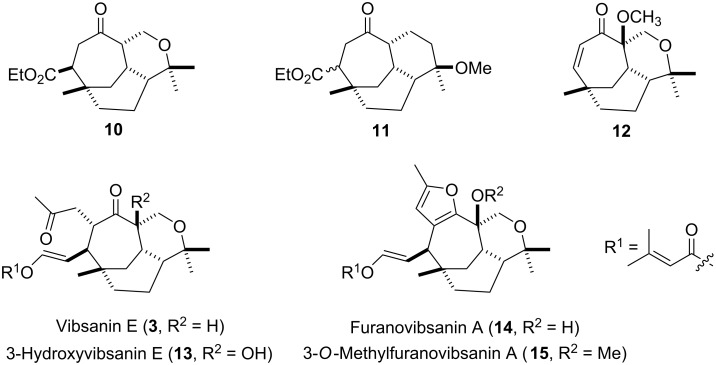
Vibsanin type diterpene synthetic targets.

In previous reports our group detailed biogenetically modelled approaches to rapidly access the central core of family members of type **3** [10–11], **5** [10,12] and **7** [13–15] (Figure 1). We now detail end game synthetic strategy studies towards the total synthesis of the vibsanin type diterpenes, vibsanin E (**3**), 3-hydroxyvibsanin E (**13**), furanovibsanin A (**14**), and 3-*O*-methylfuranovibsanin A (**15**) (Figure 2) building on core structures **10**–**12** (Figure 2).

## Results and Discussion

**Scheme 1:**
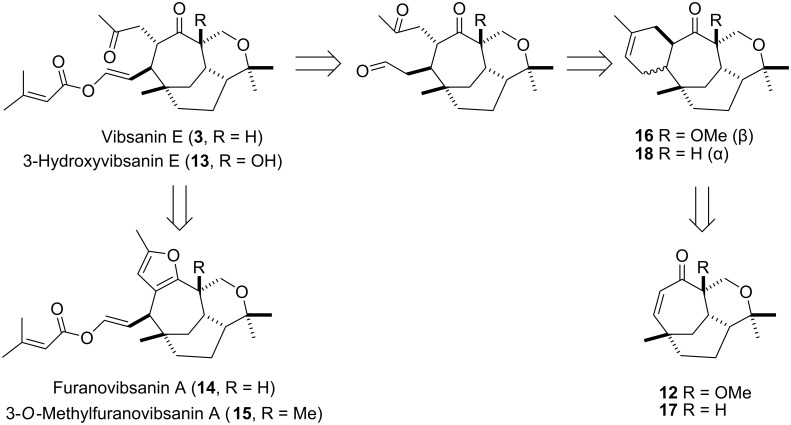
Retrosynthesis of vibsanin type targets.

As shown in the first generation retrosynthesis (Scheme 1) a [4+2] cycloaddition to install the required functionality was envisaged. All attempts, however, to procure this transformation (i.e. **16**), that is reaction of isoprene and oxygenated derivatives, with enone **12** completely failed. Davies [16–17], however, demonstrated that a photochemical assisted thermal [4+2] cycloaddition does proceed but with incorrect relative stereochemistry and limited regiocontrol (i.e. **18**). Nevertheless, Davies [16] pursued and completed an elegant synthesis of (±)-5,10-bis-*epi*-vibsanin E based on their cycloaddition methodology.

**Scheme 2:**
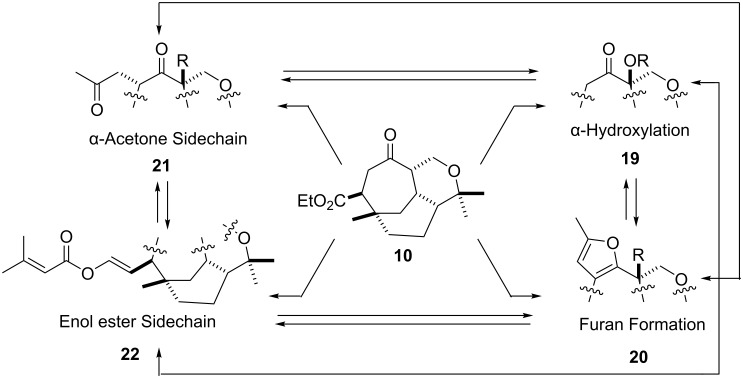
The four functional group areas identified for investigation.

With this knowledge in hand, and the availability of racemic **10** [10–11], attention was directed towards stepwise introduction of the required sidechain and corresponding α-oxo functionality depicted in Scheme 2. Essentially four areas were identified for study; 1) regio- and stereospecific α-hydroxylation (methoxylation) **19**, 2) furan formation i.e. **20**, 3) installing the acetone sidechain i.e. **21**, and 4) building the enol ester function i.e. **22** (Scheme 2). The results of each area of investigation allow end game strategies to be postulated based on combinations of these results. For example, success with α-hydroxylation (methoxylation) **19** could flow into furan formation (i.e. **20**), installing the acetone sidechain i.e. **21**, or building the enol ester function i.e. **22**, with subsequent flow into each area to attempt total synthesis (Scheme 2).

**Scheme 3:**
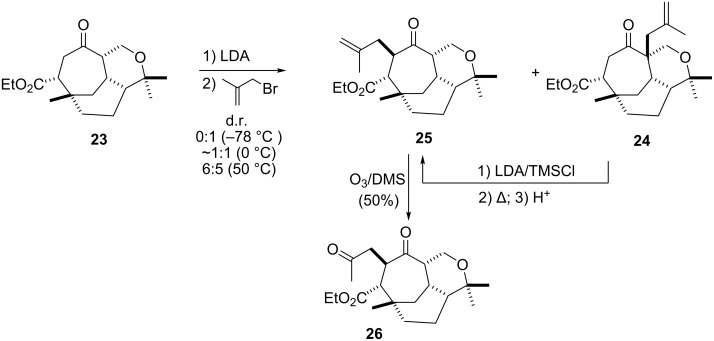
Acetone sidechain studies.

**Figure 3:**
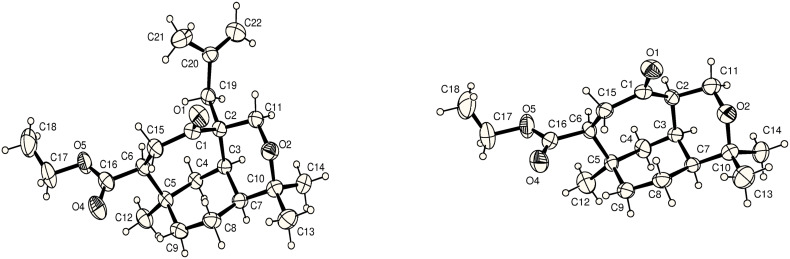
ORTEP diagrams of compounds **24** and **23** (30% probability elipsoids).

The first area of study [18] concentrated on implementation of the acetone sidechain. Enolate chemistry was the only viable option in this regard and as such two electrophiles were investigated. Initially the lithium enolate of **23** (best generated with LDA) was reacted with bromoacetone but this afforded only trace amounts of product. Switching to the more active electrophile methallyl bromide gave the desired methallylated product **25** in an optimized yield of 37% along with the undesired regioisomer **24**. Temperature was critical to the outcome of the reaction. At −78 °C only undesired regioisomer **24** was obtained in low yield (11%). However, when the enolate was quenched at 0 °C the desired regioisomer **25** was obtained in 15% yield along with the undesired isomer **24** in 17% (Scheme 3, Figure 3). The ratio and yield could be further improved [**25** (37%) : **24** (25%)] if the enolate solution was heated to 50 °C before addition of the electrophile. The difficulty in overcoming a significant preference for the undesired regioisomer **24** could be attributed to a number of combined, or individual, factors. For example, the first formed enolate could be stabilised by overlap of the π orbital with the σ^*^C–O orbital [19], or because the tertiary bridgehead hydrogen is a longer C-H bond than the secondary hydrogen C-H bond, which is kinetically favoured. Conversion of the undesired isomer **24** into the desired (i.e. **25**) by a Claisen rearrangment (via the silyl enol ether) was not high yielding and produced many side products.

Ozonolysis of **25** afforded the acetone sidechain (i.e. **26**) in acceptable yield (50%). Other methods to unmask the ketone functionality failed, for example, dihydroxylation followed by oxidative cleavage. Nevertheless, the acetone sidechain could be introduced in ~20% overall yield allowing end game functionalisation (as discussed below).

**Scheme 4:**
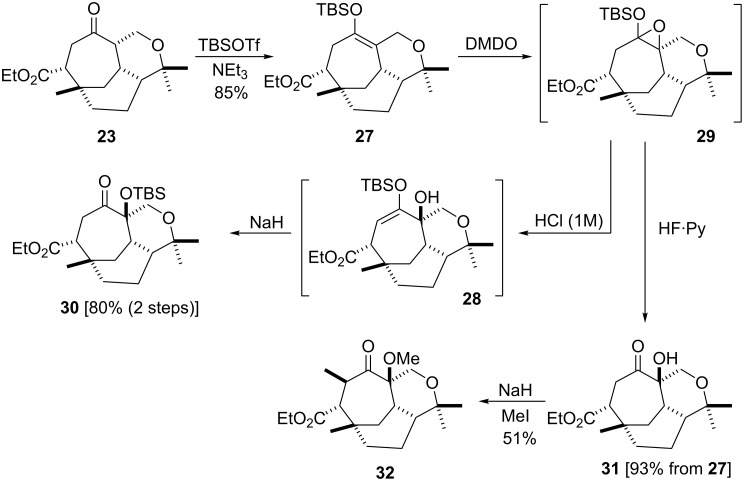
α-Hydroxylation investigations.

**Figure 4:**
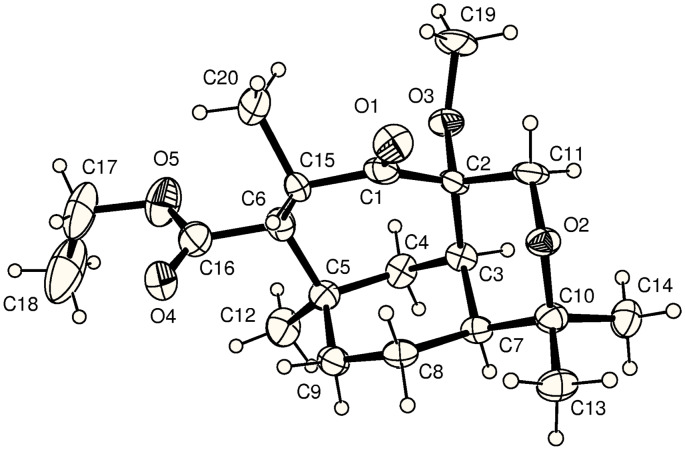
ORTEP diagram of compound **32** (30% probability ellipsoids).

α-Hydroxylation was next investigated. Considering the observed preference for regiospecific enolate formation in our system we devised a simple two pot procedure based on the epoxidation of silyl enol ethers. Ketone **23** was smoothly converted into the TBS enol ether **27** (85% yield) with TBS triflate, which was then treated with dimethyldioxirane (DMDO). When work up was restricted to a simple 1 M hydrochloric acid wash (i.e. separatory funnel) only the epoxide ring opened product (i.e. **28**) was isolated (via epoxide **29**). Subsequent treatment of the crude material (i.e. **28**) with sodium hydride gave as the sole product the TBS protected α-hydroxy ketone **30** in 80% yield over two steps, via a 1,2-Brook rearrangement. The unprotected derivative **31** could be obtained in 93% yield from **27**, via **29**, if hydrogen fluoride was used. Unfortunately, methylation of the hydroxy group in compound **31** was unsuccessful since unavoidable C-methylation also occurred to afford **32** (Scheme 4, Figure 4).

**Scheme 5:**
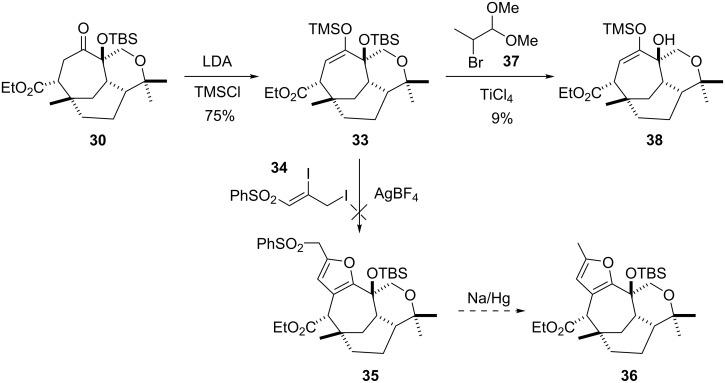
Investigating literature methods to install the furan ring system.

In the view that α-hydroxylation, in the form of TBS protection, proceeded so efficiently furan ring formation was investigated with ketone **30**. Three general protocols were identified as suitable for attempting fused furan formation with substrate **30**; 1) Padwa [20] and Mukaiyama [21] furan synthesis, 2) Nishizawa furan synthesis [22], and 3) classical acid catalysed diketone dehydration (i.e. phosphorus pentaoxide [23]). For Padwa’s protocol the TMS enol ether **33** was required, which was obtained in 75% from sequential treatment of **30** with LDA and TMSCl. Subsequent reaction of **33** with Padwa’s electrophile **34** [24] and silver tetrafluoroborate gave a complex mixture with no identifiable trace of desired product **35**, a precusor to desired furan **36** (Scheme 5). The lack of reactivity was without doubt substrate specific (i.e. **33**), as model studies on the TMS enol ether of cycloheptanone gave the expected furan product using Padwa’s protocol. Mukaiyama reported [21] the use of electrophile **37** to access the furan ring system using similar conditions to that of Padwa, however, this returned mostly starting material and traces of the tertiary hydroxy compound **38** (Scheme 5).

**Scheme 6:**
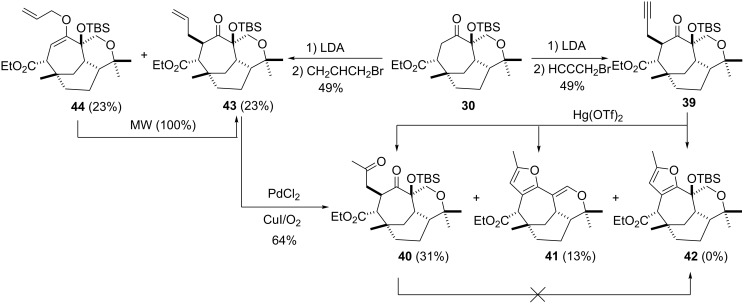
Installation of the furan ring system continued.

Nishizawa reported [22] the conversion of α-propargyl substituted ketones directly into methylated furans using catalytic amounts of mercury triflate. Although verification of this protocol was undertaken on a cycloheptanone derivative, substrate **39** failed to give the desired furan **42** (Scheme 6). Instead, hydration was observed as the major reaction pathway (i.e. **40**) with furan **41** being obtained as the minor component. Furan **41** is an interesting molecule in that it contains a bridgehead double bond, presumably formed due to the ease of carbocation formation at the benzylic (tertiary) centre. Unfortunately, the bridgehead double bond contained within **41** could not be hydrated. Conversion of diketone **40**, which could be accessed from **43** (and **44**) via a Wacker oxidation (64%), also failed to yield furan functionality using classical conditions (i.e. phosphorus pentaoxide and amberlyst resin) (Scheme 6).

**Scheme 7:**
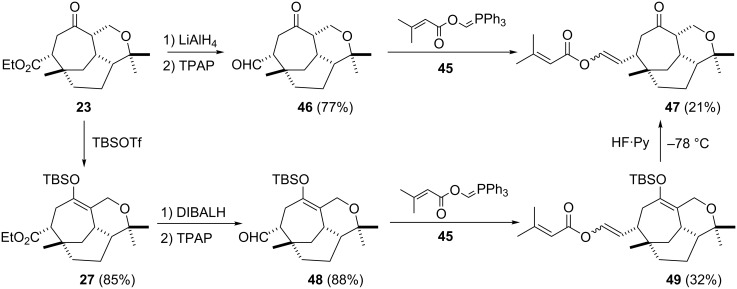
Installation of the enol sidechain utilizing Wittig chemistry.

Enol ester sidechain construction: although Davies [16] has reported the construction of the enol ester sidechain (3,3-dimethylacrylic anhydride, 4-pyrrolidinopyridine) associated with the vibsanin family members this functionality was derived from a two carbon chain aldehyde (i.e. CH_2_CHO). In the current case (i.e. **23**) the ester function would require homologation or new methodology to install the enol ester sidechain from one carbon unit (i.e. aldehyde). Considering one carbon homologation would demand multiple steps we opted to develop new methodology. A literature search revealed the work of Anders [25–30], which utilized methyleneoxy ylids of type **45**. Our modification [31] introduced 3-methylcrotonate functionality (i.e. **45**), which gave similar yields to that reported for the benzoate and related studies [25–30]. For example, treating **23** with lithium aluminium hydride followed by Swern oxidation gave **46** (77% over two steps) which when treated with **45** gave the desired material **47** in 21% yield with an *E*/*Z* ratio of 3.4:1 respectively (Scheme 7). This could be improved if the reduction/oxidation [32] sequence was performed on the TBS enol ether **27**, which gave **48** in 88% yield and subsequently gave **49** in 32% yield *E* (2.4) : *Z* (1)]. Enol ether **49** could be conveniently converted in 92% yield to **47** by treating **49** with hydrogen fluoride pyridine complex at −78 °C (Scheme 7).

With the four areas of study complete [i.e. α-hydroxylation, furan formation, acetone sidechain, and enol ester function (Scheme 2)] formulation of suitable end game stategies could now be undertaken. In summary, these studies showed that α-hydroxylation was viable and high yielding, the incorporation of the acetone and enol ether sidechains were possible but moderately yielding, and furan formation was not viable. On this basis only two targets seemed approachable: 1) bis-*epi*-vibsanin E **50**, and 2) bis-*epi*-3-hydroxyvibsanin E **51**.

**Scheme 8:**
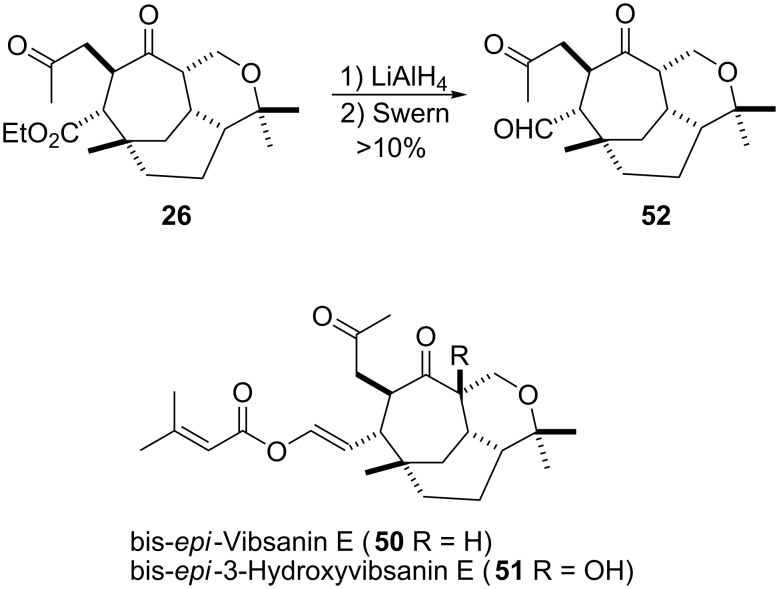
Attempts to gain access to targets **50** and **51**.

Initial studies concentrated on **26**, in that tricarbonyl reduction followed by oxidation was envisaged to give aldehyde **52**, which could then undergo reaction with ylid **45** in the hope of gaining access to bis-*epi*-vibsanin E **50**. Reduction with lithium aluminium hydride proceded smoothly, however, global oxidation caused significant problems yielding only very low amounts of aldehyde **52**, which was not enough to attempt the Wittig reaction with **45** (Scheme 8).

In the view of the diasppointing results obtained above (Scheme 8) all attention was directed towards bis-*epi*-3-hydroxyvibsanin E **51**. This manoeuvre was further justified by the fact that diketone **40** was readily available via the allylation/Wacker protocol as described in Scheme 6.

**Scheme 9:**
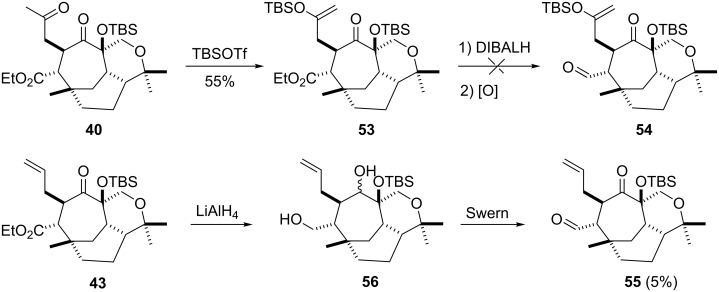
Further attempts to gain access to target compound **51**.

Considering the knowledge gained in Scheme 8, it was perceived best not to perform tricarbonyl reduction then oxidation on diketone **40**, but to first protect the ketone functionality as silyl enol ethers as was undertaken in Scheme 7 (i.e. **27**–**48**). Treating diketone **40** with *t*-butyldimethylsilyl trifluoromethanesulfonate afforded only the monoprotected product **53** (crude yield 55%), which smoothly underwent reduction with diisobutylaluminium hydride, but all attempts to oxidise the diol to **54** failed (Scheme 9). Oxidation and reduction problems occurred also when working with ketone **43**, for example, ketone **43** gave only partial reduction and subsequent oxidation of diol **56** gave the aldehyde **55** only in 5% yield (Scheme 9).

## Conclusion

In conclusion, we have investigated the construction of four different functionality types [i.e. α-hydroxylation, furan formation, acetone and enol ester sidechain functions (Scheme 2)] associated with the vibsanin family of natural products. These studies were vital for investigating end game strategies for attempting total syntheses of vibsanin E, 3-hydroxyvibsanin E, furanovibsanin A, and 3-*O*-methylfuranovibsanin A. Unfortunately, the optimum combination of functional group installation could not be found. Nevertheless, valuable insights into the scope and limitations of some literature methods called upon for the attempted total synthesis of this family of natural products were gained.

## Supporting Information

File 1 Experimental

File 2 NMR spectra
