# Supplementary material for: End game strategies towards the total synthesis of vibsanin E, 3-hydroxyvibsanin E, furanovibsanin A, and 3-O-methylfuranovibsanin A
Source: Beilstein J Org Chem. 2008 Oct 8;4:34. doi: 10.3762/bjoc.4.34 (PMC2577281; doi:10.3762/bjoc.4.34)
Supplement: File 1 — Experimental [file Beilstein_J_Org_Chem-04-34-s001.doc]

**End game strategies towards the total synthesis of vibsanin E,
3-hydroxyvibsanin E, furanovibsanin A,
and 3-*O*-methylfuranovibsanin A**

Brett D. Schwartz, Craig M. Williams^*^ and Paul V. Bernhardt

Address: School of Molecular and Microbial Sciences, University of Queensland, Brisbane, 4072, Queensland, Australia

Email: Craig M. Williams^*^ - c.williams3@uq.edu.au

* Corresponding author

**Experimental**

^1^H and ^13^C NMR spectra were recorded on Bruker AV400 (400.13 MHz; 100.62 MHz), AV300 (300.13 MHz; 75.47 MHz) and DRX500 (500.13 MHz; 125.76 MHz) instruments in the solvents specified. Coupling constants are given in Hz and chemical shifts are expressed as δ values in ppm. The GC/MS data were recorded on a Shimadzu QP5050 gas chromatograph – mass spectrometer at 70 eV, fitted with a 30 m × 0.25 mm BP5 column. The standard program was set as 2 minutes at 100 °C, followed by a temperature increase of 16 °C/min, and left for 10 min at 250 °C. High resolution electrospray ionisation (HRESIMS) accurate mass measurements were recorded in positive mode on a Bruker MicrOTOF-Q (quadrupole – Time of Flight) instrument with a Bruker ESI source. Accurate mass measurements were carried out with external calibration using sodium formate as reference calibrant. Microanalyses were performed by the University of Queensland Microanalytical Service. Column chromatography was undertaken on silica gel (Flash Silica gel 230–400 mesh), with distilled solvents. Anhydrous solvents were prepared according to Perrin and Armarego [1]. Tetrahydrofuran was freshly distilled from a sodium/benzophenone still. Melting points were determined on a Fischer Johns Melting Point apparatus and are uncorrected. Fine chemicals were purchased from the Aldrich Chem. Co. Microwave irradiation was conducted with a CEM Discover microwave in 10 mL or 80 mL pressurized vials.

**Methallylation of ketone 23 (preparation of 24 and 25):** To a cold (0 °C) stirring solution of anhydrous diisopropylamine (100 μL, 0.722 mmol) in anhydrous tetrahydrofuran (5 mL) under an argon atmosphere, was added *n*-butyllithium (1.55 M in hexanes, 444 μL, 0.689 mmol) dropwise. The above LDA solution was then added via syringe to an anhydrous tetrahydrofuran (8.5 mL) solution of ketone **23** (202 mg, 0.656 mmol) under an argon atmosphere at 0 °C. The reaction mixture was then heated to 50 °C and held for 2 min. Subsequently HMPA (200 μL) was added followed by methallyl bromide (97 mg, 72 μL, 0.722 mmol). After 5 min the reaction was quenched by pouring into ice-cold ammonium chloride (saturated aqueous solution). Extraction with ether (3 × 15 mL), followed by washing with brine (10 mL), drying (Na_2_SO_4_) and evaporation resulted in an oily residue. Column chromatography (10:1 petroleum spirit/diethyl ether) afforded ester **24** as a colourless oil (60 mg, 25%) and ester **25** as a colourless oil (89 mg, 37%). Compound **24** ^1^H NMR (400 MHz, CDCl_3_) δ 0.96 (s, 3H), 1.06 (s, 3H), 1.10−1.22 (m, 1H), 1.25 (t, *J* = 7.1 Hz, 3H), 1.26 (s, 3H), 1.49 (dd, *J* = 14.2, 4.4 Hz, 1H), 1.52−1.64 1.49 (m, 3H), (dd, *J* = 1.3, 0.7 Hz, 3H), 2.09 (d, *J* = 14.4 Hz, 1H), 2.22−2.30 (m, 2H), 2.36−2.45 (m, 1H), 2.49 (d, *J* = 13.7 Hz, 1H), 2.69−2.80 (m, 2H), 3.16 (dd, *J* = 12.1, 18.5 Hz, 1H) 3.21 (d, *J* = 11.5 Hz, 1H), 4.06−4.18 (m, 2H), 4.29 (d, *J* = 11.7 Hz, 1H), 4.62−4.64 (m, 1H), 4.82−4.84 (m, 1H). ^13^C NMR (100 MHz, CDCl_3_) δ 14.2, 22.3, 24.0, 24.5, 27.3, 31.8, 32.2, 33.4, 38.6, 41.8, 43.8, 43.9, 44.2, 51.3, 54.7, 60.5, 66.7, 73.4, 116.0, 140.2, 174.5, 210.0. GC/MS EI *m/z* (%) 362 (M^+^˙, 2), 347 (3), 334 (4), 307 (56), 306 (100), 287 (10), 233 (17), 164 (48), 135 (52), 94 (88), 81 (46), 79 (58), 67 (43), 55 (66), 41 (70). Compound **25** ^1^H NMR (400 MHz, CDCl_3_) δ 0.94 (s, 3H), 1.06 (td, *J* = 5.6, 4.8 Hz, 1H), 1.18 (s, 3H), 1.27 (s, 3H), 1.27 (t, *J* = 7.1 Hz, 3H), 1.35−1.42 (m, 1H), 1.56−1.72 (m, 4H), 1.68 (s, 3H), 1.88−1.96 (m, 2H), 2.09−2.13 (ddd, *J* = 14.3, 6.4, 3.1 Hz, 1H), 2.23 (d, *J* = 12.4 Hz, 1H), 2.45 (dd, *J* = 13.4, 9.9 Hz, 1H), 2.51−2.59 (m, 2H), 3.75−3.83 (m, 3H), 4.15 (q, *J* = 4.16 Hz, 2H), 4.62 (s, 1H), 4.71 (s, 1H). ^13^C NMR (100 MHz, CDCl_3_) δ 14.2, 22.3, 24.0, 24.5, 27.3, 31.8, 32.3, 33.4, 38.6, 41.8, 43.8, 43.9, 44.2, 51.3, 54.7, 60.5, 66.7, 73.4, 116.0, 140.2, 174.5, 210.0. GC/MS EI *m/z* (%) 362 (M^+^˙, 18), 347 (12), 333 (10), 319 (7), 289 (100), 271 (7), 261 (16), 231 (10), 155 (31), 135 (51), 109 (52), 94 (52), 67 (38), 55 (59), 43 (53). HRMS Calculated for C_22_H_34_NaO_4_: 385.2355; found: 385.2358.

**Ozonolysis of ester 25 (preparation of 26):** To a solution of ester **25** (80 mg, 0.221 mmol) in dichloromethane at −78 °C was bubbled ozone at a rate as to not cause frothing. After 10 min the solution turned blue at which time ozone bubbling was ceased. The reaction was then stirred for a further 30 min before oxygen was bubbled through the solution until the blue colour dissipated. A solution of dimethyl sulfide (150 μL) in methanol (1 mL) was added and the reaction warmed to r.t. overnight. The volatiles were then removed *in vacuo* and the residue subjected to column chromatography (1:1 petroleum spirit/ethyl acetate) affording diketone **26** (40 mg, 50%) as a colourless oil. ^1^H NMR (400 MHz, CDCl_3_) δ 0.90 (s, 3H), 1.13 (s, 3H), 1.26 (s, 3H), 1.25−1.35 (m, 1H), 1.02−1.11 (m, 1H), 1.26 (t, *J* = 7.2 Hz, 3H), 1.47−1.67 (m, 3H), 1.80 (app. dt, *J* = 14.5, 2.5 Hz, 1H), 2.02 (s, 3H), 2.23−2.30 (m, 1H), 2.37 (d, *J* = 12.7 Hz, 1H), 2.48−2.55 (m, 1H), 2.74 (dd, *J* = 18.3, 7.2 Hz, 1H), 2.80−2.88 (m, 2H), 3.48 (ddd, *J =* 12.6, 7.1, 3.8 Hz, 1H), 3.73 (dd, *J* = 12.1, 5.2 Hz, 1H), 4.15 (q, *J* = 7.2 Hz, 2H), 4.31 (dd, *J* = 12.0, 4.5 Hz, 1H). ^13^C NMR (100 MHz, CDCl_3_) δ 14.3, 23.5, 25.8, 26.2, 29.3, 29.6, 32.9, 33.7, 33.9, 41.9, 45.4, 46.5, 47.0, 52.6, 56.4, 60.4, 61.3, 73.7, 174.1, 206.6, 212.2. GC/MS EI *m/z* (%) 364 (M^+^˙, 2), 349 (7), 318 (12), 306 (14), 288 (7), 263 (23), 232 (14), 212 (10), 166 (24), 123 (20), 111 (43), 94 (38), 67 (19), 55 (39), 44 (11), 43 (100). HRMS Calculated for C_21_H_32_NaO_5_: 387.2147; found: 387.2147.

**Silyl enol ether 27:** Ketone **23** (1.0 g, 3.25 mmol) and anhydrous triethylamine (1.2 mL, 8.45 mmol) were dissolved in anhydrous dichloromethane (35 mL) under an argon atmosphere and cooled to −78 °C. *tert*-Butyldimethylsilyl trifluoromethanesulfonate (970 μL, 4.23 mmol) was then added and the mixture stirred for 30 min, followed by stirring at 0 °C for 5 min. At this time TLC showed complete consumption of starting material. The reaction was then quenched by pouring the reaction contents into an ice-cold saturated solution of sodium hydrogen carbonate (20 mL) and extracted with diethyl ether (3 × 20 mL). The organic layer was partitioned, dried (Na_2_SO_4_) and evaporated *in vacuo*. Subjecting the residue to column chromatography (20:1 petroleum spirit/ethyl acetate) afforded the desired product **27** (1.17 g, 85%) and the corresponding regioisomer (113 mg, 8%). ^1^H NMR (400 MHz, CDCl_3_) δ 0.03 (s, 3H), 0.08 (s, 3H), 0.89 (s, 9H), 1.01 (td, *J* = 14.1, 4.3 Hz, 1H), 1.03 (s, 3H), 1.07 (s, 3H), 1.12 (dt, *J* = 12.3, 3.5 Hz, 1H),1.19−1.24 (m, 1H), 1.25 (t, *J* = 7.2 Hz, 3H), 1.26 (s, 3H), 1.34−1.38 (m, 1H), 1.56−1.63 (m, 1H), 1.97 (dq, *J* = 16.6, 4.3 Hz, 1H ) 2.07 (dd, *J* = 16.6, 4.3 Hz, 1H), 2.36 (dt, *J =* 14.3, 3.0 Hz, 1H), 2.63 (t, *J =* 4.61 Hz, 1H), 2.78−2.85 (m, 2H), 3.68 (dd, *J* = 11.8, 0.8 Hz, 1H), 4.13 (qd, *J* = 7.2, 1.0 Hz, 2H), 4.52 (d, *J* = 11.8 Hz, 1H). ^13^C NMR (100 MHz, CDCl_3_) δ −4.1, −4.0, 14.1, 18.1, 20.4, 23.7, 25.8 (3C), 27.2, 33.3, 33.4, 34.7, 36.4, 36.4, 41.6, 48.1, 53.8, 59.9, 61.3, 73.6, 113.2, 144.5, 174.2. GC/MS EI *m/z* (%) 422 (M^+^˙, 2), 407 (1), 365 (76), 347 (3), 291 (11), 265 (4), 245 (3), 199 (14), 157 (12), 105 (15), 75 (59), 73 (100), 43 (22). HRMS Calculated for C_24_H_42_NaSiO_4_: 445.2750; found: 445.2765.

**Compound 29 and 28:** To a solution of silyl enol ether **27** (432 mg, 1.02 mmol) in distilled acetone (35 mL) at −78 °C was added dimethyldioxirane (~0.08 M, 4.5 mmol, 57 mL) dropwise over 5 min. The reaction was monitored by TLC (8:1 petroleum spirit/ethyl acetate), which showed complete consumption of starting material after 5 min. Acetone and excess dimethyldioxirane were then removed *in vacuo* affording **29** which was not further purified due to facile decomposition. To the above residue (**29**) diluted in diethyl ether (150 mL) was added aqueous hydrochloric acid (1 M, 20 mL) and the mixture shaken vigorously for 90 s (i.e. separatory funnel). The aqueous phase was partitioned, extracted with diethyl ether (50 mL), and the combined ether layers washed with saturated sodium hydrogen carbonate solution (15 ml), dried (MgSO_4_) and concentrated *in vacuo*. The residue (crude **28**) was not further purified. Compound **29** ^1^H NMR (400 MHz, CDCl_3_) δ 0.08 (s, 3H), 0.17 (s, 3H), 0.80 (s, 3H), 0.86 (s, 9H), 0.95−1.09 (m, 1H), 1.14 (s, 3H), 1.18 (dd, *J* = 14.2, 4.1 Hz, 1H), 1.24 (t, *J* = 7.1 Hz, 3H), 1.34 (s, 3H), 1.44 (m, 1H), 1.60−1.66 (m, 2H), 1.75 (dt, *J* = 14.2, 2.6 Hz, 1H), 2.24−2.31 (m, 2H), 2.44 (ddd, *J* = 14.2, 5.9, 3.0 Hz, 1H), 2.68−2.76 (m, 2H), 3.64 (d, *J* = 10 Hz, 1H), 3.72 (d, *J* = 10 Hz, 1H), 4.11 (qd, *J* = 7.1, 0.5 Hz, 2H). ^13^C NMR (100 MHz, CDCl_3_) δ −3.5, −2.9, 14.2, 17.9, 22.0, 24.1, 25.8 (3C), 26.7, 31.6, 32.3, 32.7, 35.4, 35.8, 42.5, 46.8, 51.8, 60.1, 64.1, 66.2, 73.9, 87.6, 174.9. Compound **28** ^1^H NMR (400 MHz, C_6_D_6_) δ 0.27 (s, 3H), 0.32 (s, 3H), 0.95−1.00 (m, 1H), 0.98 (t, *J* = 7.1 Hz, 3H), 1.0 (s, 9H), 1.02 (s, 3H), 1.06 (s, 3H), 1.10−1.16 (m, 1H), 1.17 (s, 3H), 1.24 (dd, *J* = 14.5, 5.8, 1H), 1.28−1.35 (m, 1H), 1.61 (br s, OH), 2.25−2.21 (m, 1H), 2.33 (qd, *J* = 13.3, 3.9 Hz, 1H), 2.39−2.46 (m, 2H), 3.25 (d, *J* = 11.0 Hz, 1H), 3.35 (dd, *J* = 2.7, 1.3 Hz, 1H), 3.93−4.05 (m, 2H), 4.34 (d, *J* = 11.0 Hz, 1H), 5.20 (d, *J* = 2.7 Hz, 1H). ^13^C NMR (100 MHz, C_6_D_6_) δ −4.5, −4.0, 14.3, 18.5, 21.9, 24.4, 26.2 (3C), 27.4, 33.0, 33.1, 35.6, 39.1, 40.8, 44.5, 54.5, 60.2, 67.8, 72.9, 73.7, 110.0, 155.0, 174.6. GC/MS EI *m/z* (%) 438 (M^+^˙, 0.1), 420 (0.2), 405 (2), 363 (42), 347 (8), 289 (25), 215 (11), 197 (15), 133 (15), 75 (77), 73 (100), 43 (31), 41 (29). HRMS Calculated for C_24_H_42_NaO_5_Si: 461.2699; found: 461.2691.

**Compound 30:** Hydroxy ester **28** (806 mg, 1.84 mmol) was dissolved in anhydrous tetrahydrofuran (25 mL) and cooled to 0 °C under an argon atmosphere. To this was added sodium hydride (96 mg, 60% suspension in oil) portionwise. After 30 min the reaction was quenched by addition of ice-cold ammonium chloride (saturated aqueous solution). The residue was subjected to column chromatography (10:1 petroleum ether/ethyl acetate) which afforded the desired product **30** (636 mg, 79%) as a colourless oil. ^1^H NMR (400 MHz, C_6_D_6_) δ 0.06 (s, 3H), 0.11 (s, 3H), 0.85−0.91 (m, 1H), 0.93 (s, 9H), 0.94 (t, *J* = 7.1 Hz, 3H), 0.99 (s, 3H), 1.03 (s, 3H), 1.05−1.16 (m, 2H), 1.18 (s, 3H), 1.27−1.35 (m, 1H), 1.55 (qd, *J* = 13.9, 4.1 Hz, 1H), 2.14−2.23 (m, 2H), 2.29−2.33 (m, 1H), 2.72 (ddd, *J =* 11.2, 5.0, 0.7 Hz, 1H), 2.81 (dd, *J =* 15.7, 5.1 Hz, 1H), 3.26 (dd, *J =* 15.6, 11.3 Hz, 1H), 3.42 (d, *J* = 11.2 Hz, 1H), 3.84−3.98 (m, 2H), 4.28 (d, *J* = 11.2 Hz, 1H). ^13^C NMR (100 MHz, C_6_D_6_) δ −3.1, −2.0, 14.2, 18.7, 22.6, 24.0, 26.1 (3C), 27.0, 33.1, 33.8, 34.4, 39.9, 41.4, 41.5, 42.8, 51.5, 60.2, 67.4, 73.3, 79.5, 173.6, 205.8. GC/MS EI *m/z* (%) 438 (M^+^˙, 0.1), 423 (0.3), 381 (42), 363 (23), 335 (30), 307 (14), 289 (17), 261 (11), 215 (17), 187 (36), 135 (40), 75 (100), 73 (78), 41 (42). HRMS Calculated for C_24_H_42_NaO_5_Si: 461.2699; found: 461.2708.

**Compound 31:** Epoxide **29** (140 mg, 0.317 mmol) was dissolved in anhydrous dichloromethane (5 mL) and cooled to −78 °C under an argon atmosphere. To this was added hydrogen fluoride pyridine complex (200 μL) dropwise. After 30 min the reaction mixture was poured carefully into an ice-cold saturated solution of sodium hydrogen carbonate (10 mL) and extracted with diethyl ether (3 × 15 mL). The organic layer was then partitioned, dried (Na_2_SO_4_) and concentrated *in vacuo*. The residue was subjected to column chromatography (6:1 petroleum spirit/ethyl acetate) which afforded the desired material **31** (96 mg, 93%). ^1^H NMR (500 MHz, CDCl_3_) δ 0.97 (s, 3H), 1.06−1.13 (m, 1H), 1.21 (s, 3H), 1.25 (t, *J* = 7.1 Hz, 3H), 1.40 (s, 3H), 1.45 (dd, *J* = 15.2, 6.9 Hz, 1H), 1.55−1.80 (m, 3H), 1.92−1.97 (m, 1H), 2.01−2.11 (m, 1H), 2.39 (ddd, *J =* 13.6, 3.7, 1.5 Hz, 1H), 2.44−2.48 (m, 1H), 2.53 (dd, *J =* 11.8, 3.8 Hz, 1H), 3.65 (AB, *J* = 13.2 Hz, 2H), 3.68 (dd, *J =* 13.65, 11.76 Hz, 1H), 4.11−4.21 (m, 2H), 4.31 (s, OH). ^13^C NMR (100 MHz, CDCl_3_) δ 14.3, 22.7, 24.4, 26.6, 32.7, 33.5, 35.1, 35.3, 39.3, 40.0, 40.6, 52.8, 60.7, 67.3, 74.1, 77.0, 172.7, 211.7. HRMS Calculated for C_18_H_28_NaO_5_: 347.1829; found: 347.1821.

**Compound 32:** Hydroxy ester **31** (96 mg, 0.219 mmol) was dissolved in anhydrous tetrahydrofuran (5 ml) and cooled to 0 °C under an argon atmosphere. Sodium hydride (18 mg, 0.444 mmol, 60% suspension in oil) was added in one portion and the mixture stirred for 30 min. Excess methyl iodide (200 μL) was then added and the reaction stirred for 1 h before pouring into an ice-cold saturated solution of ammonium chloride (10 mL). After extraction with ether (3 × 15 mL) and drying (MgSO_4_) the combined organic layers were concentrated *in vacuo*. The residue was then subjected to column chromatography (4:1 petroleum spirit/ethyl acetate) which afforded compound **32** (54 mg, 51%) as colourless needles (mp 95 °C). ^1^H NMR (300 MHz, CDCl_3_) δ 0.90 (s, 3H), 1.02−1.12 (m, 1H), 1.09 (s, 3H), 1.13 (d, *J* = 7.0 Hz, 3H), 1.25 (t, *J* = 7.2 Hz, 3H), 1.29 (s, 3H), 1.31−1.44 (m, 3H), 1.49−1.61 (m, 1H), 2.19−2.34 (m, 2H), 2.43−2.48 (m, 1H), 2.51 (d, *J* = 12.2 Hz, 1H), 3.11 (s, 3H), 3.10−3.21 (m, 1H), 3.34 (d, *J* = 10.9 Hz, 1H), 4.08−4.18 (m, 2H), 4.26 (d, *J* = 10.9 Hz, 1H). ^13^C NMR (75 MHz, CDCl_3_) δ ^13^C NMR (75 MHz, CDCl_3_) δ 14.2, 22.0, 22.8, 24.3, 26.6, 32.3, 32.6, 33.5, 38.2, 42.7, 43.3, 45.8, 51.6, 58.9, 60.2, 64.2, 73.6, 81.2, 174.7, 208.8. HRMS Calculated for C_20_H_32_NaO_5_: 375.2142; found: 375.2126.

**Compound 33:** To a cold (0 °C) stirring solution of anhydrous diisopropylamine (48 μL, 0.35 mmol) in anhydrous tetrahydrofuran (1 mL) under an argon atmosphere, was added *n*-butyl lithium (1.39 M in hexanes, 227 μL, 0.32 mmol) dropwise. The above LDA solution was then added via syringe to an anhydrous tetrahydrofuran (3 mL) solution of ketone **30** (126 mg, 0.29 mmol) and TMSCl (44 μL, 0.35 mmol) under an argon atmosphere at −78 °C. The reaction was stirred for 1 h at −78 °C then quenched by pouring into ice-cold sodium hydrogen carbonate (saturated aqueous solution). The mixture was extracted with ether (2 × 15 mL), the combined organic layer dried (Na_2_SO_4_) and concentrated *in vacuo* to a give a colourless oil which was used without further purification. ^1^H NMR (300 MHz, C_6_D_6_) δ 0.20 (s, 3H), 0.22 (s, 3H), 0.32 (s, 9H), 0.95 (t, *J* = 7.1 Hz, 3H), 1.00 (s, 9H), 1.04−1.23(m, 2H), 1.26−1.38 (m, 2H), 1.07 (s, 3H), 1.09 (s, 3H), 1.19 (s, 3H), 2.25−2.52 (m, 3H), 2.64 (dt, *J* = 14.2, 2.4 Hz, 1H), 3.26 (d, *J* = 10.9 Hz, 1H), 3.48 (dd, *J* = 2.6, 1.3 Hz, 1H), 3.84−4.08 (m, 2H), 4.31 (d, *J* = 10.8 Hz, 1H), 5.17 (d, *J* = 2.4 Hz, 1H). ^13^C NMR (75 MHz, C_6_D_6_) δ −3.8, −2.2, 0.4 (3C), 14.3, 18.8, 22.0, 24.5, 26.1 (3C), 27.5, 33.0, 33.2, 35.7, 40.5, 41.1, 44.6, 54.9, 60.2, 68.6, 73.5, 75.3, 111.0, 154.3, 174.8.

**Compound 38:** To a solution of bromoacetaldehyde dimethyl acetal (65 mg, 0.29 mmol) in anhydrous dichloromethane (2 mL) was added freshly distilled titanium tetrachloride (40 μL, 0.36 mmol) dropwise via syringe. A solution of compound **33** (prepared above) in dichloromethane (2 mL) was then added dropwise over 2 min. The reaction was stirred at −78 °C for 4 h then quenched by pouring into an ice-cold saturated aqueous sodium hydrogen carbonate solution. The mixture was then extracted with ether (3 × 15 mL) and the combined organic layers were washed (brine) and dried (Na_2_SO_4_) then concentrated *in vacuo* to give a residue that was purified by flash chromatography (20:1 – 10:1 petroleum spirit/diethyl ether) to give **38** (13 mg, 9%) as a colourless oil. ^1^H NMR (400 MHz, CDCl_3_) δ 0.22 (s, 9H), 0.95 (s, 3H), 1.11 (s, 3H), 1.01−1.13 (m, 1H), 1.26 (t, *J* = 7.1 Hz, 3H), 1.33 (s, 3H), 1.37−1.57 (m, 3H), 2.11−2.19 (m, 1H), 2.22−2.28 (m, 1H), 2.53−2.59 (m, 2H), 3.23 (dd, *J =* 2.8, 1.3 Hz, 1H), 3.50 (d, *J* = 11.1 Hz, 1H), 4.10−4.22 (m, 2H), 4.30 (d, *J* = 11.3 Hz, 1H), 4.95 (d, *J* = 4.95 Hz, 1H). ^13^C NMR (100 MHz, CDCl_3_) δ 0.4 (3C), 14.3, 21.5, 24.2, 27.2, 32.3, 32.7, 35.3, 40.6, 42.4, 46.4, 54.0, 60.4, 68.5, 72.4, 74.3, 111.3, 151.8, 174.6. GC/MS EI *m/z* (%) 378 (M^+^˙−18, 8), 363 (7), 349 (3), 320 (3), 305 (12), 247 (37), 157 (11), 129 (8), 91 (12), 73 (100), 45 (26).

**Alkylation of ketone 30 with propargyl bromide (preparation of 39):** To a cold (0 °C) stirring solution of anhydrous diisopropylamine (116 μL, 0.827 mmol) in anhydrous tetrahydrofuran (4 mL) under an argon atmosphere, was added *n*-butyl lithium (1.6 M in hexanes, 473 μL, 0.76 mmol) dropwise. The above LDA solution was then added via cannula to a −78 °C anhydrous tetrahydrofuran (20 mL) solution of ketone **30** (302 mg, 0.689 mmol). The reaction mixture was then stirred for 30 min followed by addition of HMPA (250 μL) and propargyl bromide (80% in toluene, 61 μL, 0.689 mmol). After 4 h at −78 °C the reaction was allowed to warm to r.t. overnight. The reaction was quenched by pouring into ice-cold ammonium chloride (saturated aqueous solution) then extraction with ether (3 × 20 mL), followed by washing with brine, drying (Na_2_SO_4_) and evaporation resulted in an oily residue. Column chromatography (10:1 petroleum spirit/diethyl ether) afforded compound **39** (162 mg, 49%) as a white powder (mp 87−89 °C). ^1^H NMR (400 MHz, CDCl_3_) δ ^1^H NMR (400 MHz, CDCl_3_) δ −0.02 (s, 3H), 0.21 (s, 3H), 0.85 (s, 9H), 0.91 (s, 3H), 0.97−1.08 (m, 1H), 1.23 (s, 3H), 1.26 (t, *J* = 7.1 Hz, 3H), 1.42 (s, 3H), 1.42 (dd, *J* = 15.1, 7.0 Hz, 1H), 1.55−1.68 (m, 3H), 1.87 (t, *J* = 2.6 Hz, 1H), 1.88−1.93 (m, 1H), 2.03−2.18 (m, 3H), 2.44−2.48 (m, 1H), 2.52 (ddd, *J* = 15.8, 11.0, 2.6 Hz, 1H), 3.66 (d, *J* = 12.9 Hz, 1H), 3.96 (ddd, *J =* 12.4, 11.1, 3.3 Hz, 1H), 4.16 (q, *J* = 7.1 Hz, 2H), 4.16 (d, *J* = 12.9 Hz, 1H). ^13^C NMR (100 MHz, CDCl_3_) δ −2.8, −2.4, 14.3, 18.8, 20.6, 22.5, 24.2, 26.2 (3C), 26.9, 32.8, 34.1, 35.9, 37.9, 39.3, 40.1, 46.9, 58.5, 60.6, 66.5, 69.7, 73.5, 80.6, 82.0, 172.1, 208.5. GC/MS EI *m/z* (%) 461 (M^+^˙−15, 0.4), 444 (0.2), 419 (25), 401 (6), 345 (9), 327 (11), 253 (8), 193 (13), 135 (19), 93 (18), 75 (100), 41 (39). HRMS Calculated for C_27_H_44_NaO_5_Si: 499.2856; found: 499.2856.

**Reaction of compound 39 with mercury triflate (preparation of 40 and 41):** Compound **39** (122 mg, 0.256 mmol) dissolved in anhydrous benzene (2 mL) under an argon atmosphere was added to an anhydous benzene solution of mercury triflate (0.01 M, 256 μL), prepared according to Imagawa et al. [2] and stirred for 30 min. The reaction was quenched by addition of sodium hydrogen carbonate (saturated solution, 5 mL) Extraction with dichloromethane (2 × 15 mL), followed by washing with brine, drying (Na_2_SO_4_) and evaporation resulted in an oily residue. Column chromatography (10:1 – 5:1 petroleum spirit/ethyl acetate) afforded diketone **40** (40 mg, 31%) and furan **41** (12 mg, 13%) both as colourless oils. Diketone **40**. ^1^H NMR (300 MHz, CDCl_3_) δ −0.08 (s, 3H), 0.13 (s, 3H), 0.85 (s, 9H), 0.90 (s, 3H), 0.94−1.09 (m, 1H), 1.23 (s, 3H), 1.28 (t, *J* = 7.1 Hz, 3H), 1.37 (s, 3H), 1.42 (dd, *J* = 15.1, 6.7 Hz, 1H), 1.55−1.70 (m, 4H), 1.92−2.06 (m, 1H), 2.05 (s, 3H), 2.09 (d, *J* = 12.1 Hz, 1H), 2.42−2.32 (m, 2H), 3.04 (dd, *J* = 17.7, 10.6 Hz, 1H), 3.67 (d, *J* = 12.8 Hz, 1H), 3.92 (ddd, *J =* 12.6, 10.7, 3.2 Hz, 1H), 4.17 (q, *J* = 7.2 Hz, 2H), 4.50 (d, *J* = 12.9 Hz, 1H). ^13^C NMR (75 MHz,) δ −3.0, −2.4, 14.3, 18.8, 23.4, 26.0, 26.1 (3C), 26.5, 29.5, 33.1, 33.8, 35.6, 38.3, 40.1, 40.5, 43.1, 46.7, 58.1, 60.5, 67.4, 73.7, 83.5, 172.7, 205.8, 210.1. GC/MS EI *m/z* (%) 479 (M^+^˙−15, 0.2), 462 (0.1), 437 (9), 419 (12), 373 (13), 345 (22), 327 (16), 271 (12), 253 (7), 167 (12), 75 (90), 73 (47), 43 (100). HRMS Calculated for C_27_H_46_NaO_6_Si: 517.2961; found: 517.2965. Furan **41**. ^1^H NMR (500 MHz, C_6_D_6_) δ 0.97 (t, *J* = 7.1 Hz, 3H), 1.06−1.15 (m, 2H), 1.13 (s, 3H), 1.15 (s, 3H), 1.20 (s, 3H), 1.26−1.35 (m ,1H), 1.27−1.36 (m, 1H), 1.78 (dtd, *J* = 12.1, 7.5, 4.6 Hz, 1H), 1.99 (dd, *J* = 14.1, 4.2 Hz, 1H), 2.01 (d, *J* = 0.65 Hz, 3H), 2.38 (ddd, J = 14.1, 9.2, 4.6 Hz, 1H), 2.68−2.58 (m, 1H), 3.62 (s, 1H), 3.99 (m, 2H), 5.78 (d, J = 0.8 Hz, 1H), 6.79 (d, *J* = 2.7 Hz, 1H). ^13^C NMR (125 MHz, C_6_D_6_) δ 13.4, 14.4, 19.7, 25.9, 28.9, 31.0, 32.4, 33.2, 33.8, 39.8, 42.0, 55.5, 60.1, 77.9, 107.7, 109.3, 116.0, 144.0, 146.8, 149.4, 172.5. GC/MS EI *m/z* (%) 344 (M^+^˙, 100), 315 (15), 271 (25), 241 (14), 227 (15), 187 (48), 173 (23), 159 (30), 145 (22), 91 (25), 55 (25), 43 (96), 41 (55).

**Allylation of ketone 30 (preparation of 43 and 44):** To a cold (0 °C) stirring solution of anhydrous diisopropylamine (85 μL, 0.61 mmol) in anhydrous tetrahydrofuran (2 mL) under an argon atmosphere, was added *n*-butyllithium (1.55 M in hexanes, 401 μL, 0.557 mmol) dropwise. The above LDA solution was then added via syringe to a −78 °C anhydrous tetrahydrofuran (10 mL) solution of ketone **30** (222 mg, 0.51 mmol) under an argon atmosphere. The reaction mixture was then stirred for 30 min followed by addition of allyl bromide (45 μL, 0.53 mmol). After 4 h at −78 °C the reaction was allowed to warm to r.t. overnight. The reaction was quenched by pouring onto an ice-cold saturated solution of sodium hydrogen carbonate then the mixture was extracted with ether (3 × 20 mL). The combined organic layer was washed with brine, dried (Na_2_SO_4_) and concentrated *in vacuo* to afford an oily residue. Column chromatography (20:1 – 10:1 petroleum spirit/ethyl acetate) afforded C-allylated material **43** as a colourless oil (38 mg, 15%), O-allylated material **44** as a colourless oil (50 mg, 21%) and recovered starting material **30** (70 mg). **Conversion of 44 into 43:** A solution of O-allylated material **44** (50 mg) in dry toluene (1 mL) under an argon atmosphere was heated under microwave irradiation for 20 min (maximum temperature 170 °C, 300 W). The solvent was removed *in vacuo* affording C-allylated material **43** (50 mg, 100%) as a colourless oil. Compound **43** ^1^H NMR (300 MHz, CDCl_3_) δ −0.073 (s, 3H), 0.19 (s, 3H), 0.84 (s, 9H), 0.90 (s, 3H), 0.94−1.08 (m, 1H), 1.22 (s, 3H), 1.27 (t, *J* = 7.1 Hz, 3H), 1.36−1.43 (m, 1H), 1.40 (s, 3H), 1.67−1.52 (m, 3H), 1.91−2.19 (m, 3H), 2.23 (d, *J* = 11.4 Hz, 1H), 2.37−2.50 (m, 2H), 3.58 (d, *J* = 12.8 Hz, 1H), 3.72 (ddd, *J* = 12.5, 10.7, 3.0 Hz, 1H), 3.78 (d, *J* = 12.8 Hz, 1H), 4.16 (q, *J* = 7.1 Hz, 2H), 4.91−5.04 (m, 2H), 5.60 (dddd, *J =* 17.0, 10.0, 8.4, 6.0 Hz, 1H). ^13^C NMR (75 MHz, CDCl_3_) δ −2.6, −2.3, 14.3, 18.7, 22.5, 24.2, 26.2 (3C), 26.8, 32.8, 33.9, 35.4, 35.9, 38.0, 39.4, 40.2, 47.5, 58.9, 60.3, 66.5, 73.6, 80.6, 117.9, 135.1, 172.7, 209.1. HRMS Calculated for C_27_H_46_KO_5_Si: 517.2752; found: 517.2769. Compound **44** ^1^H NMR (300 MHz, CDCl_3_) δ −0.06 (s, 3H), −0.02 (s, 3H), 0.83 (s, 9H), 0.88 (s, 3H), 0.98−1.10 (m, 1H), 1.08 (s, 3H), 1.27 (t, *J* = 7.1 Hz, 3H), 1.31 (s, 3H), 1.34−1.44 (m, 3H), 2.05−2.20 (m, 1H), 2.22−2.30 (m, 2H), 2.47 (dt, *J =* 14.1, 2.4, 1H), 3.12 (d, *J =* 10.7 Hz, 1H), 3.19 (dd, *J =* 2.7, 1.1 Hz, 1H), 4.06−4.26 (m, 4H), 4.31 (d, *J =* 10.8 Hz, 1H), 4.59 (d, *J =* 2.8 Hz, 1H), 5.17 (dq, *J =* 10.5, 1.4 Hz, 1H), 5.32 (dq, *J =* 17.3, 1.6 Hz, 1H), 6.05−5.91 (m, 1H). ^13^C NMR (100 MHz, CDCl_3_) δ −4.1, −2.5, 14.3, 18.4, 22.3, 24.4, 25.8 (3C), 27.1, 32.4, 32.5, 34.6, 40.7, 42.0, 44.6, 54.0, 60.2, 67.7, 68.2, 73.5, 74.7, 101.1, 116.9, 133.4, 157.2, 175.7. GC/MS EI *m/z* (%) 463 (M^+^˙−15, 2), 421 (8), 403 (5), 375 (10), 347 (15), 329 (14), 255 (19), 237 (21), 227 (15), 135 (21), 75 (100). HRMS Calculated for C_27_H_46_NaO_5_Si: 501.3007; found: 501.3000.

**Wacker oxidation of 43 (preparation of 40):** Compound **43** (15 mg, 0.031 mmol) was dissolved in *N*,*N*-dimethylformamide and water (7:1, 1 mL), and to this was added palladium dichloride (~0.5 mg, 10%) and copper(I) chloride (3 mg, 0.031 mmol). The reaction mixture was then stirred under an oxygen atmosphere for 48 h. Filtration through celite followed by evaporation under high vacuum gave a residue which was subjected to column chromatography (5:1 petroleum spirit/ethyl acetate) affording diketone **40** (10 mg, 64%). Spectral data were identical to that described above.

**Reduction/oxidation of ketone 23 (preparation of 46):** Ketone **23** (118 mg, 0.383 mmol) was dissolved in anhydrous tetrahydrofuran (5 mL) under an argon atmosphere. To this was added lithium aluminium hydride (30 mg, 0.766 mmol) and the reaction stirred at 0 °C for 30 min then warmed to room temperature over 2 h. The reaction was quenched by cautious addition of sodium sulfate decahydrate (~500 mg) then stirred for 30 min. The white mixture was diluted with ether (50 mL) and filtered through celite. The filter cake was washed with a further 50 mL then evaporation resulted in an oily residue, which was not further purified. To a solution of oxalyl chloride (331 μL, 3.83 mmol) in anhydrous dichloromethane (10 mL) under an argon atmosphere at −78 °C was added dimethyl sulfoxide (380 μL, 5.36 mmol) dropwise. After 5 min at −78 °C the above crude (dried under high vacuum) dissolved in anhydrous dichloromethane (2 mL) was added. After 1 h at −78 °C excess anhydrous triethylamine (2.66 mL, 19.0 mmol) was added and stirring continued at that temperature for a further 15 min. The solution was warmed to 0 °C and stirred for 20 min, then diluted with diethyl ether (30 mL), washed with brine (10 mL) and dried (MgSO_4_). Evaporation *in vacuo* gave a residue which was subjected to column chromatography (1:1 petroleum spirit/ethyl acetate) affording compound **46** (78 mg, 77% over two steps). ^1^H NMR (400 MHz, CDCl_3_) δ 1.11 (s, 3H), 1.17 (s, 3H), 1.18−1.25 (m, 3H), 1.27 (s, 3H), 1.53–1.57 (m, 1H), 1.66 (dd, *J =* 14.8, 5.7 Hz, 1H), 1.87–1.94 (m, 1H), 2.16 (dt, *J =* 14.8, 2.5 Hz, 1H), 2.33–2.37 (m, 1H), 2.44 (td, *J =* 6.4, 3.5 Hz, 1H), 2.63–2,69 (m, 1H), 2.77 (ddd, *J =* 13.9, 6.3, 0.6 Hz, 1H), 2.87 (dd, *J =* 13.9, 6.6 Hz, 1H), 3.64 (dd, *J =* 12.1, 3.8 Hz, 1H), 4.21 (dd, *J =* 12.1, 1.2 Hz, 1H), 9.80 (d, *J* = 3.5 Hz, 1H). ^13^C NMR (100 MHz, CDCl_3_) δ 21.1, 23.4, 27.6, 30.2, 34.4, 35.1, 35.2, 41.5, 43.1, 44.1, 51.3, 55.1, 60.2, 73.4, 202.9, 210.9. GC/MS EI *m/z* (%) 264 (M^+^˙, 1), 249 (5), 221 (11), 206 (16), 177 (9), 146 (29), 131 (16), 119 (17), 94 (13), 79 (72), 55 (46), 41 (64).

**Wittig product 47:** [(3-Methylbut-2-enoyloxy)methyl]triphenylphosphonium chloride: To a solution of (hydroxymethyl)triphenylphosphonium chloride [3] (13.8 g, 42 mmol) in anhydrous dichloromethane (10 mL) under an argon atmosphere was added 3,3-dimethylacryloyl chloride (10 g, 85 mmol) dropwise. The mixture was refluxed for 16 h, cooled to room temperature and transferred by pipette to vigorously stirred diethyl ether (50 mL). A white gum formed which was isolated by decanting off the mother liquor then re-dissolved in dichloromethane (25 mL). The trituration process was repeated with diethyl ether / dichloromethane until pure by ^1^H NMR and then the product was placed under high vacuum (0.05 mmHg) for 48 h which afforded [(3-methylbut-2-enoyloxy)methyl]triphenylphosphonium chloride as white powder (15.8 g, 92%). [Note: highly hygroscopic.] ^1^H NMR (400 MHz, CDCl_3_) *δ* 1.82 (d, *J =* 1.2 Hz, 3H), 1.94 (d, *J* = 1.2 Hz, 3H), 5.53 (sept, *J* = 1.2 Hz, 1H), 6.53 (d, *J* = 2.8 Hz, 2H), 7.64−7.72 (m, 6H), 7.77–7.86 (m, 9H). ^13^C NMR (100 MHz, CDCl_3_) *δ* 20.6, 27.5, 55.7 (d, *J* = 65 Hz), 113.2, 115.9 (d, 86 Hz, 3C), 130.5 (d, *J* = 13 Hz, 6C), 134.2 (d, *J* = 10 Hz, 6C), 135.6 (d, *J* = 3 Hz, 3C), 162.0, 163.6 (d, *J* = 4.6 Hz).

A suspension of [(3-methylbut-2-enoyloxy)methyl]triphenylphosphonium chloride (385 mg, 0.937 mmol) in anhydrous tetrahydrofuran (10 mL) under an argon atmosphere was sonicated for 10 min until a uniform milky dispersion had formed. The mixture was then cooled to −61 °C (CHCl_3_/CO_2_ bath) and a solution of lithium diisopropylamide was added strictly dropwise. [Lithium diisopropylamide prepared as follows: To a cold (0 °C) stirring solution of anhydrous diisopropylamine (71 μL, 0.508 mmol) in anhydrous tetrahydrofuran (8.5 mL) under an argon atmosphere, was added *n*-butyllithium (1.5 M in hexanes, 401 μL, 0.310 mmol) dropwise.] The red mixture was stirred for 45 min at −61°C then a solution of aldehyde **46** (71 mg, 0.269 mmol) in anhydrous tetrahydrofuran (2 mL) was added dropwise and stirred for a further 4 h at −61°C. The mixture was then poured onto saturated ice-cold sodium bicarbonate (10 mL) and extracted with diethyl ether (3 × 15 mL). The combined organic layer was washed with brine, dried over sodium sulfate and concentrated *in vacuo*. The residue was purified by column chromatography (10:1, petroleum spirit/ethyl acetate) to give the desired product **47** as a colourless oil (20 mg, 21%). ^1^H NMR (400 MHz, C_6_D_6_, mixture of *cis* and *trans* isomers) δ 0.56–0.76 (m, 4H), 0.77 (s, 3H), 1.00 (s, 3H), 1.05 (s, 3H), 1.06 (s, 6H), 1.10 (s, 3H), 1.14–1.29 (m, 4H), 1.378 (s, 3H), 1.381 (s, 3H), 1.55–1.59 (m, 1H), 1.62–1.80 (m, 6H), 1.93–2.00 (m, 2H), 2.01–2.10 (m, 3H), 2.04 (d, *J* = 1.6Hz, 3H), 2.06 (d, *J* = 1.6Hz, 3H), 2.21 (dd, *J* = 12, 4.8Hz, 1H), 2.32 (dd, *J* = 13, 6Hz, 1H), 2.48 (dd, *J* = 12, 6.8Hz, 1H), 2.56 (dd, *J* = 12, 6Hz, 1H), 2.98–3.05 (m, 1H), 3.27 (dd, *J* = 12, 3.6Hz, 1H), 3.32 (dd, *J* = 12, 4Hz, 1H), 4.35 (d, *J* = 12Hz, 1H), 4.96 (dd, *J* = 11, 6.4Hz, 1H), 5.66 (sept, *J* = 1.2Hz, 1H), 5.67 (sept, *J* = 1.2Hz, 1H), 5.76 (dd, *J* = 12, 11Hz, 1H), 7.38 (dd, *J* = 6.4, 0.8Hz, 1H), 7.49 (d, *J* = 12Hz, 1H). ^13^C NMR (100 MHz, C_6_D_6_, mixture of *cis* and *trans* isomers) δ 20.3, 21.0, 21.5, 23.3, 23.5, 27.0, 28.1, 30.3, 30.4, 34.8, 34.9, 35.1, 35.8, 39.9, 42.4, 43.3, 43.4, 43.5, 43.7, 47.4, 48.0, 50.7, 50.9, 59.7, 60.1, 72.7, 72.8, 114.0, 115.0, 115.2, 115.4, 134.2, 135.9, 159.2, 159.6, 162.9, 163.4, 209.6, 209.8. GC/MS EI *m/z* (%) 360 (M^+^˙,0.1), 345 (0.1), 277 (1), 260 (1), 135 (1), 83 (100), 55 (19). HRMS Calculated for C_22_H_32_NaO_4_: 383.2198; found: 383.2203.

**Reduction/oxidation of silyl enol ether 27 (preparation of 48):** Silyl enol ether **27** (296 mg, 0.701 mmol) was dissolved in anhydrous dichloromethane (20 mL) under an argon atmosphere and cooled to −78 °C. To this was added diisobutylaluminium hydride (2.81 mL, 1.0 M, 2.81 mmol) dropwise. The reaction was stirred at −78 °C for 1 h then quenched by addition of wet silica (1 g, 1:3 water:silica). The mixture was then filtered and the filter cake washed with dichloromethane (50 mL). The solution was concentrated *in vacuo* to give a residue that was used without further purification. The above crude (dried under high vacuum) was dissolved in anhydrous dichloromethane (15 mL) under an argon atmosphere and powdered molecular sieves (4 Å, 500 mg) added followed by *N*-methyl-morpholine-*N*-oxide (205 mg, 1.75 mmol) and TPAP (10 mg). After 20 min the reaction was complete (TLC). The reaction mixture was then filtered through celite and evaporated *in vacuo*. The residue was subjected to column chromatography (15:1 petroleum spirit/ethyl acetate) affording aldehyde **48** (232 mg, 88% over two steps) as a white solid (mp 137–139 °C). ^1^H NMR (400 MHz, CDCl_3_) δ 0.04 (s, 3H), 0.08 (s, 3H), 0.90 (s, 9H), 1.05 (s, 3H), 1.07 (s, 3H), 1.08–1.32 (m, 3H), 1.29 (s, 3H), 1.47–1.54 (m, 1H), 1.97–2.07 (m, 2H), 2.29–2.33 (m, 1H), 2.49 (dt, *J =* 14.37, 3.16 Hz, 1H), 2.89 (br s, 1H), 3.00–3.08 (m, 1H), 3.72 (ddd, *J =* 12.1, 1.8, 1.1 Hz, 1H), 4.52 (d, *J* = 12.1 Hz, 1H), 4.52 (d, *J* = 12.1 Hz, 1H), 9.98 (d, *J* = 3.0 Hz, 1H). ^13^C NMR (100 MHz, CDCl_3_) δ −4.3, −3.9, 18.0, 21.2, 23.4, 25.7 (3C), 27.3, 31.5, 34.4, 34.5, 35.9, 36.4, 41.0, 46.7, 59.9, 60.8, 73.7, 114.7, 143.8, 205.9. GC/MS EI *m/z* (%) 380 (M^+^˙,6), 363 (1), 349 (1), 321 (19), 303 (12), 263 (14), 207 (15), 169 (19), 95 (29), 91 (19), 75 (77), 73 (100), 59 (23), 55 (21), 41 (40). HRMS Calculated for C_22_H_38_NaO_3_Si: 401.2488; found: 401.2494.

**Wittig product 49:** A suspension of [(3-methylbut-2-enoyloxy)methyl]triphenylphosphonium chloride (478 mg, 1.17 mmol) in anhydrous tetrahydrofuran (10 mL) under an argon atmosphere was sonicated for 10 min until a uniform milky dispersion had formed. The mixture was then cooled to −61 °C (CHCl_3_/CO_2_ bath) and a solution of lithium diisopropylamide was added strictly dropwise. [Lithium diisopropylamide prepared as follows: To a cold (0 °C) stirring solution of anhydrous diisopropylamine (157 μL, 1.12 mmol) in anhydrous tetrahydrofuran (2 mL) under an argon atmosphere, was added *n*-butyl lithium (1.6 M in hexanes, 656 μL, 1.05 mmol) dropwise] The red mixture was stirred for 45 min at −61 °C then a solution of aldehyde **48** (132 mg, 0.35 mmol) in anhydrous tetrahydrofuran (2 mL) was added dropwise and stirred for a further 90 min at −61 °C. The mixture was then poured onto saturated ice-cold sodium bicarbonate (10 mL) and extracted with diethyl ether (3 × 15 mL). The combined organic layer was washed with brine, dried over sodium sulfate and concentrated *in vacuo*. The residue was purified by column chromatography (20:1 petroleum spirit/ethyl acetate) to give the desired product **49** as a colourless oil (53 mg, 32%). ^1^H NMR: (400 MHz, C_6_D_6_, mixture of *cis* and *trans* isomers) δ 0.02 (s, 3H), 0.05 (s, 3H), 0.06 (s, 3H), 0.12 (s, 3H), 0.72–1.03 (m, 15H), 0.83 (s, 3H), 0.93 (s, 18H), 1.10 (s, 3H), 1.15 (d, *J* = 3.2Hz, 3H), 1.13 (s, 3H), 1.25 (s, 3H), 1.36 (d, *J* = 1.6Hz, 3H), 1.37 (d, *J* = 1.6Hz, 3H), 1.69–1.81 (m, 2H), 1.86–1.94 (m, 1H), 2.03 (d, *J* = 1.2Hz, 3H), 2.04 (d, *J* = 1.2Hz, 3H), 2.23 (dt, *J* = 10.8, 4.0Hz, 1H), 2.30 (dt, *J* = 14, 3.2Hz, 1H), 2.39 (dt, *J* = 14, 3.2Hz, 1H), 2.71 (bs, 1H), 2.74 (bs, 1H), 3.01–3.08 (m, 1H), 3.13–3.19 (m, 1H), 3.82 (bd, *J* = 11.6Hz, 1H), 3.85 (bd, *J* = 12Hz, 1H), 4.86 (bd, *J* = 12Hz, 1H), 4.87 (bd, *J* = 12Hz, 1H), 5.43 (dd, *J* = 10, 6.4Hz, 1H), 5.62 (sept, *J* = 1.2Hz, 1H), 5.70 (sept, *J* = 1.2Hz, 1H), 6.20 (dd, *J* = 12, 11Hz, 1H), 7.40 (dd, *J* = 6.4, 0.8Hz, 1H), 7.56 (d, *J* = 12Hz, 1H). ^13^C NMR (100 MHz, C_6_D_6_, mixture of *cis* and *trans* isomers) δ −4.1, −3.9, −3.6, 18.2, 18.3, 20.2, 20.3, 21.7, 22.2, 23.6, 25.9 (3C), 27.0, 27.7, 27.8, 34.0, 34.1, 35.0, 36.1, 36.4, 36.9, 37.2, 37.6, 38.0, 40.9, 41.3, 44.9, 47.3, 47.4, 48.6, 61.0, 73.5, 73.6, 115.2, 115.3, 115.5, 115.6, 116.0, 134.3, 136.2, 144.1, 144.3, 158.7, 159.3, 162.9, 163.4. GC/MS EI *m/z* (%) 474 (M^+^˙, 3) 459 (0.2), 417 (1), 391 (10), 363 (3), 317 (3), 238 (4), 181 (4), 157 (11), 83 (100), 73 (37), 55 (17).

**Deprotection of 49 (preparation of 47):** Silyl enol ether **49** (19 mg, 0.040 mmol) was dissolved in anhydrous dichloromethane (2 mL) under an argon atmosphere and cooled to −78 °C. To this was added hydrogen fluoride pyridine complex (100 μL) and the reaction stirred for 10 min. The reaction was quenched by cautious addition of saturated sodium hydrogen carbonate solution (5 mL) and then the mixture was extracted with dichloromethane (2 × 15 mL). The combined organic layer was washed with brine, dried (Na_2_SO_4_) and concentrated *in vacuo* to afford an oil. Column chromatography (10:1 petroleum spirit/ethyl acetate) afforded ketone **47** as a colourless oil (13 mg, 92%). Spectral data was identical to that reported above.

**Reduction/oxidation of diketone 26 (preparation of 52):** Diketone **26** (5 mg, 0.014 mmol) was dissolved in anhydrous tetrahydrofuran (2 mL) under an argon atmosphere. To this was added lithium aluminium hydride (5 mg, 0.131 mmol) and the reaction stirred at r.t. for 1.5 h. The reaction was quenched by addition of sodium sulfate decahydrate (50 mg) and this mixture was stirred for 45 min. The salts were filtered through celite, washed with ethyl acetate (20 mL) and the filtrate evaporated *in vacuo* affording an oily residue, which was not further purified.

To a solution of oxalyl chloride (27 μL, 0.031 mmol) in anhydrous dichloromethane (1 mL) under an argon atmosphere at −78 °C was added dimethyl sulfoxide (28 μL, 0.40 mmol) dropwise. After 5 min at −78 °C the above crude (dried under high vacuum) dissolved in anhydrous dichloromethane (1 mL) under an argon atmosphere was added. After 1 h at −78 °C excess anhydrous triethylamine (105 μL, 0.76 mmol) was added and stirring continued at that temperature for a further 15 min. The solution was warmed to 0 °C and stirred for 20 min, then diluted with diethyl ether (20 mL), washed with brine (10 mL) and dried (MgSO_4_). Evaporation *in vacuo* gave a residue which was subjected to column chromatography (1:5 – 1:1, petroleum spirit/ethyl acetate) affording compound **52** (~1 mg, >10% over two steps). ^1^H NMR (400 MHz, CDCl_3_) δ 1.00 (s, 3H), 1.16-1.26 (m, 2H), 1.19 (s, 3H), 1.29 (s, 3H), 1.34-1.44 (m, 1H), 1.59 (dd, *J* = 14.6, 5.9 Hz, 1H), 1.70-1.82 (m, 2H), 1.94-2.00 (m, 1H), 2.04 (s, 3H), 2.11 (ddd, *J =* 12.4, 4.4, 1.3 Hz, 1H), 2.52-2.58 (m, 1H), 2.60 (dd, *J =* 18.3, 3.6 Hz, 1H), 2.81 (dd, *J =* 18.2, 8.5 Hz, 1H), 2.88 (ddd, *J =* 9.0, 6.2, 4.4 Hz, 1H), 3.78 (ddd, *J =* 12.2, 8.6, 3.7 Hz, 1H), 3.86 (dd, *J =* 12.5, 6.2 Hz, 1H), 4.29 (dd, *J =* 12.5, 4.4 Hz, 1H), 9.70 (d, *J* = 4.4 Hz, 1H). GC/MS EI *m/z* (%) 320 (M^+^˙, 0.5), 305 (1), 281 (2), 274 (3), 262 (3), 233 (12), 207 (17), 121 (8), 94 (39), 79 (21), 44 (82), 40 (100).

**Silyl enol ether 53:** Diketone **40** (25 mg, 0.051 mmol) was dissolved in anhydrous dichloromethane (2 mL) under an argon atmosphere and cooled to −78 °C. TBSOTf (35 μL, 0.152 mmol) and anhydrous triethylamine (15 μL, 0.11 mmol) were then added and the reaction allowed to warm to r.t. over 2 h. The reaction was quenched by addition of saturated sodium hydrogen carbonate (5 mL) and then the mixture was extracted with dichloromethane (3 × 10 mL). The combined organic phase was washed with brine, dried (Na_2_SO_4_) and concentrated *in vacuo* which afforded an oil that was purified by column chromatography (20:1 petroleum spirit/ethyl acetate) to give **53** as a colourless oil (20 mg, 55%) and recovered starting material (10 mg). ^1^H NMR (400 MHz, CDCl_3_) δ 0.18 (s, 3H), 0.19 (s, 3H), 0.23 (s, 3H), 0.28 (s, 3H), 0.85 (s, 3H), 0.90 (s, 9H), 0.93–0.97 (m, 1H), 0.94 (s, 9H), 0.95 (t, *J* = 7.1 Hz, 3H), 1.07–1.25 (m, 3H), 1.16 (s, 3H), 1.31 (s, 3H), 1.53–1.45 (m, 1H), 2.06–2.14 (m, 2H), 2.26–2.45 (m, 3H), 2.85 (dd, *J* = 11.4, 11.5 Hz, 1H), 3.78 (d, *J* = 12.8 Hz, 1H), 3.95–3.86 (m, 2H), 4.13 (s, 1H), 4.14 (d, *J* = 12.8 Hz, 1H), 4.24 (s, 1H), 4.25-4.31 (m, 1H). ^13^C NMR (100 MHz, CDCl_3_) δ −5.1, −4.6, −2.2, −1.7, 14.3, 18.2, 19.2, 22.9, 24.4, 25.9 (3C), 25.9 (3C), 26.8, 33.2, 34.1, 36.2, 38.4, 39.1, 39.3, 40.3, 45.7, 59.3, 60.2, 67.0, 73.3, 81.6, 93.3, 156.5, 172.3, 209.2.

**Reduction/oxidation of ketone 43 (preparation of 55):** Ketone **43** (105 mg, 0.22 mmol) was dissolved in anhydrous tetrahydrofuran (5 mL) under an argon atmosphere and cooled to 0 °C. To this was added lithium aluminium hydride (33 mg, 0.879 mmol) and the reaction allowed to warm to r.t.. The reaction was quenched by addition of sodium sulfate decahydrate (500 mg) and then the mixture was stirred for 45 min. The salts were filtered through celite, washed with ethyl acetate (20 mL) and the filtrate evaporated *in vacuo* which afforded a residue which was not further purified.

To a solution of trifluoroacetic anhydride (52 μL, 0.374 mmol) in anhydrous dichloromethane (1 mL) under an argon atmosphere at −78 °C was added dimethyl sulfoxide (40 μL, 0.51 mmol) dropwise. After 5 min at −78 °C the above crude (dried under high vacuum) dissolved in anhydrous dichloromethane (2 mL) under an argon atmosphere was added. After 1 h at −78 °C excess anhydrous triethylamine (185 μL, 1.3 mmol) was added and stirring continued at that temperature for a further 15 min. The solution was warmed to 0 °C and stirred for 20 min, then diluted with diethyl ether (20 mL), washed with brine (15 mL) and dried (MgSO_4_). Evaporation *in vacuo* gave a residue which was subjected to column chromatography (10:1 petroleum spirit/ethyl acetate) affording compound **55** (5 mg, 5% over two steps). ^1^H NMR (400 MHz, CDCl_3_) δ −0.07 (s, 3H), 0.19 (s, 3H), 0.85 (s, 9H), 0.96 (s, 3H), 1.14–1.05 (m, 1H), 1.23 (s, 3H), 1.25–1.41 (m, 2H), 1.42 (s, 3H), 1.57–1.73 (m, 2H), 1.78–1.86 (m, 1H), 1.93–2.09 (m, 2H), 2.26–2.40 (m, 2H), 2.44–2.48 (m, 2H), 3.63 (d, *J* = 12.9 Hz, 1H), 3.64 (d, *J* = 12.9 Hz, 1H), 3.84 (ddd, *J =* 12.3, 10.7, 3.1 Hz, 1H), 4.96–5.05 (m, 2H), 5.61 (dddd, *J =* 17.0, 10.1, 8.3, 6.10 Hz, 1H).^13^C NMR (100 MHz, CDCl_3_) δ −2.7, −2.5, 18.6, 22.4, 23.9, 26.1 (3C), 26.8, 31.9, 35.0, 35.1, 35.1, 37.6, 39.1, 40.6, 45.4, 62.6, 66.5, 73.5, 80.4, 118.2, 134.5, 202.8, 208.3. GC/MS EI *m/z* (%) 377 (M^+^˙−57, 32), 359 (3), 347 (5), 331 (2), 285 (6), 267 (8), 239 (8), 223 (9), 195 (10), 182 (19), 135 (21), 81 (46), 75 (100), 41 (45). HRMS calculated for C_25_H_43_O_4_Si (M+H): 435.2925; found: 435.2916.

**X-ray crystallography**

Crystallographic data for structures **23**, **24** and **32** have been deposited with the Cambridge Crystallographic Data Centre (CCDC deposition numbers 670487-9) and may be obtained free of charge via <http://www.ccdc.cam.ac.uk/conts/retrieving.html>, or from the Cambridge Crystallographic Data Centre, 12 Union Road, Cambridge CB2 1EZ, UK; fax: +44 1223 336 033, or e-mail: [deposit@ccdc.cam.ac.uk](mailto:deposit@ccdc.cam.ac.uk).

**References**

1. Perrin, D. D.; Armarego, W. L. F., Eds. *Purification of Laboratory Chemicals,* 3rd ed.; Pergamon: New York, 1988.
2. Imagawa, H.; Kurisaki, T.; Nishizawa, M. *Org. Lett.* **2004,** *6,* 3679–3681. doi:[10.1021/ol048730p](http://dx.doi.org/10.1021/ol048730p)
3. Hellmann, H.; Bader, J.; Birkner, H.; Schumacher, O. *Justus Liebigs Ann. Chem.* **1962,** *659,* 49–63. doi:[10.1002/jlac.19626590107](http://dx.doi.org/10.1002/jlac.19626590107)
