# Supplementary material for: End game strategies towards the total synthesis of vibsanin E, 3-hydroxyvibsanin E, furanovibsanin A, and 3-O-methylfuranovibsanin A
Source: Beilstein J Org Chem. 2008 Oct 8;4:34. doi: 10.3762/bjoc.4.34 (PMC2577281; doi:10.3762/bjoc.4.34)
Supplement: File 2 — NMR spectra [file Beilstein_J_Org_Chem-04-34-s002.pdf]

# End game strategies towards the total synthesis of vibsanin E, 3-hydroxyvibsanin E, furanovibsanin A, and 3-*O*-methylfuranovibsanin A

Brett D. Schwartz, Craig M. Williams* and Paul V. Bernhardt

Address: School of Molecular and Microbial Sciences, University of Queensland, Brisbane, 4072, Queensland, Australia

Email: Craig M. Williams* - c.williams3@uq.edu.au

* Corresponding author

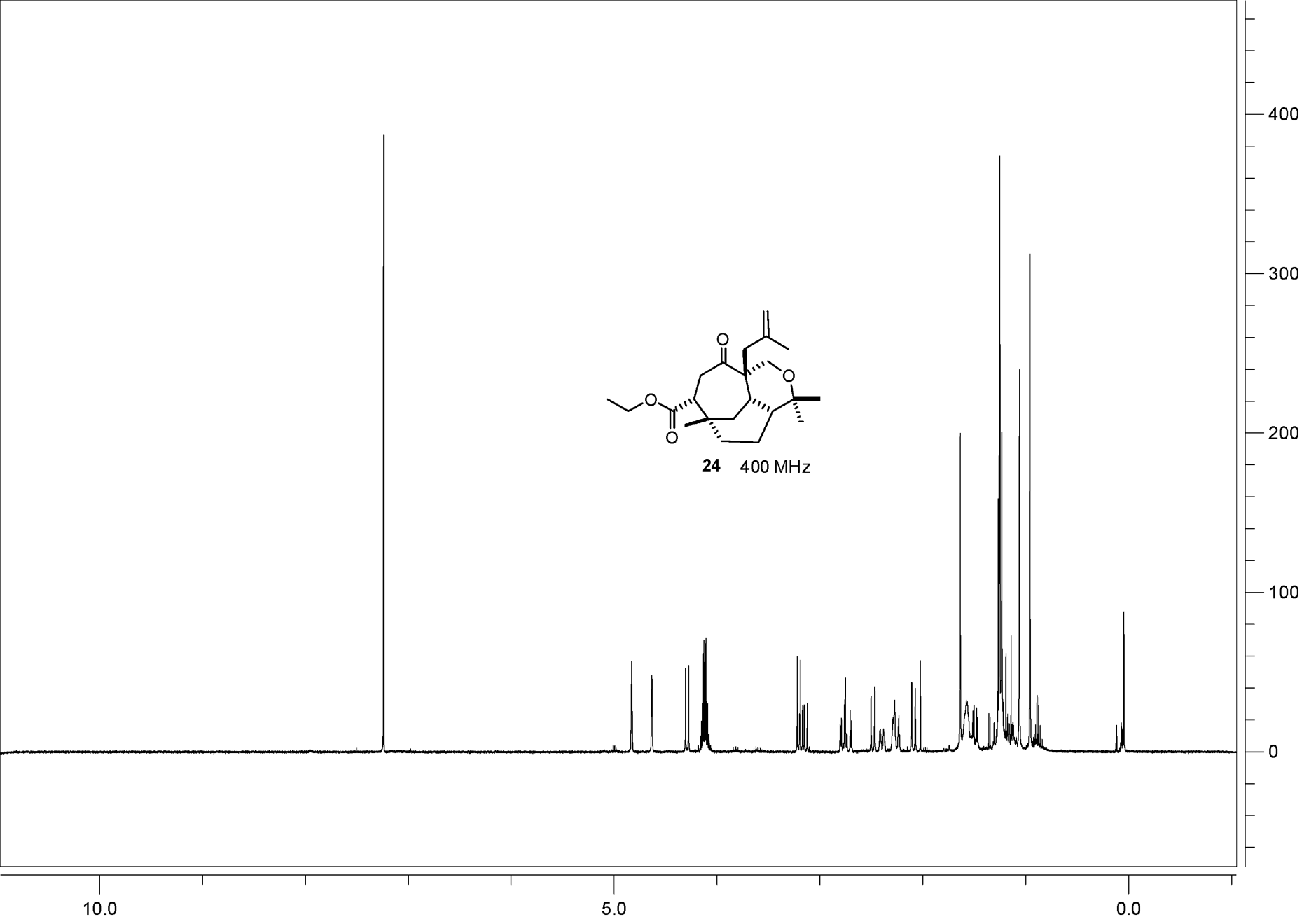
24 400 MHz
400
300
200
100
0
10.0
5.0
0.0
ppm (f1)

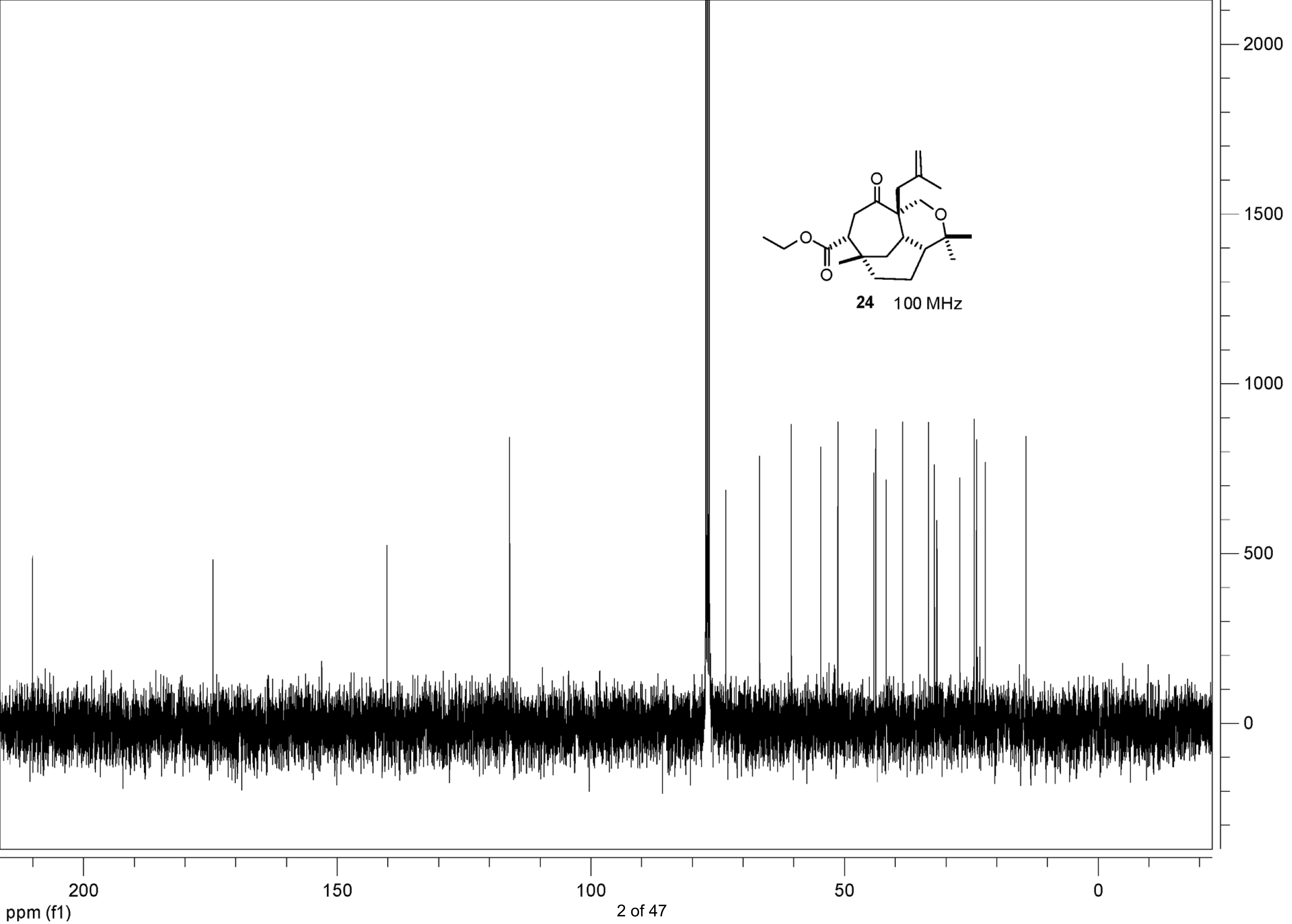
24 100 MHz
2000
1500
1000
500
0
200
150
100
50
0
ppm (f1)

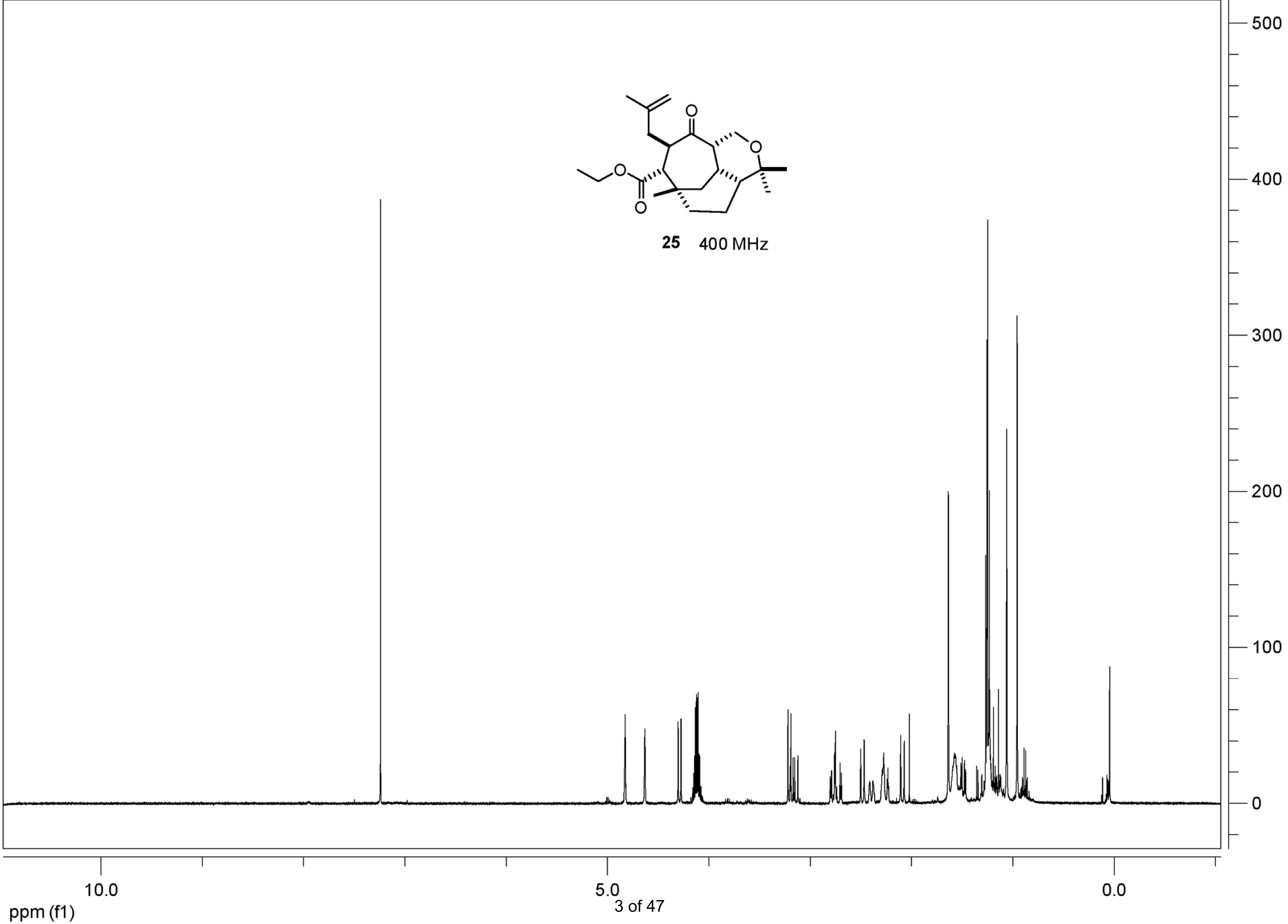
25 400 MHz
500
400
300
200
100
0
10.0
5.0
0.0
ppm (f1)

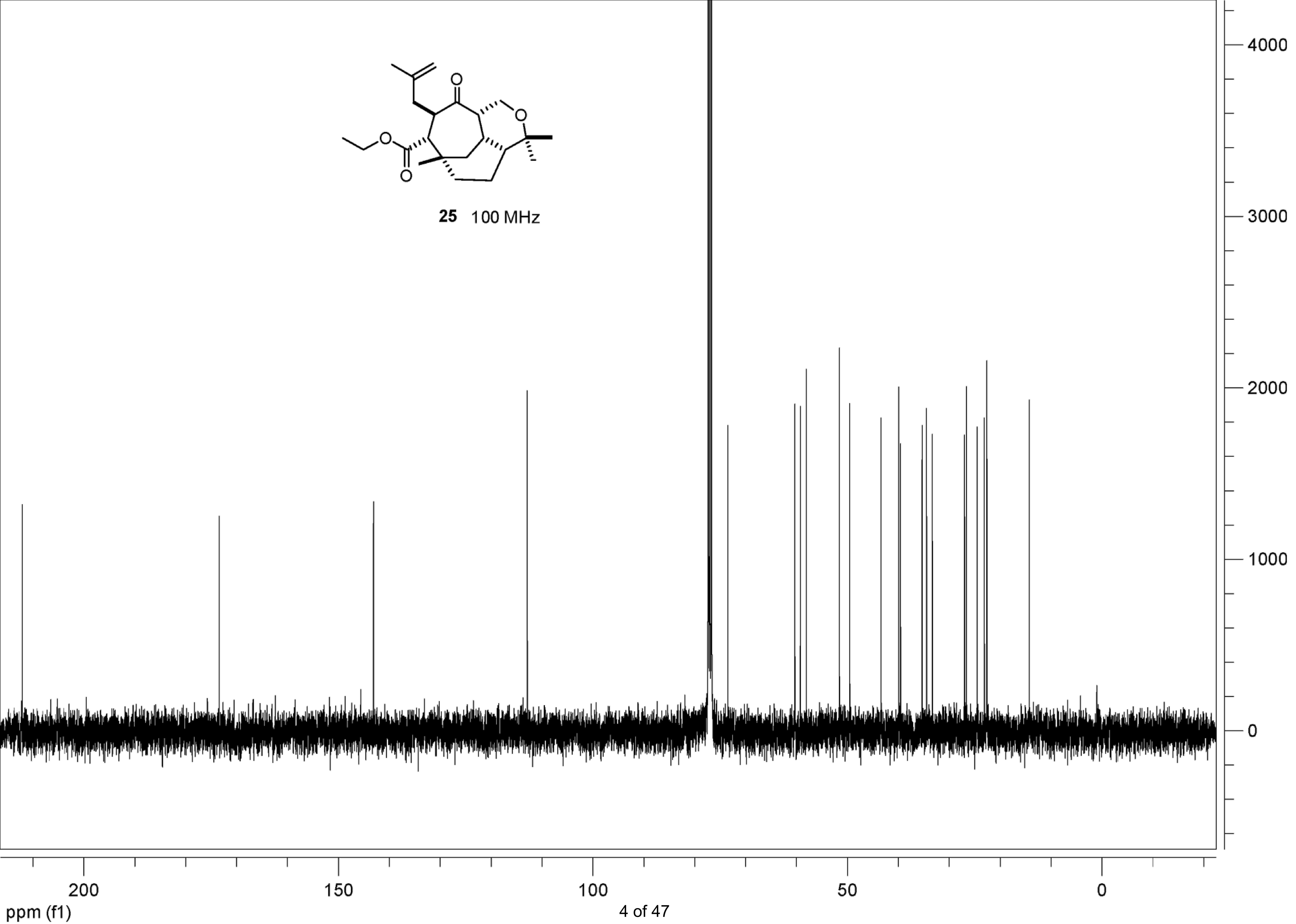
25 100 MHz
4000
3000
2000
1000
0
200
150
100
50
0
ppm (f1)

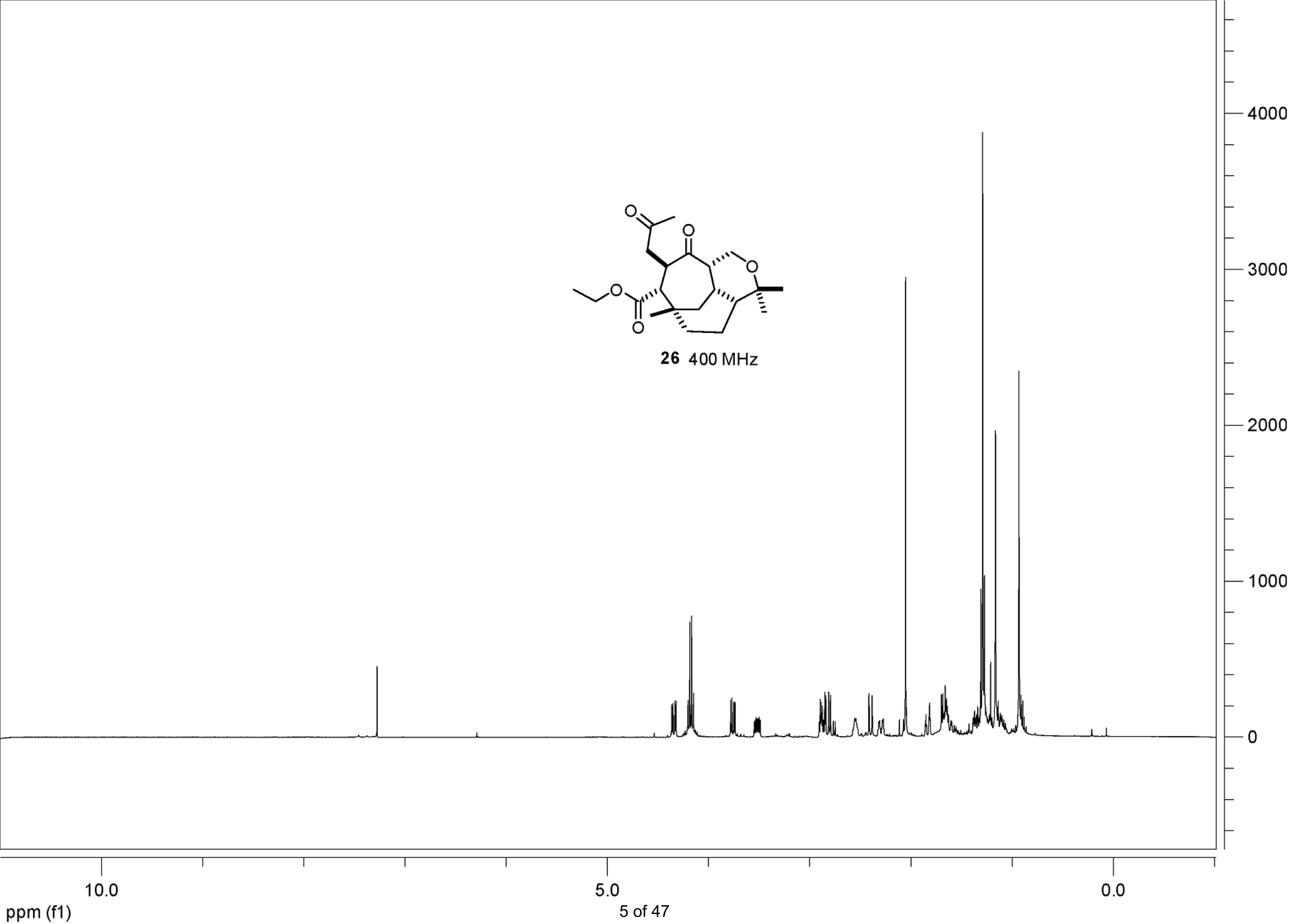

26 400 MHz
4000
3000
2000
1000
0
10.0
5.0
0.0
ppm (f1)

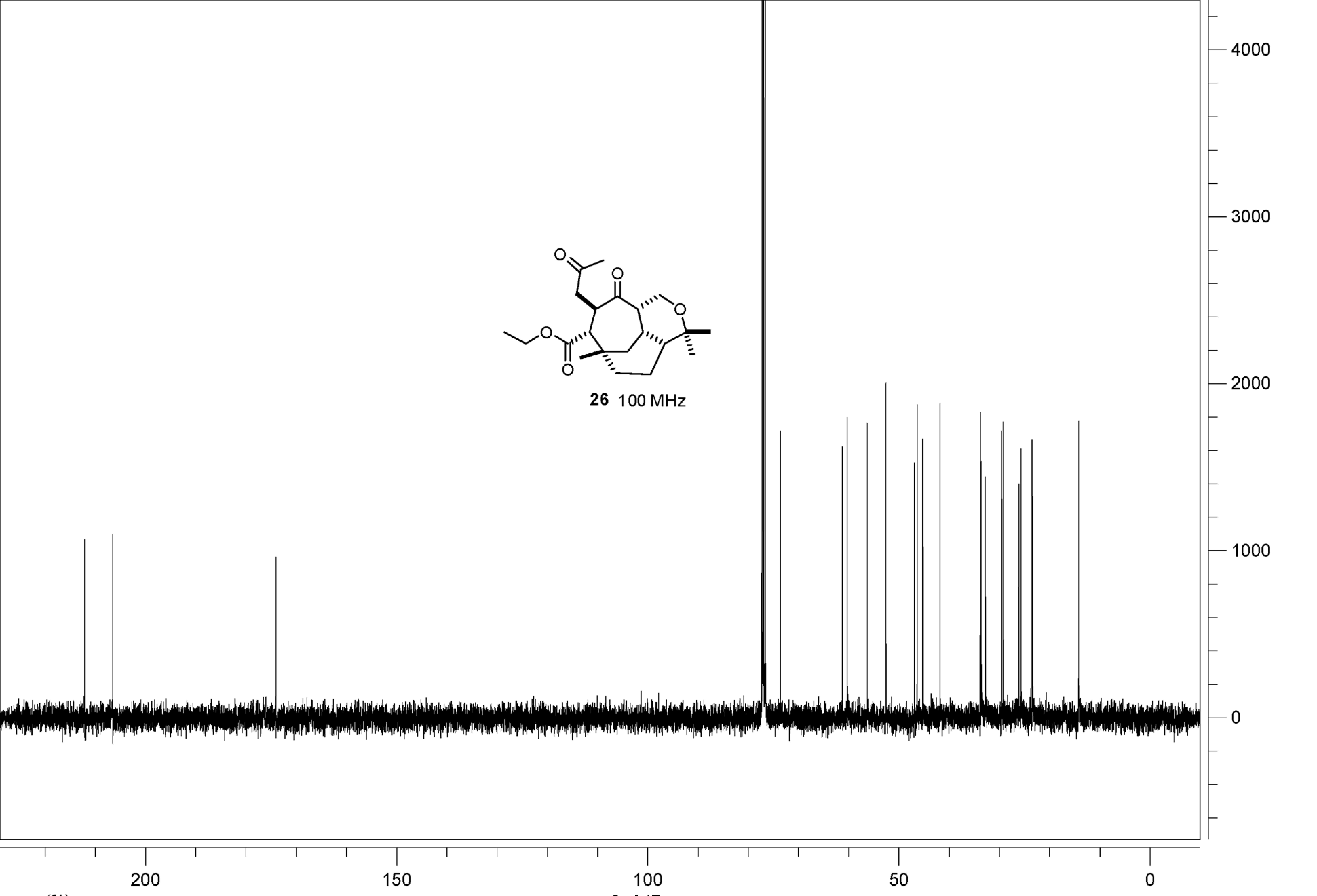
26 100 MHz
4000
3000
2000
1000
0
200
150
100
50
0
ppm (f1)

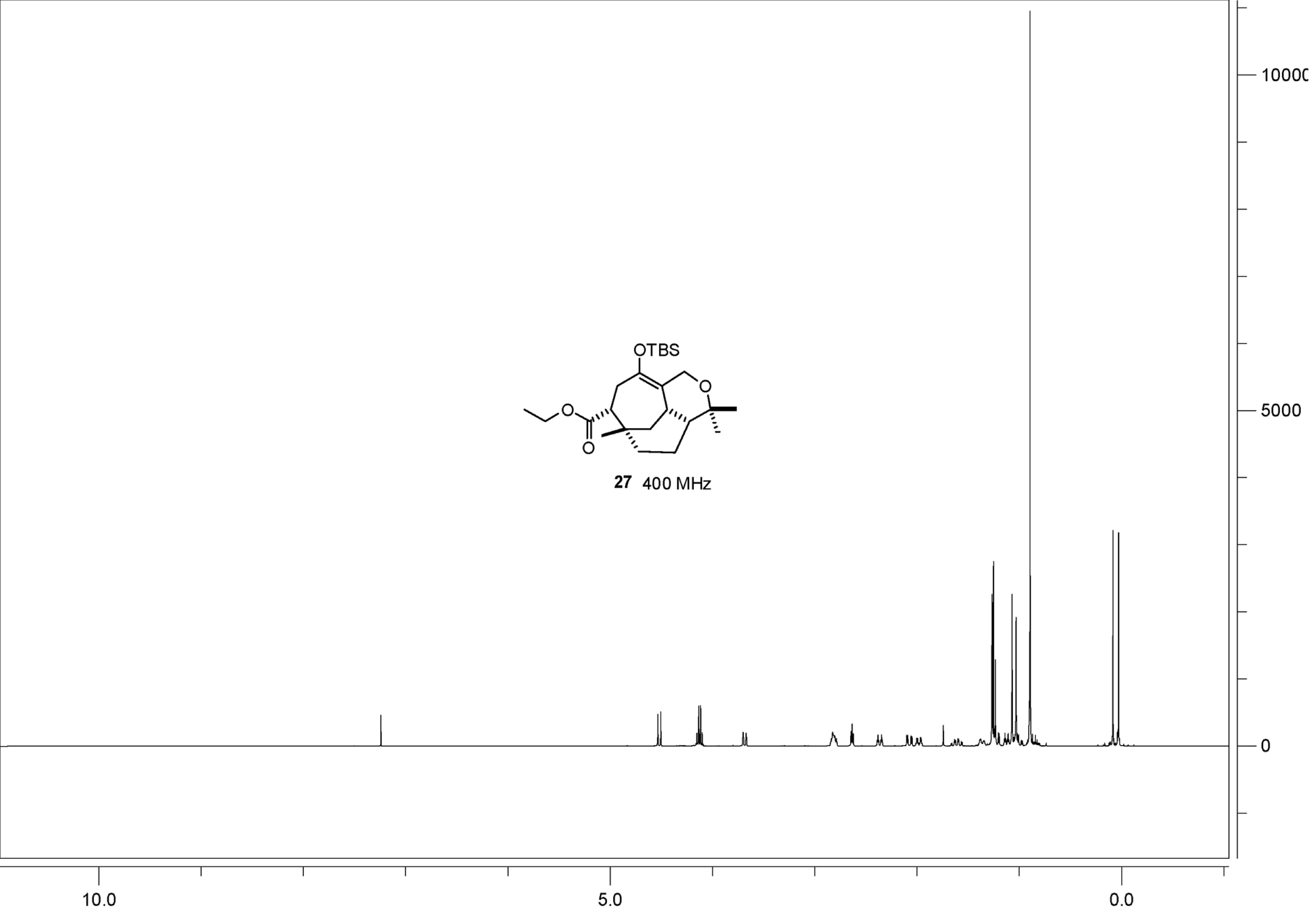
OTBS
O
O
O
27 400 MHz
10000
5000
0
10.0
5.0
0.0
ppm (f1)

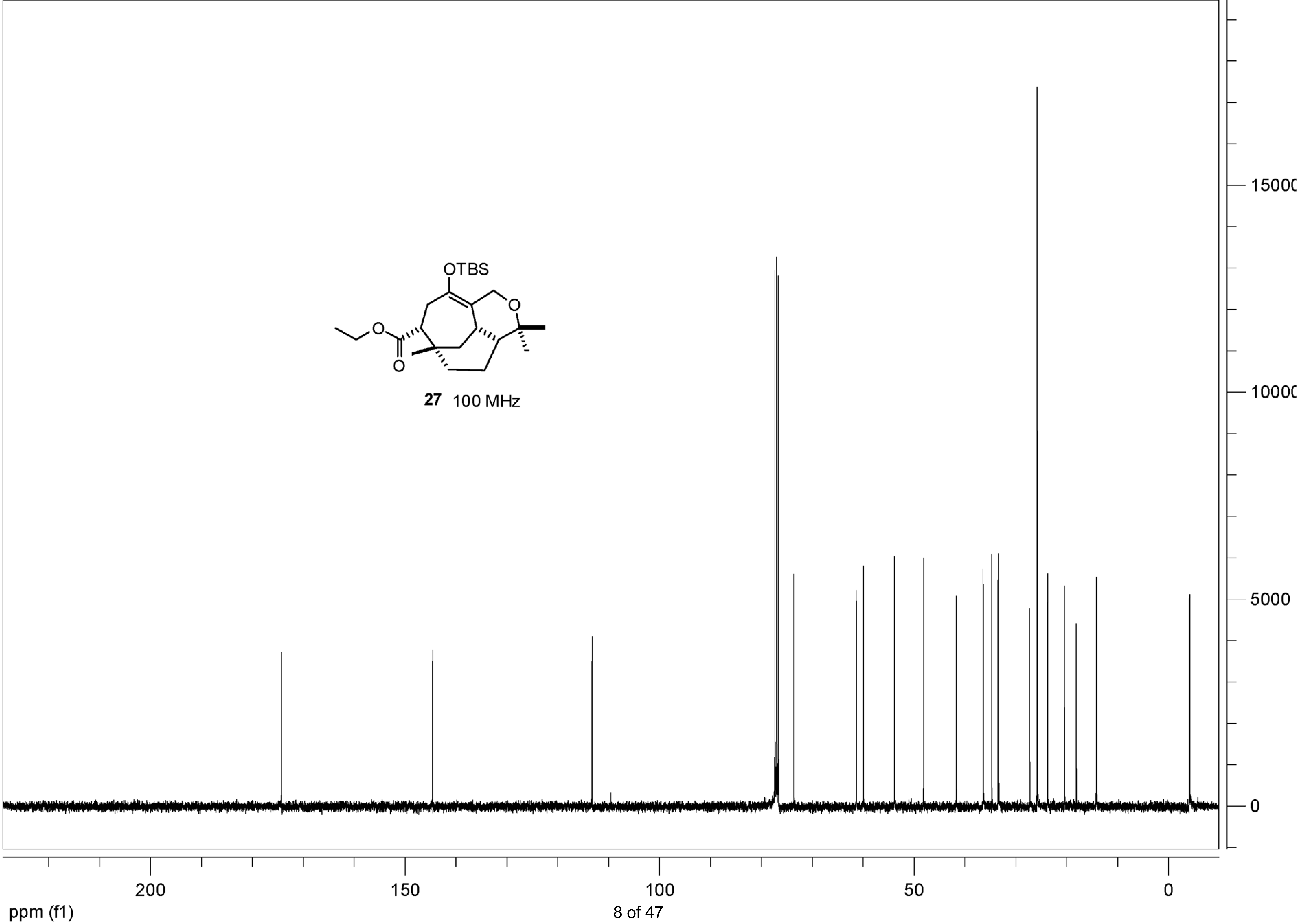
OTBS
O
O
O
27 100 MHz
15000
10000
5000
0
200
150
100
50
0
ppm (f1)

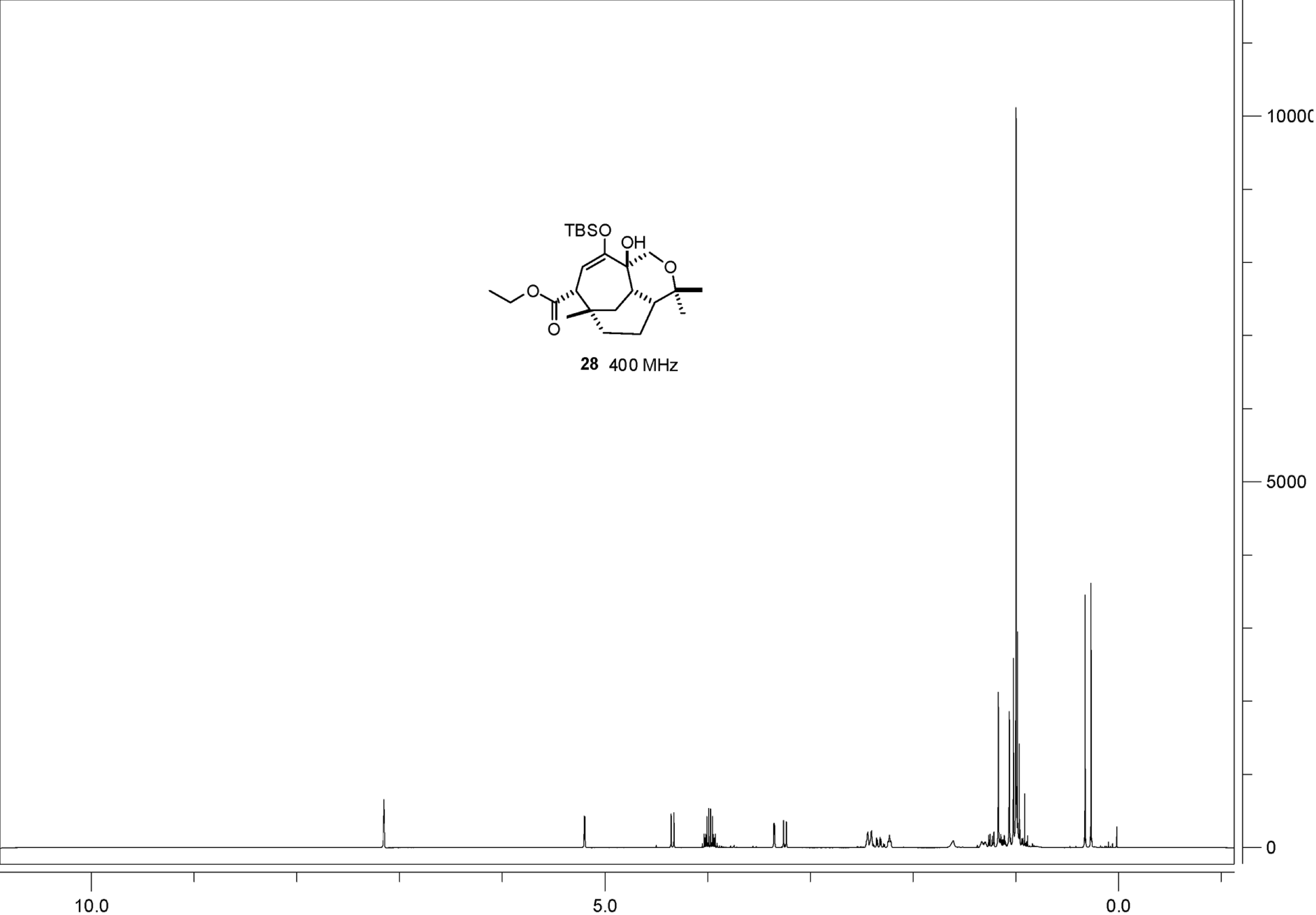
TBSO
OH
O
O
O
28 400 MHz
10000
5000
0
10.0
5.0
0.0
ppm (f1)

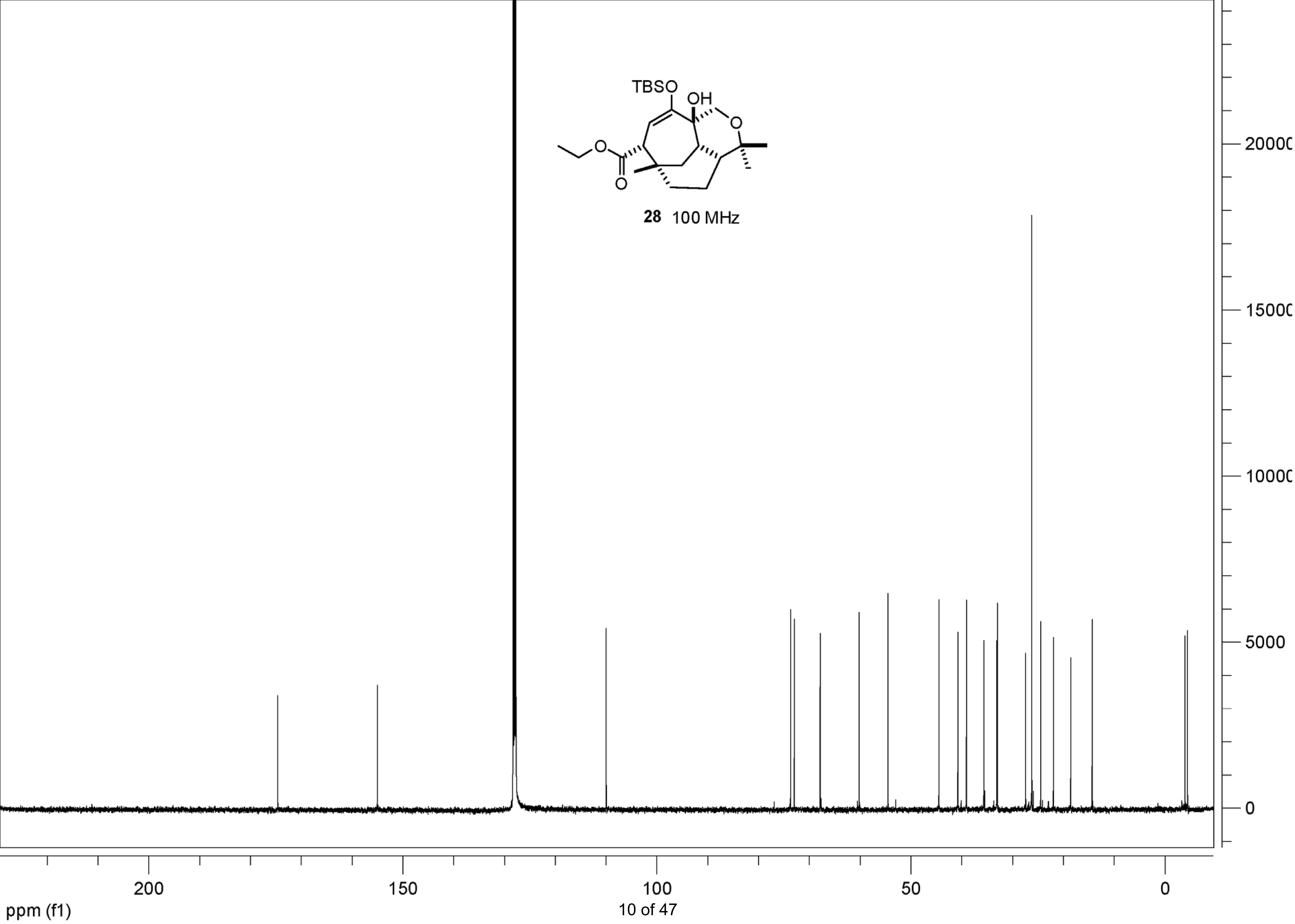
TBSO
OH
O
O
O
28 100 MHz
20000
15000
10000
5000
0
200
150
100
50
0
ppm (f1)

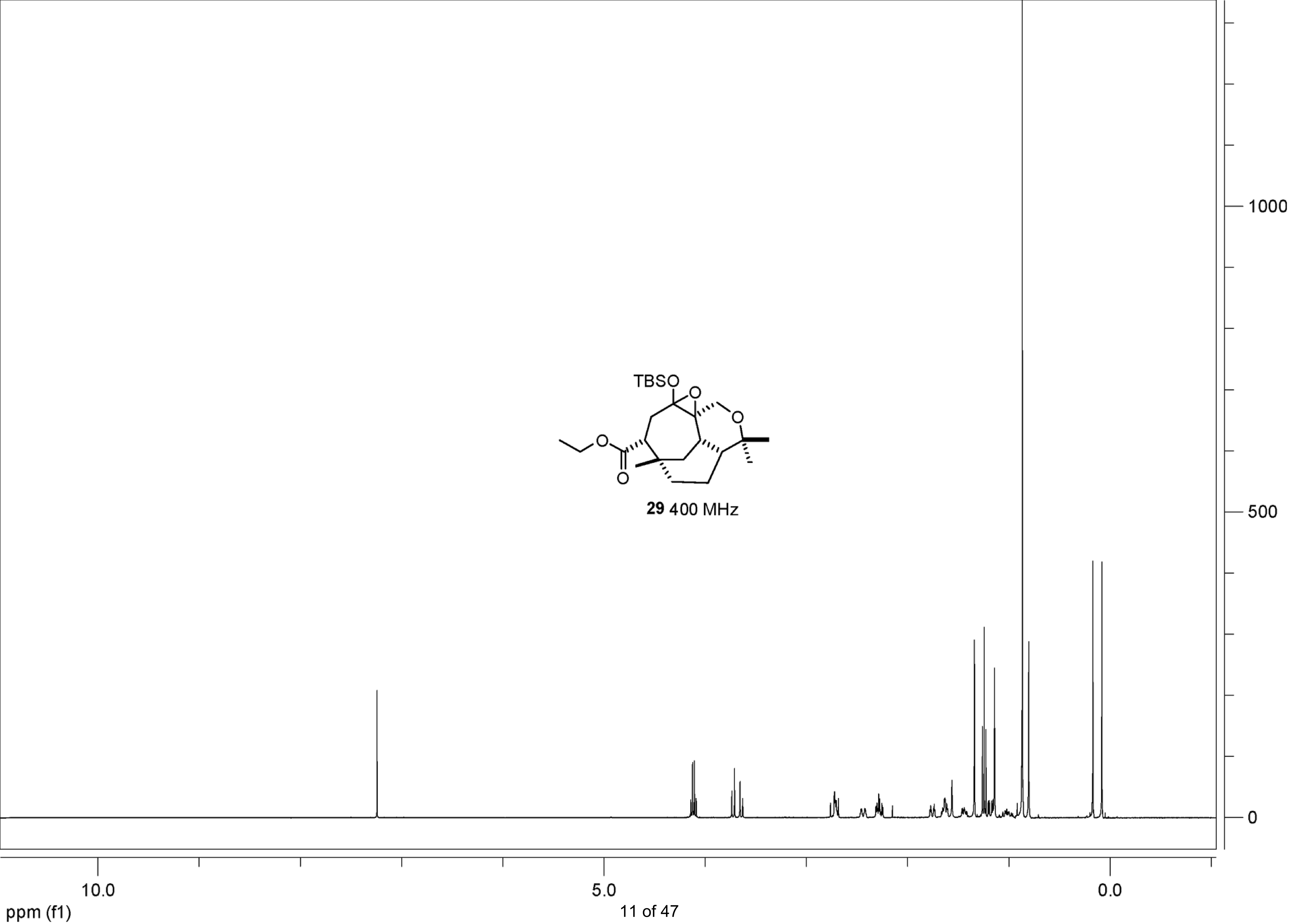
TBSO
O
O
O
O
29 400 MHz
1000
500
0
10.0
5.0
0.0
ppm (f1)

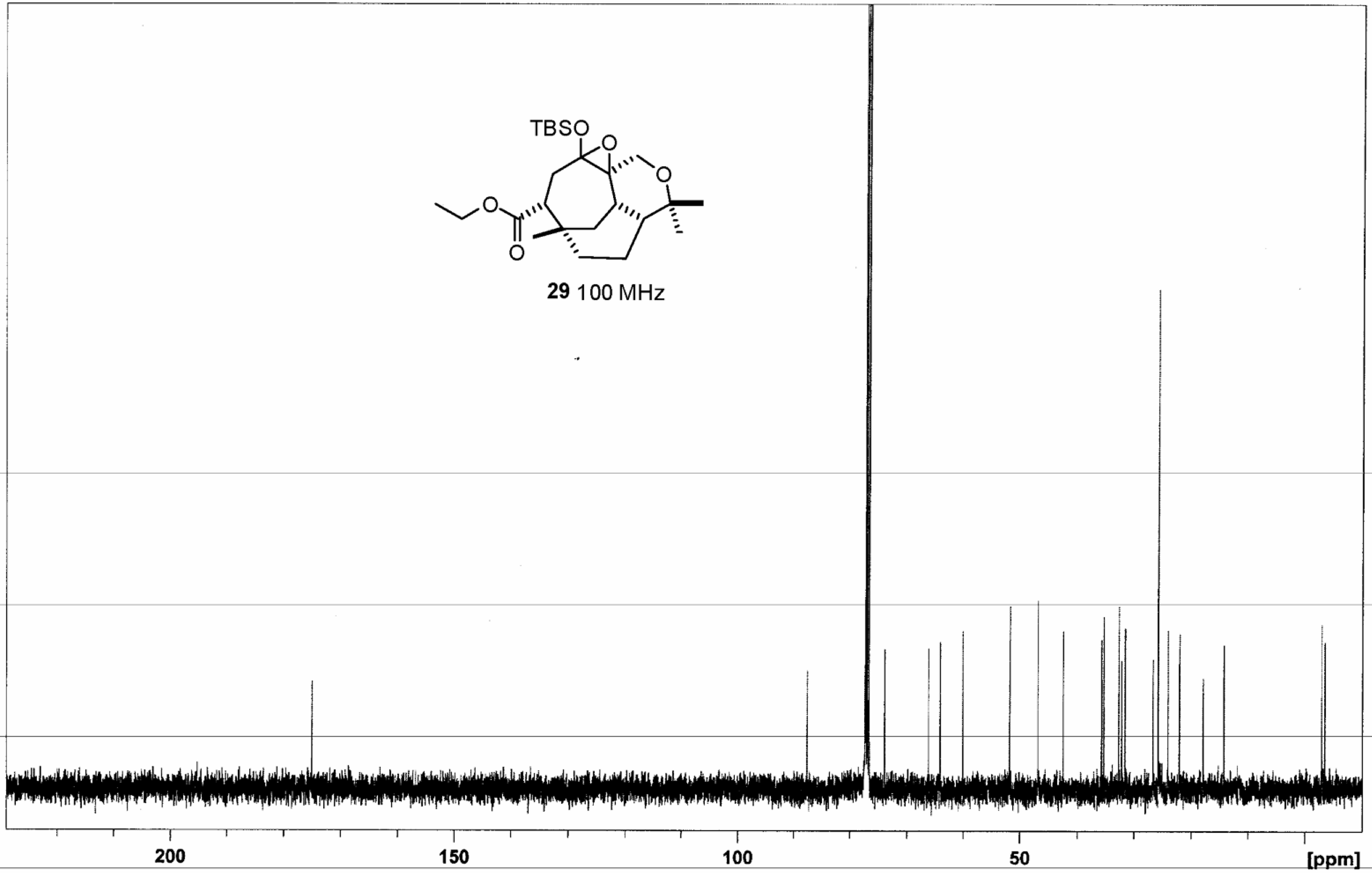
TBSO
O
O
O
O
29 100 MHz
200
150
100
50
[ppm]

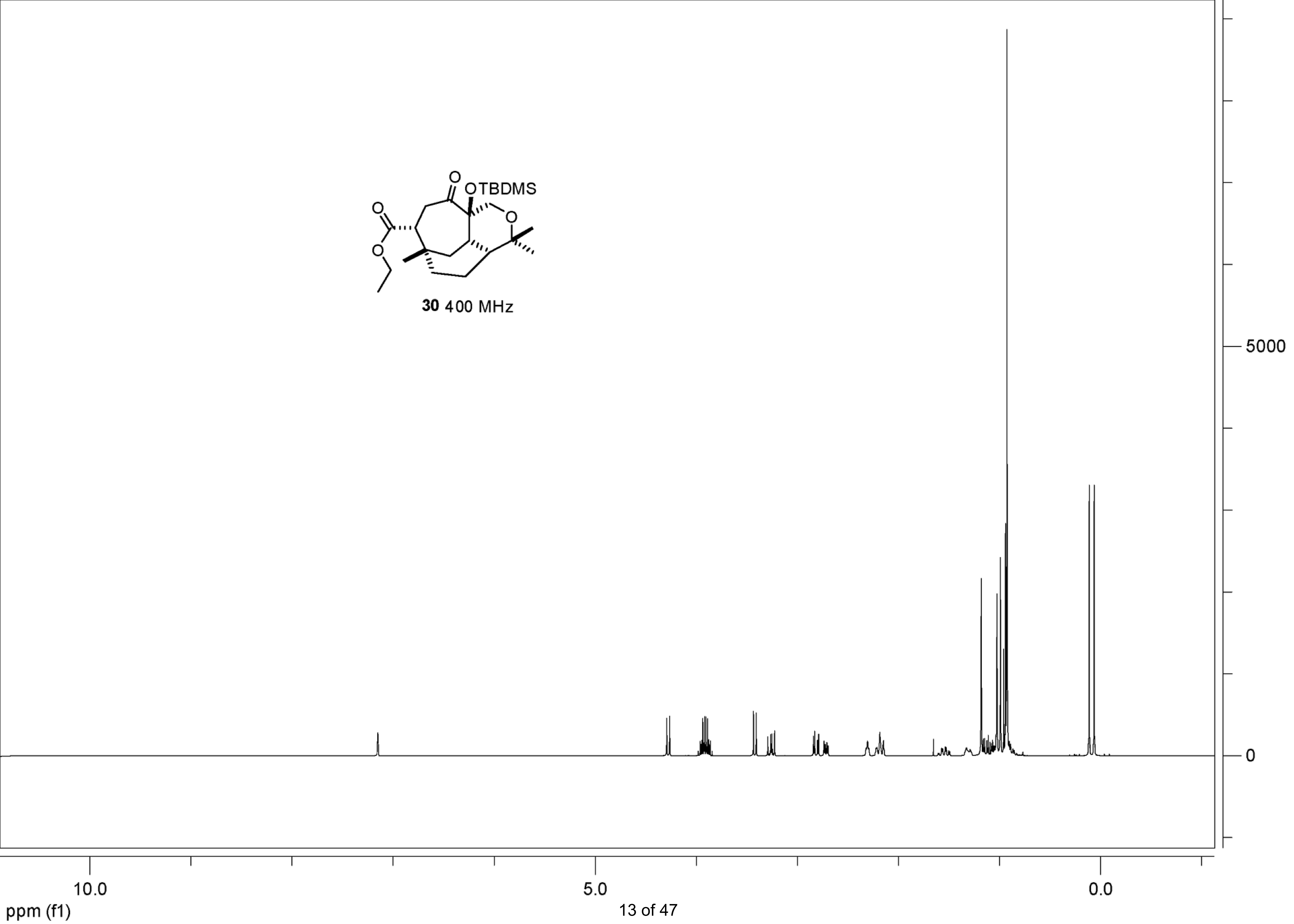
OTBDMS
O
O
O
O
30 400 MHz
5000
0
10.0
5.0
0.0
ppm (f1)

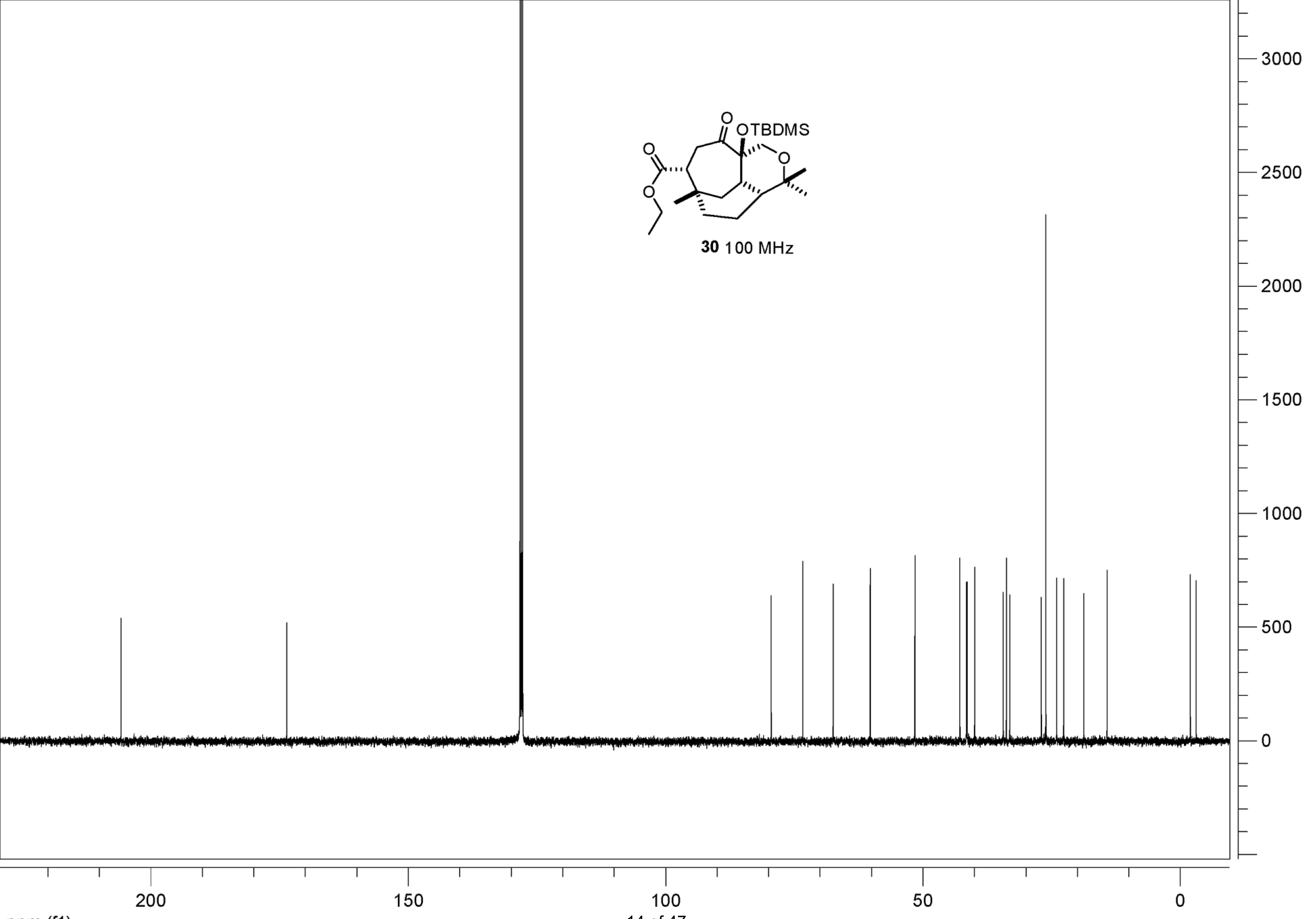
OTBDMS
O
O
O
30 100 MHz
3000
2500
2000
1500
1000
500
0
200
150
100
50
0
ppm (f1)

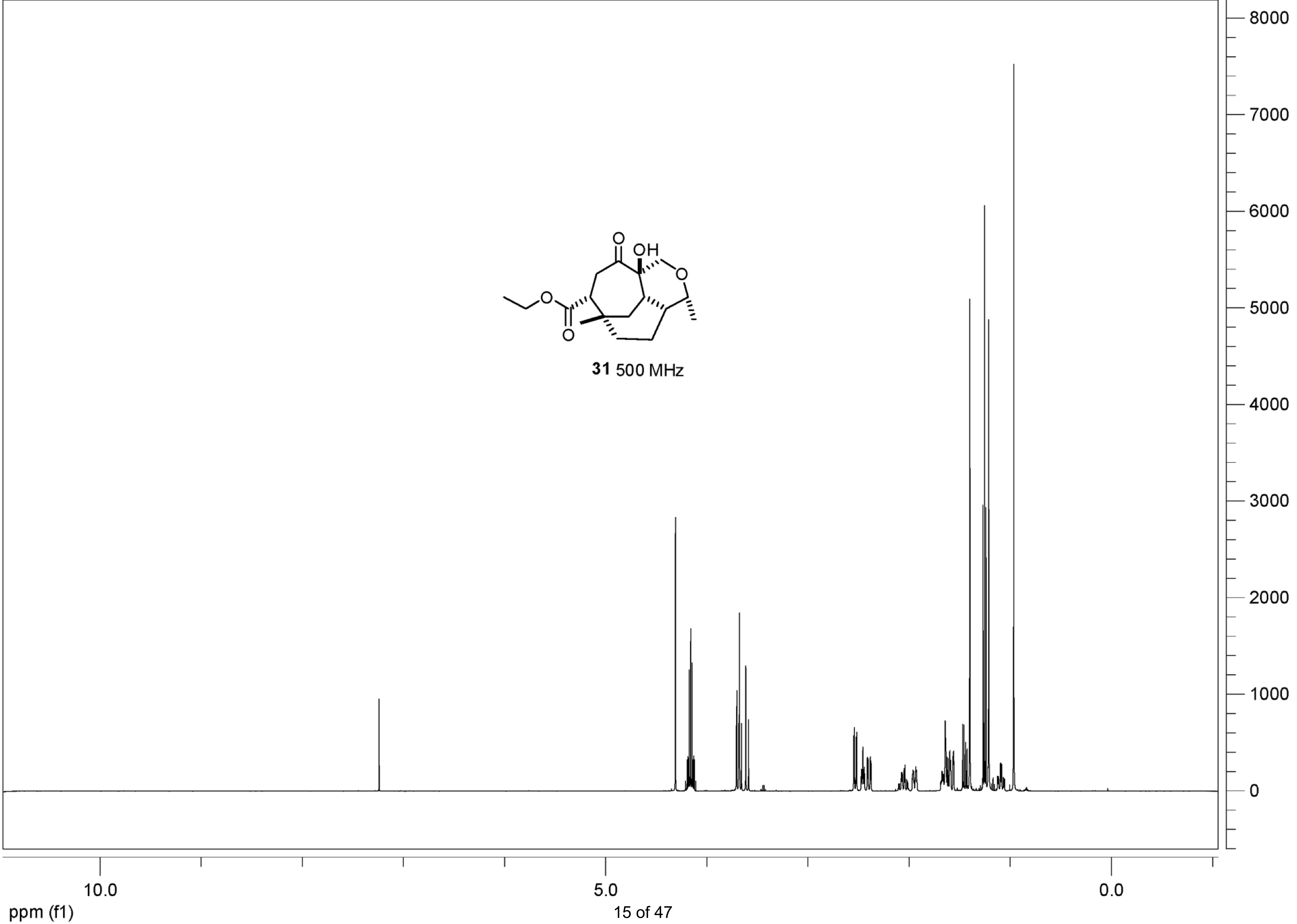

31 500 MHz
OH
O
8000
7000
6000
5000
4000
3000
2000
1000
0
10.0
5.0
0.0
ppm (f1)

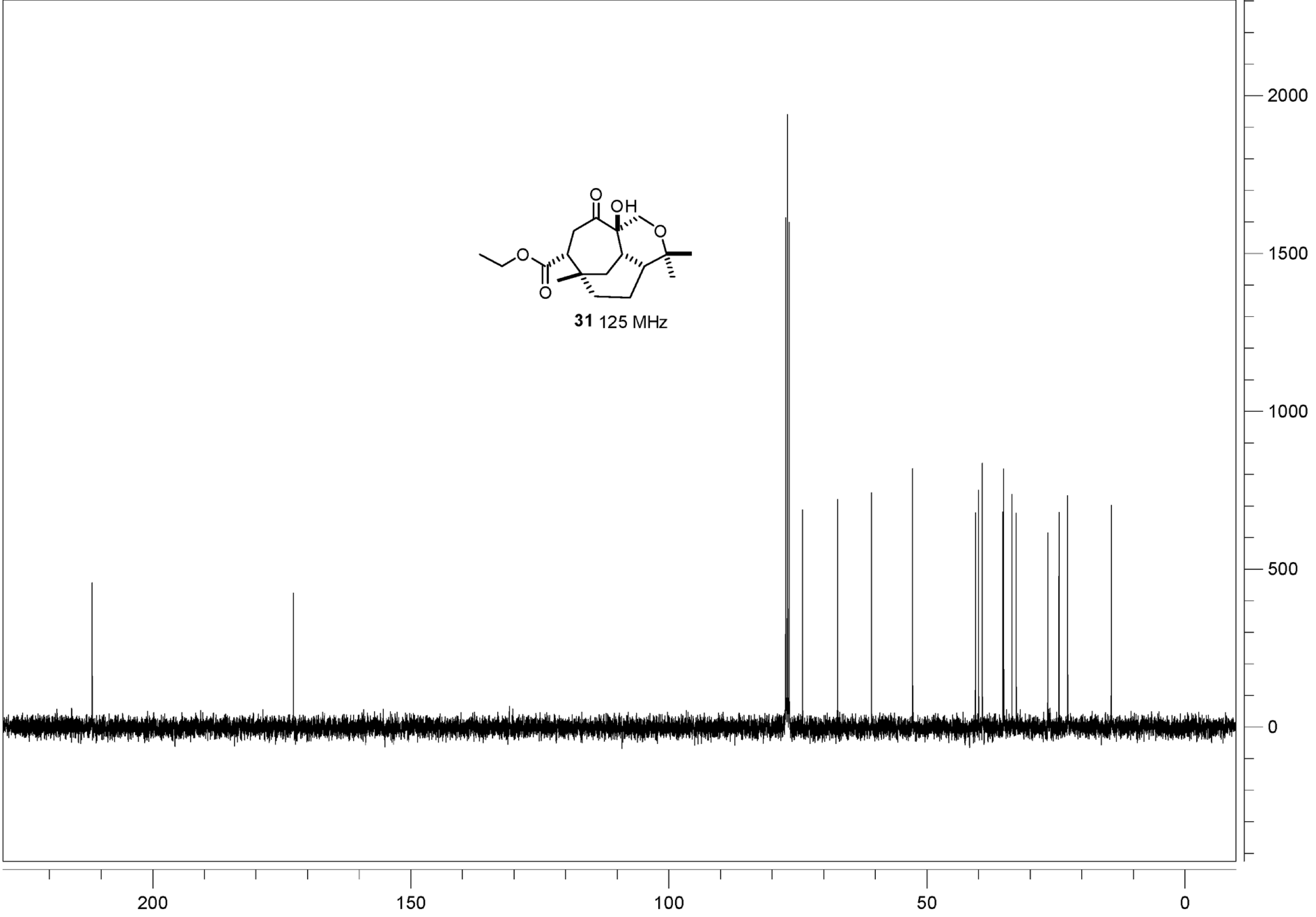
O
OH
O
O
O
31 125 MHz
2000
1500
1000
500
0
200
150
100
50
0
ppm (f1)

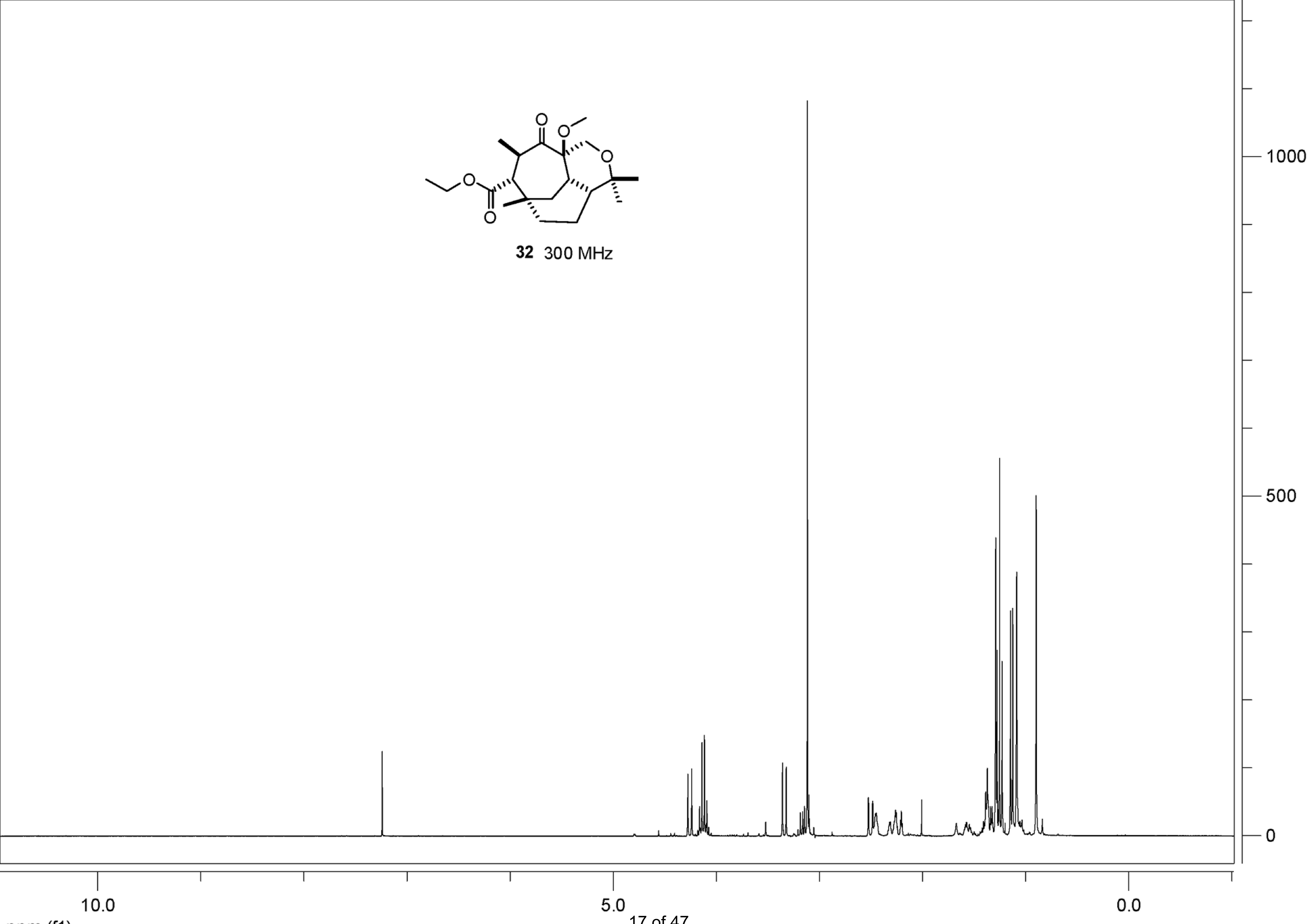
O
O
O
O
O
32 300 MHz
1000
500
0
10.0
5.0
0.0
ppm (f1)

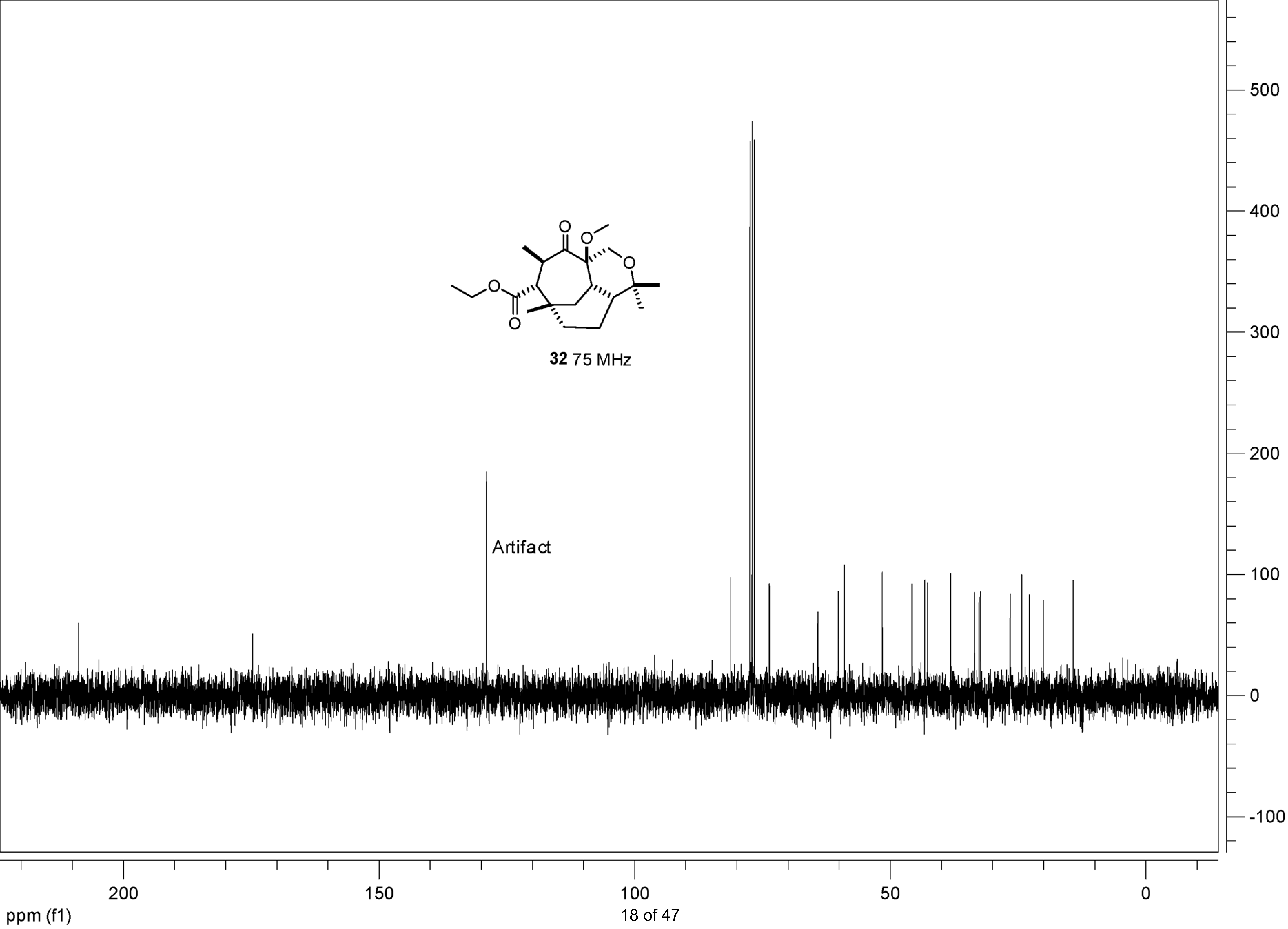

O
O
O
O
O
32 75 MHz
Artifact
500
400
300
200
100
0
-100
200
150
100
50
0
ppm (f1)

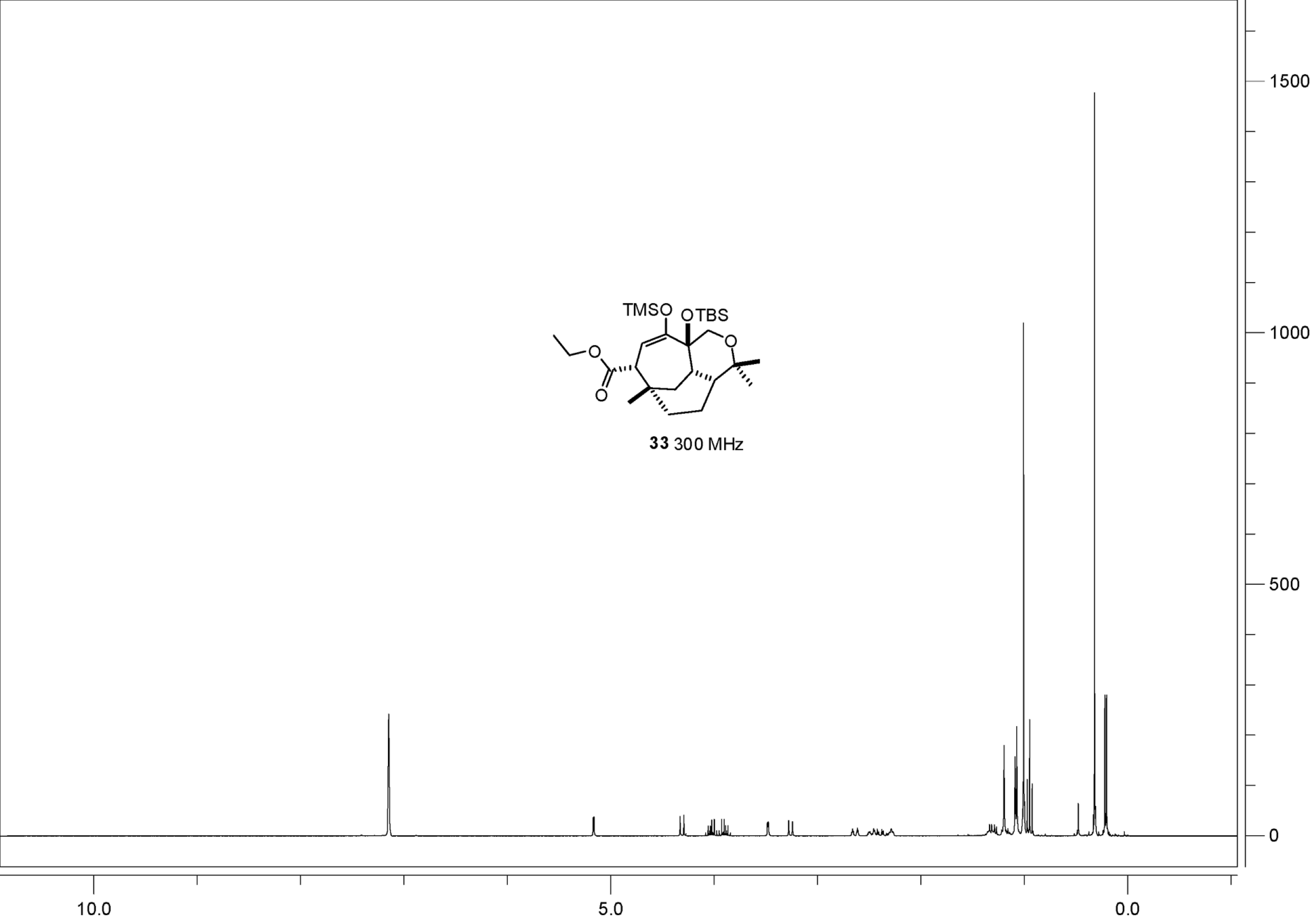
TMSO
OTBS
O
O
O
33 300 MHz
1500
1000
500
0
10.0
5.0
0.0
ppm (f1)

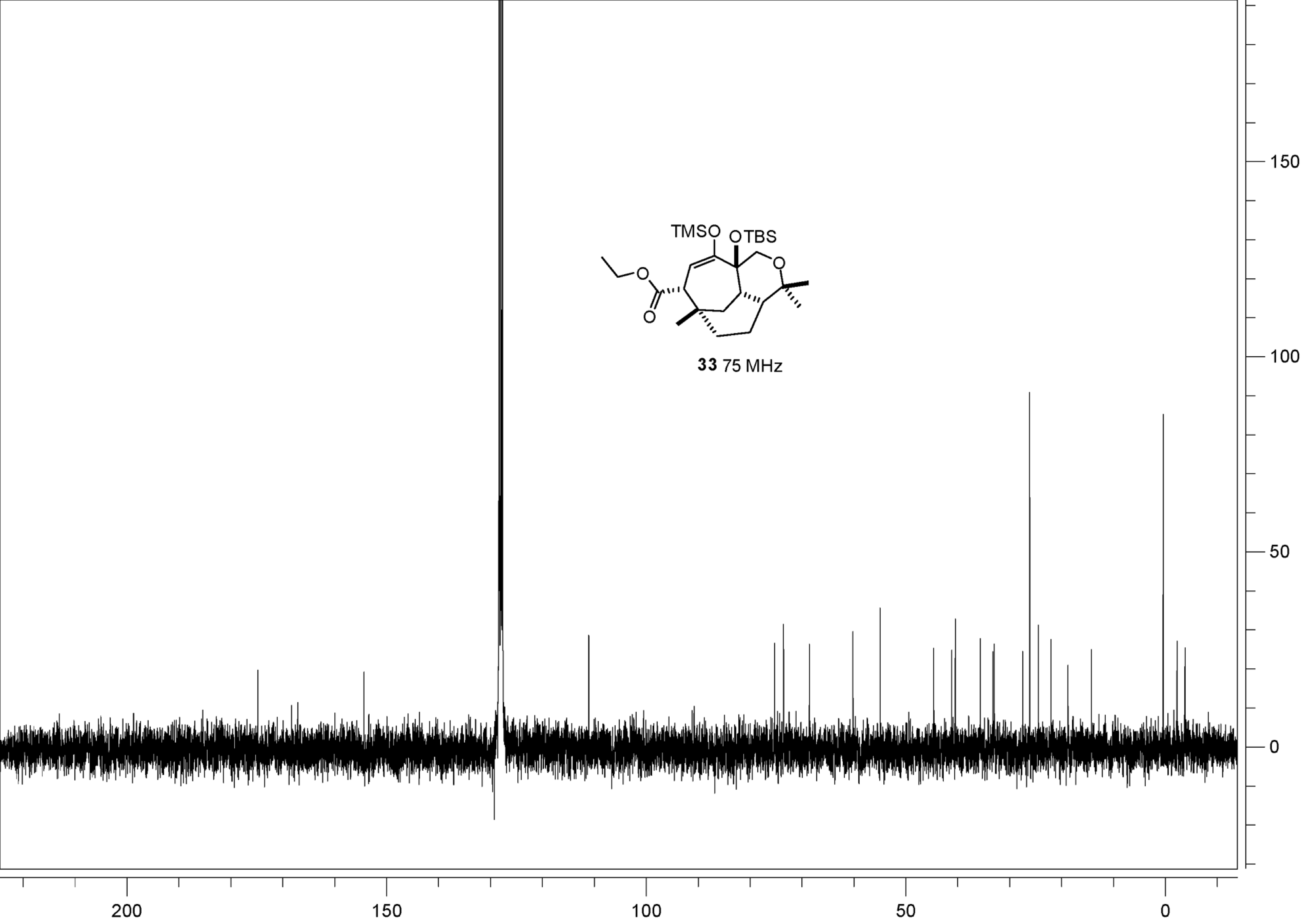
TMSO
OTBS
O
O
O
33 75 MHz
150
100
50
0
200
150
100
50
0
ppm (f1)

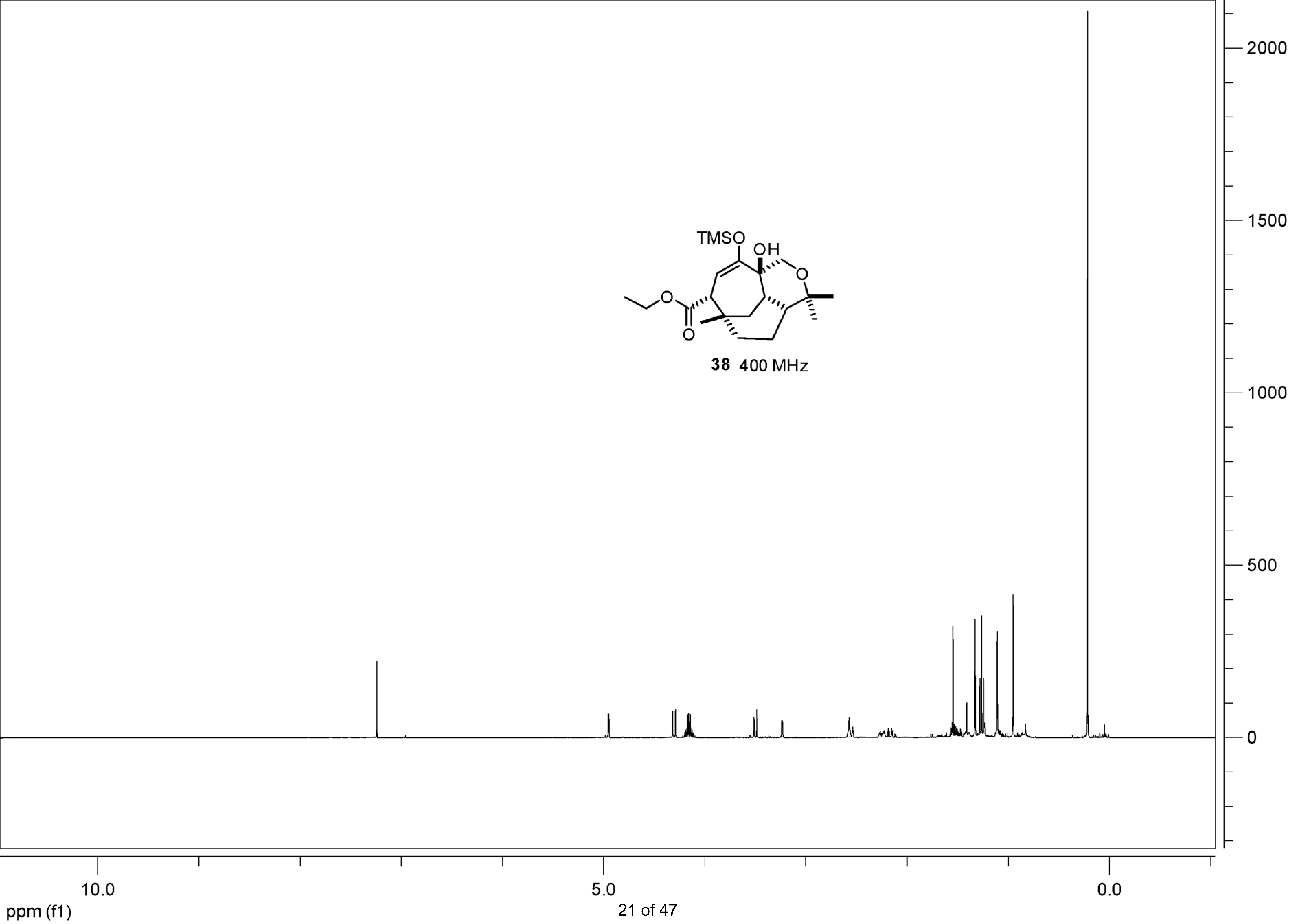
TMSO
OH
O
O
O
O
38 400 MHz
2000
1500
1000
500
0
10.0
5.0
0.0
ppm (f1)

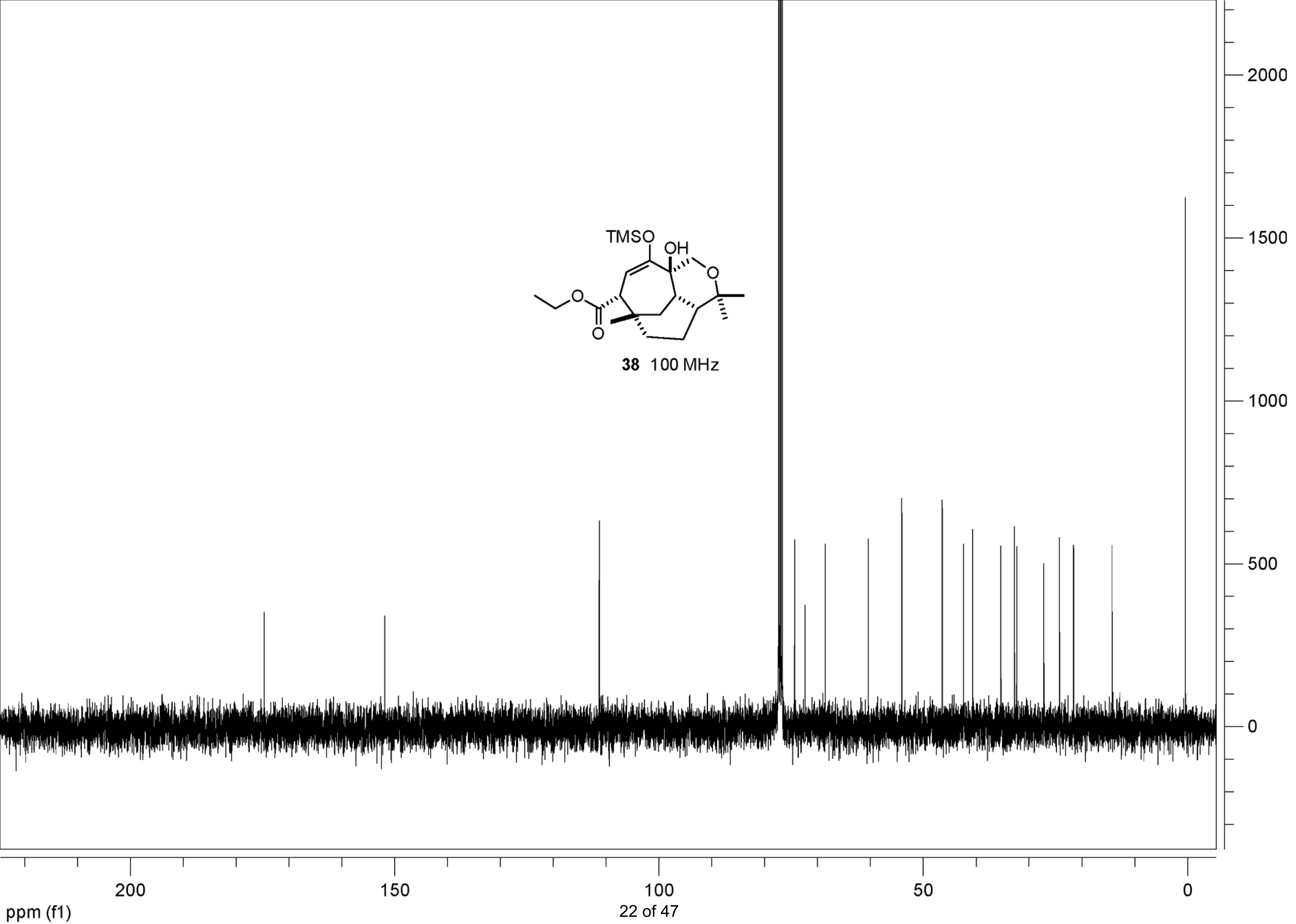
TMSO
OH
O
O
O
38 100 MHz
2000
1500
1000
500
0
200
150
100
50
0
ppm (f1)

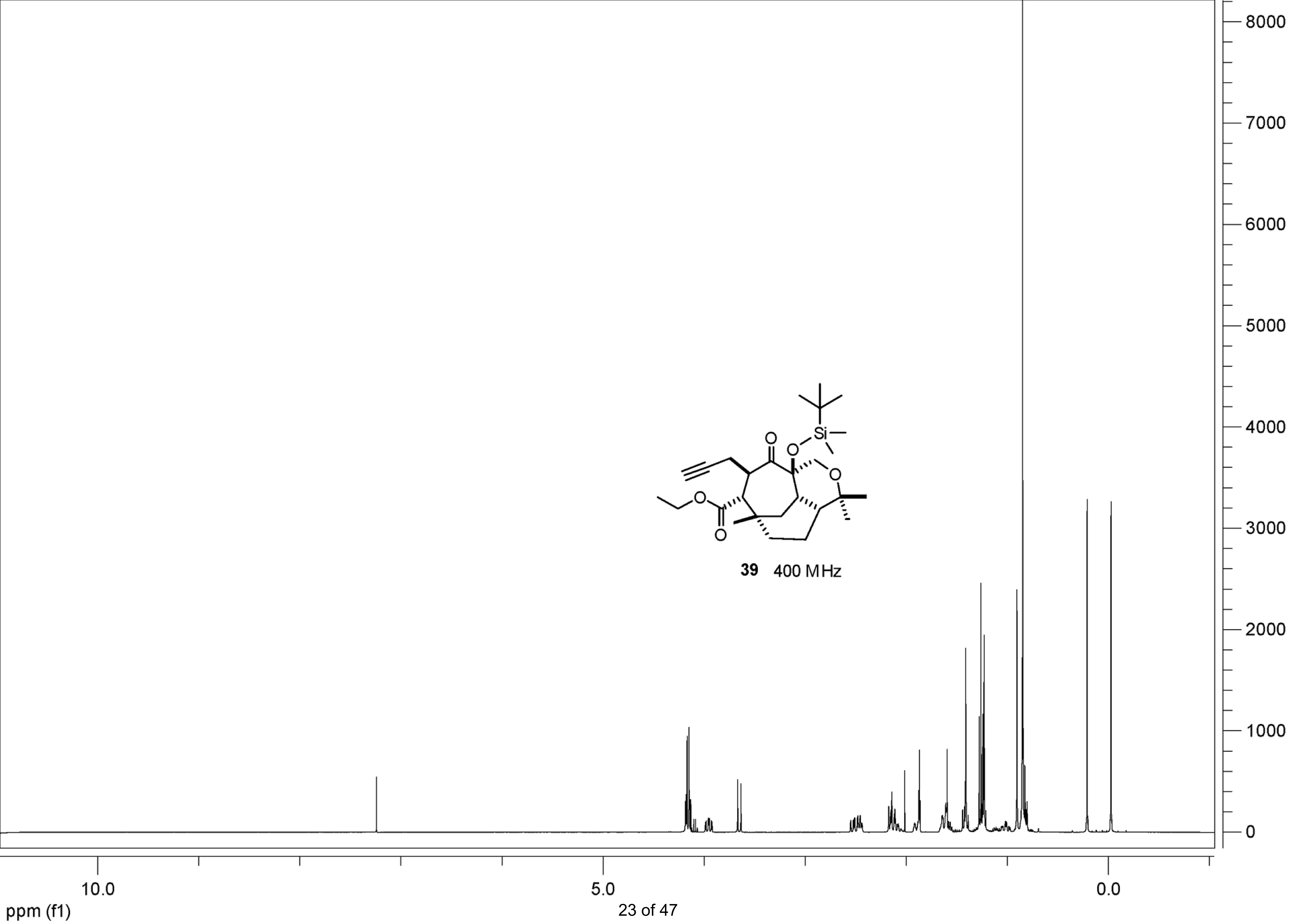
39 400 MHz
8000
7000
6000
5000
4000
3000
2000
1000
0
10.0
5.0
0.0
ppm (f1)

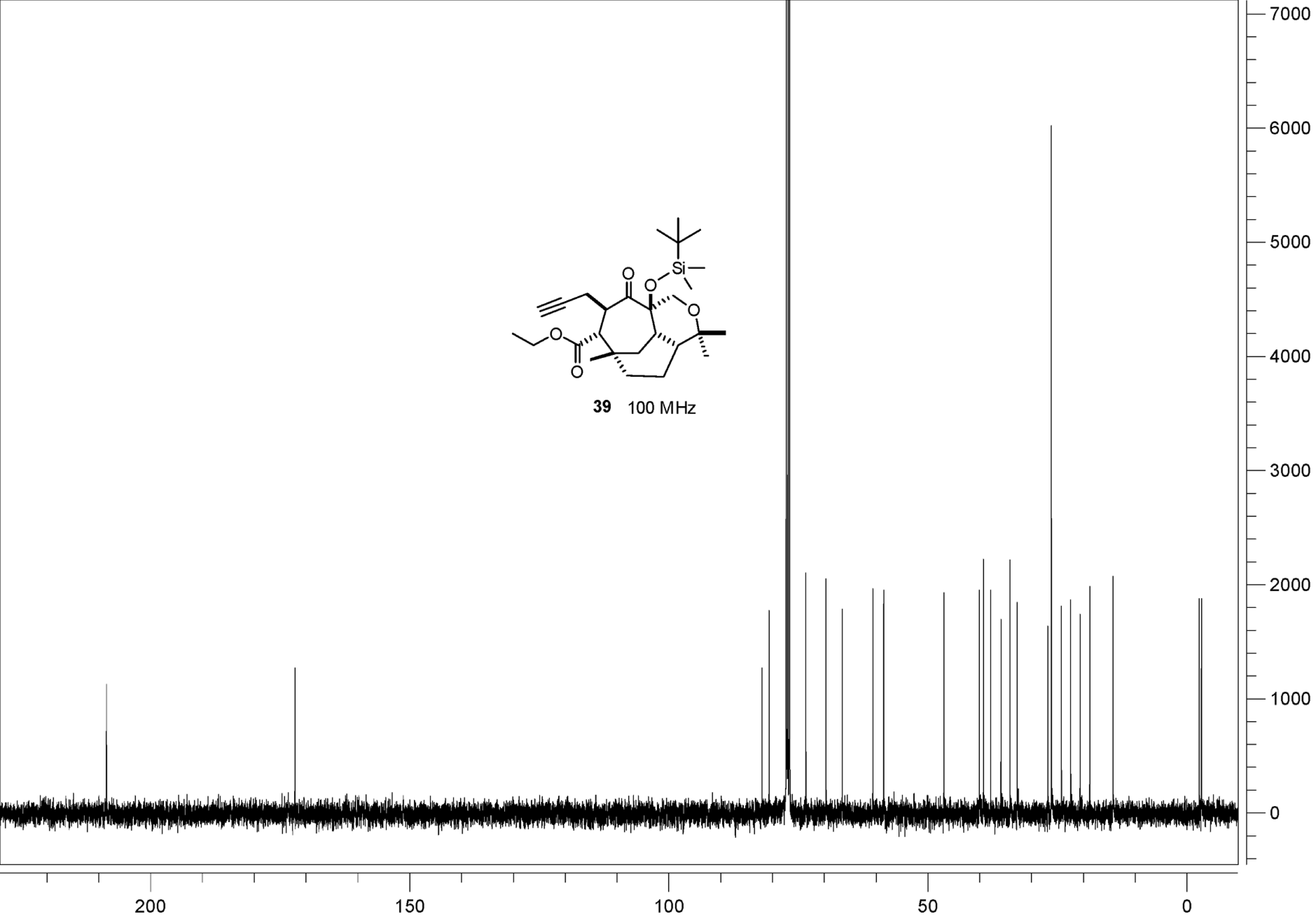

39 100 MHz
O
Si
7000
6000
5000
4000
3000
2000
1000
0
200
150
100
50
0
ppm (f1)

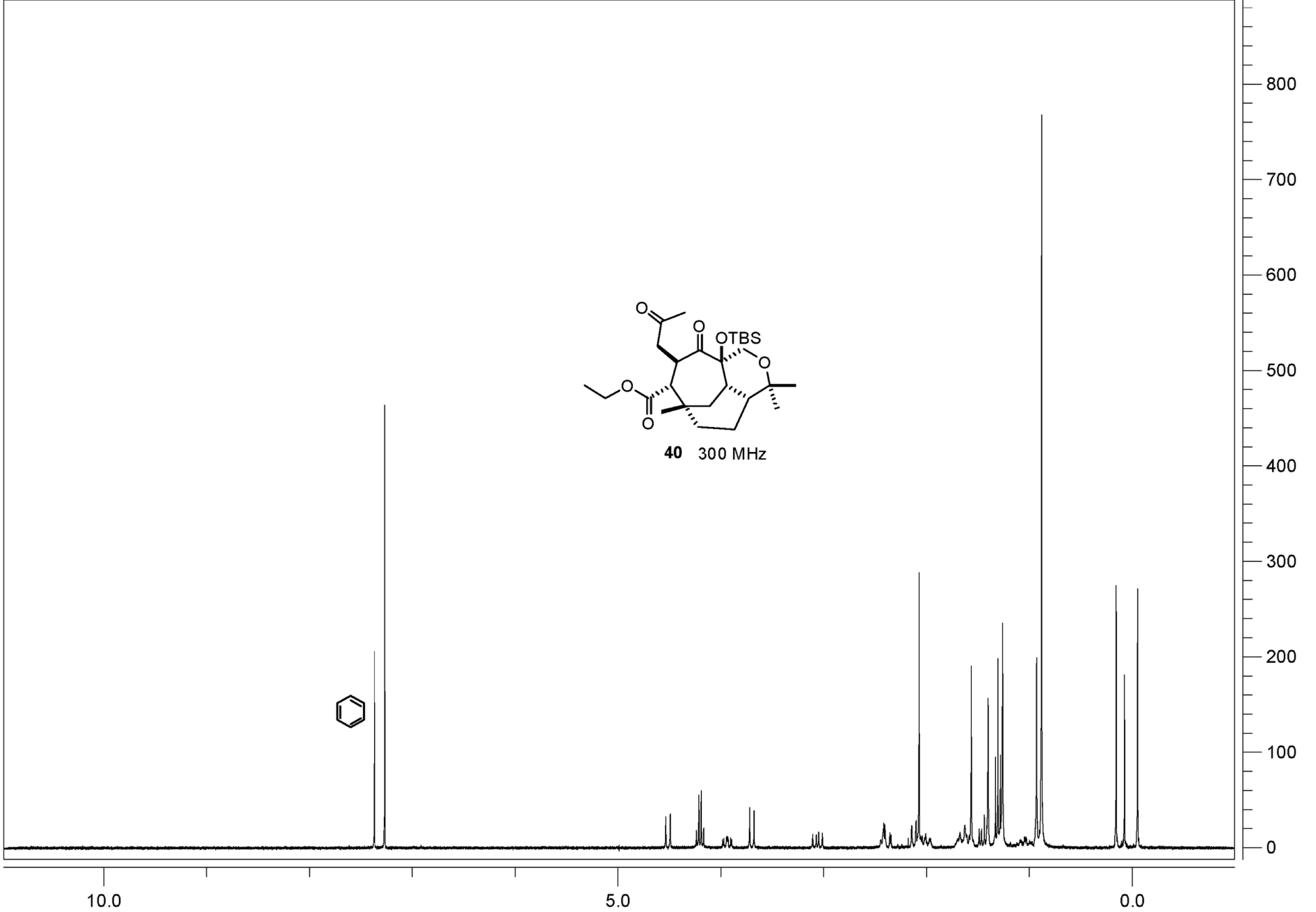
O
O
OTBS
O
O
O
40 300 MHz
800
700
600
500
400
300
200
100
0
10.0
5.0
0.0
ppm (f1)

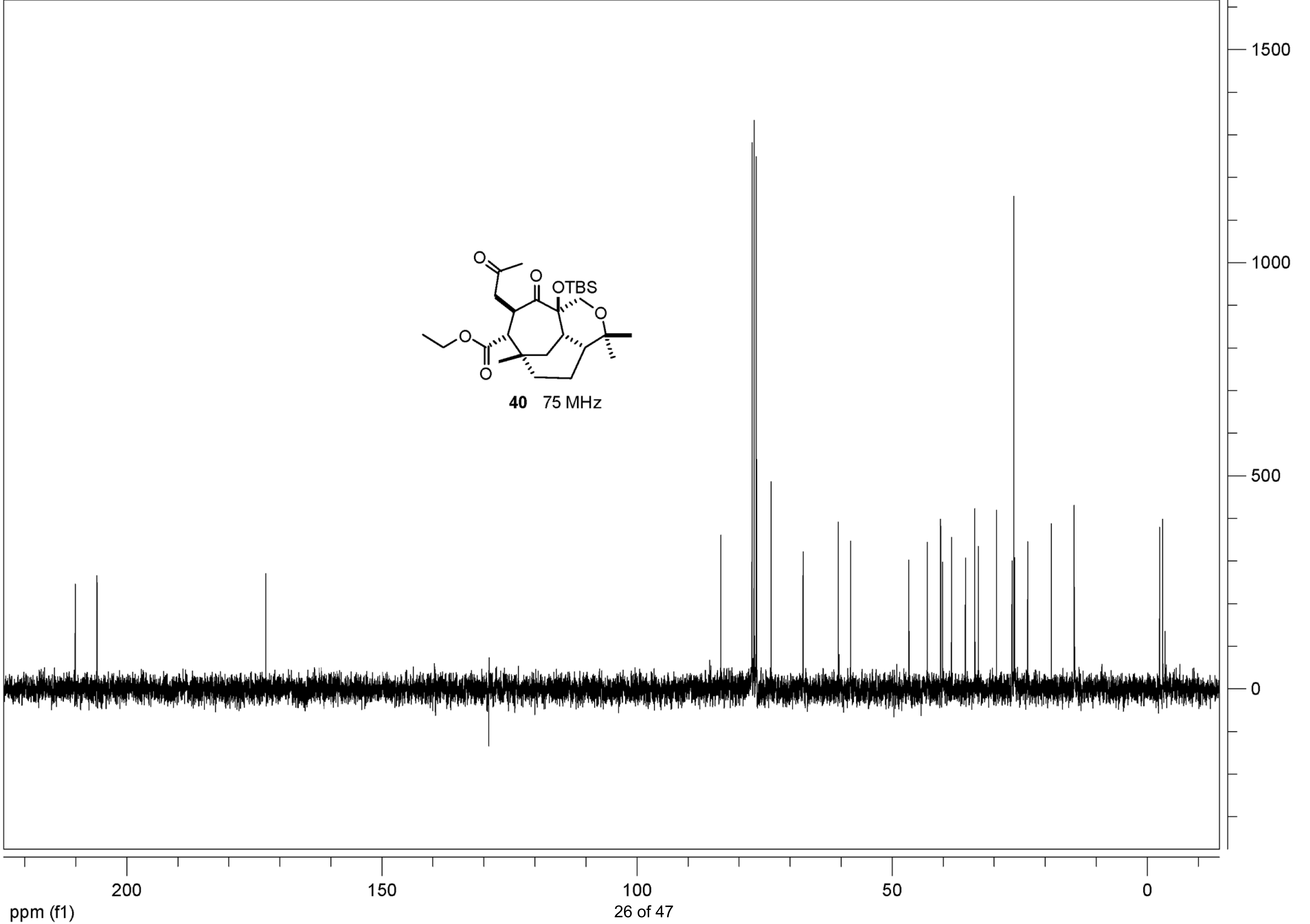

OTBS
40 75 MHz
1500
1000
500
0
200
150
100
50
0
ppm (f1)

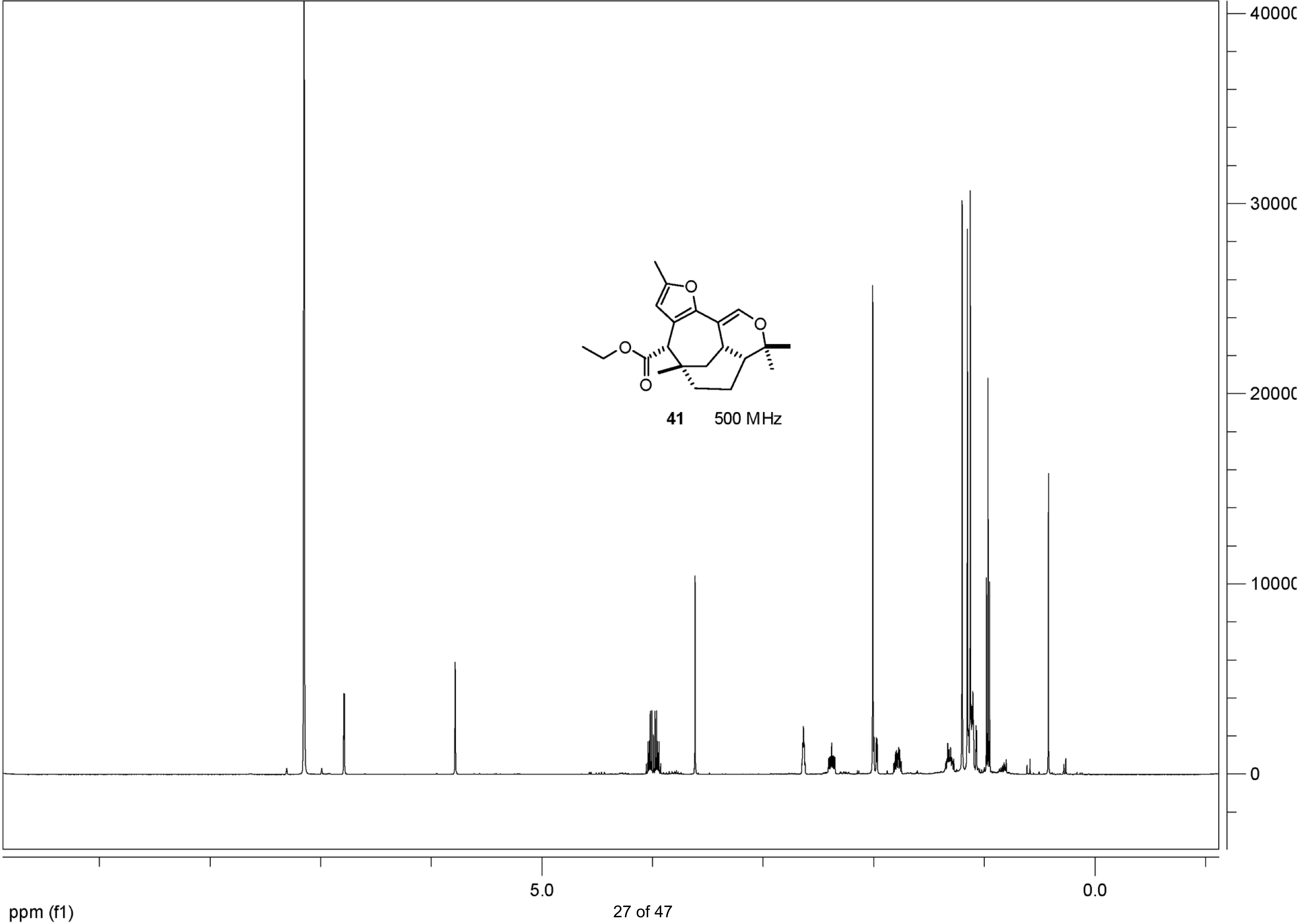
41
500 MHz
40000
30000
20000
10000
0
5.0
0.0
ppm (f1)

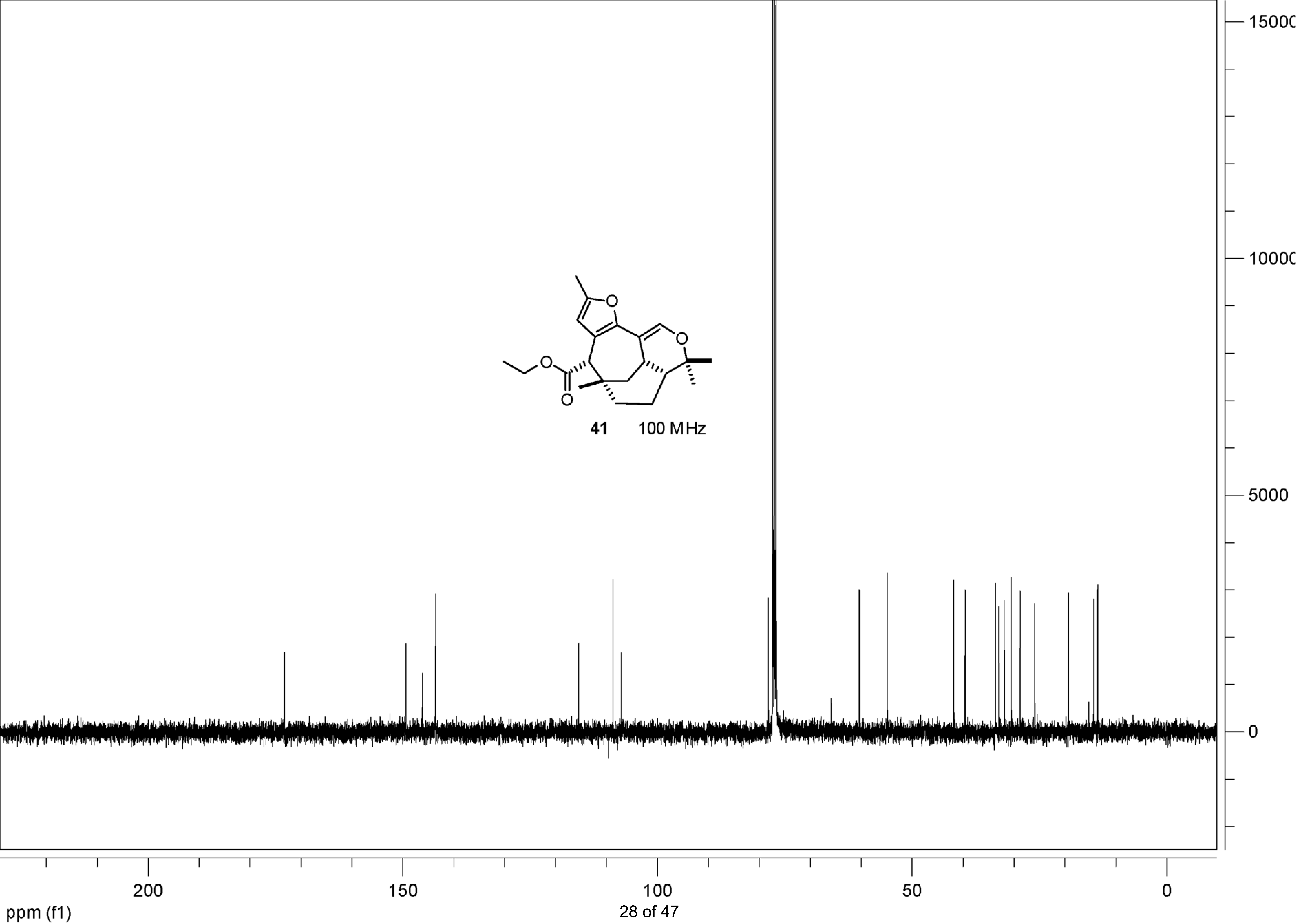

41
100 MHz
15000
10000
5000
0
200
150
100
50
0
ppm (f1)

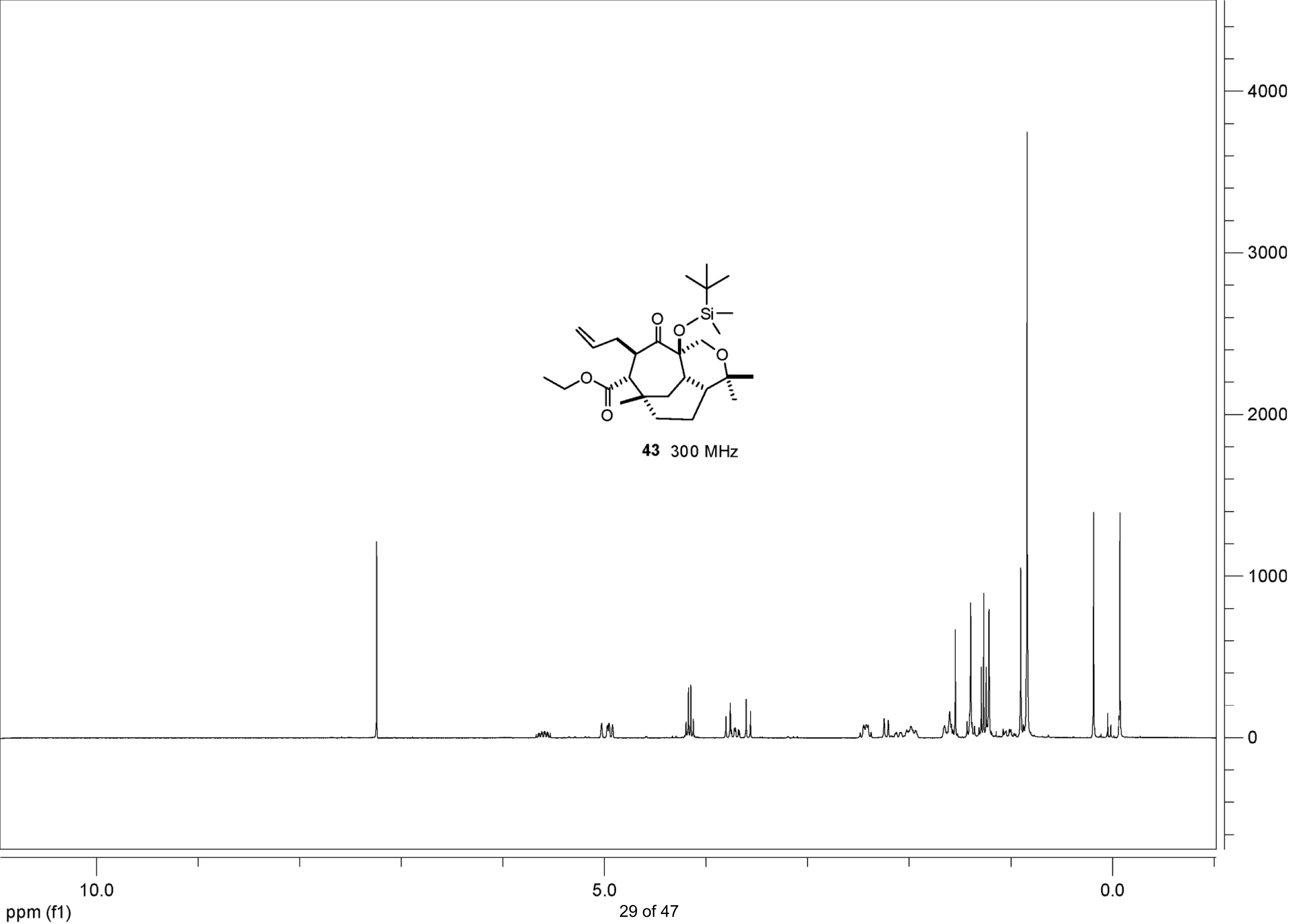
O
O
Si
O
O
O
43 300 MHz
4000
3000
2000
1000
0
10.0
5.0
0.0
ppm (f1)

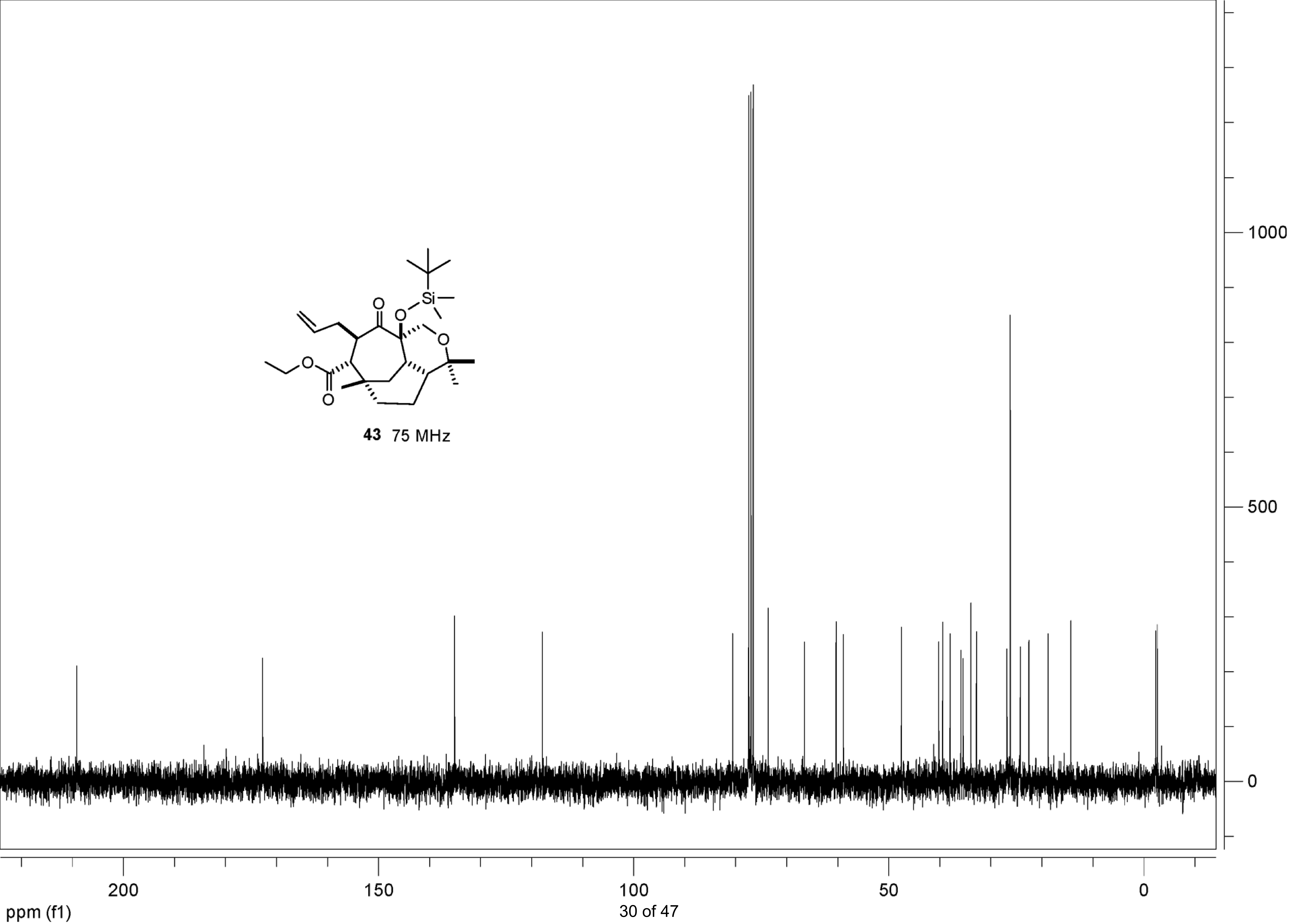

O
O
O
O
Si
O
43 75 MHz
1000
500
0
200
150
100
50
0
ppm (f1)

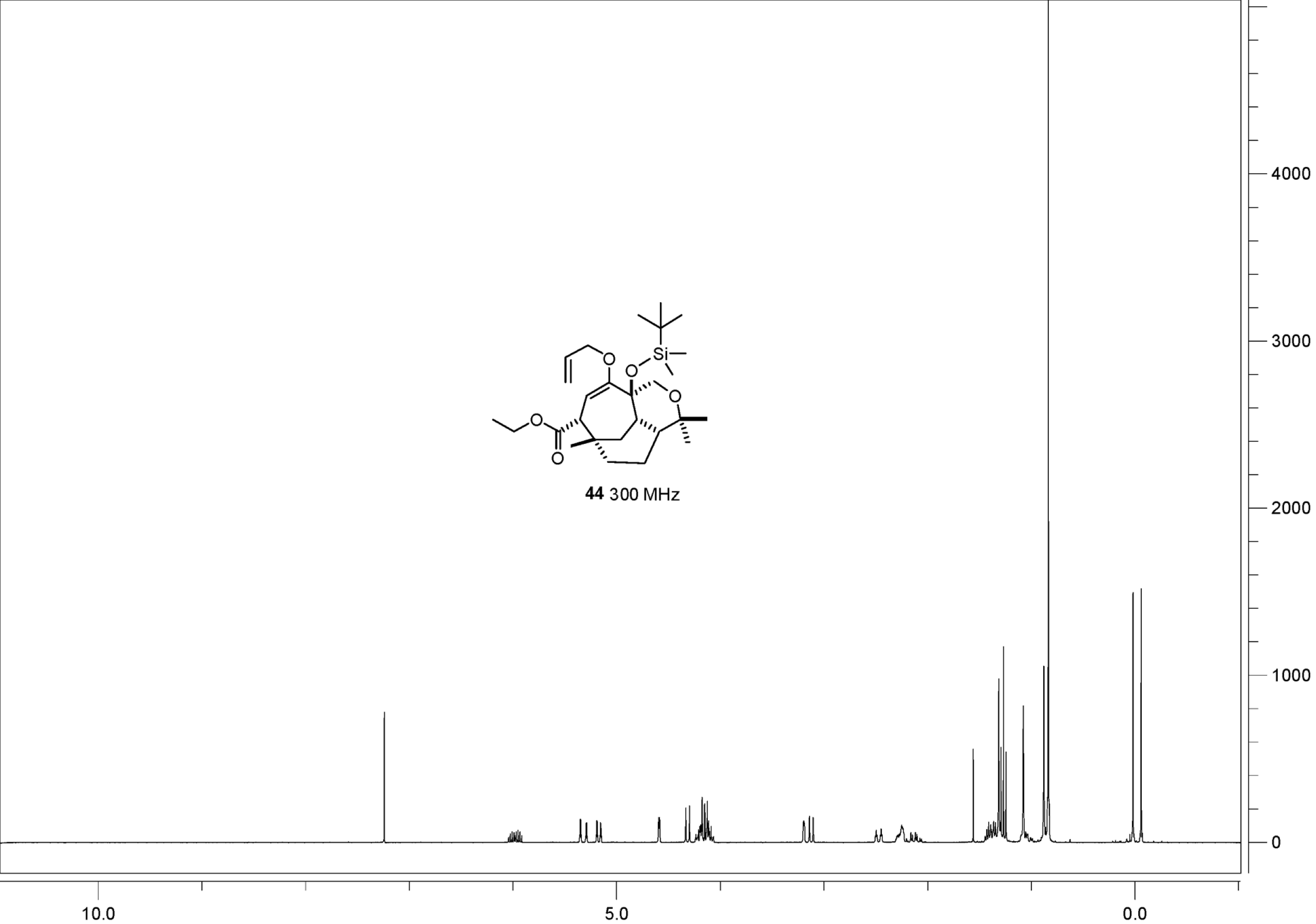

Si
O
O
O
O
O
44 300 MHz
4000
3000
2000
1000
0
10.0
5.0
0.0
ppm (f1)

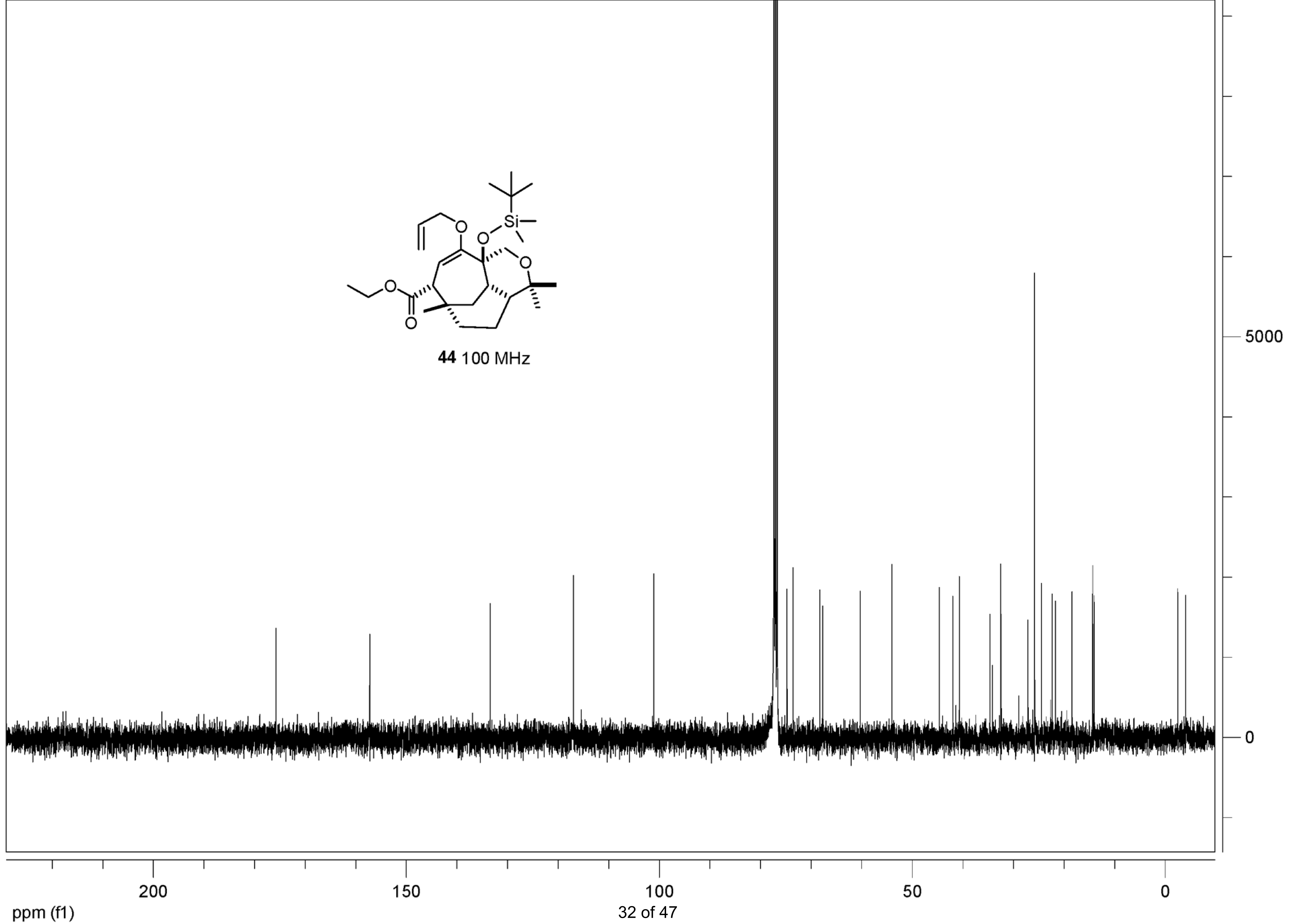
44 100 MHz
5000
0
200
150
100
50
0
ppm (f1)

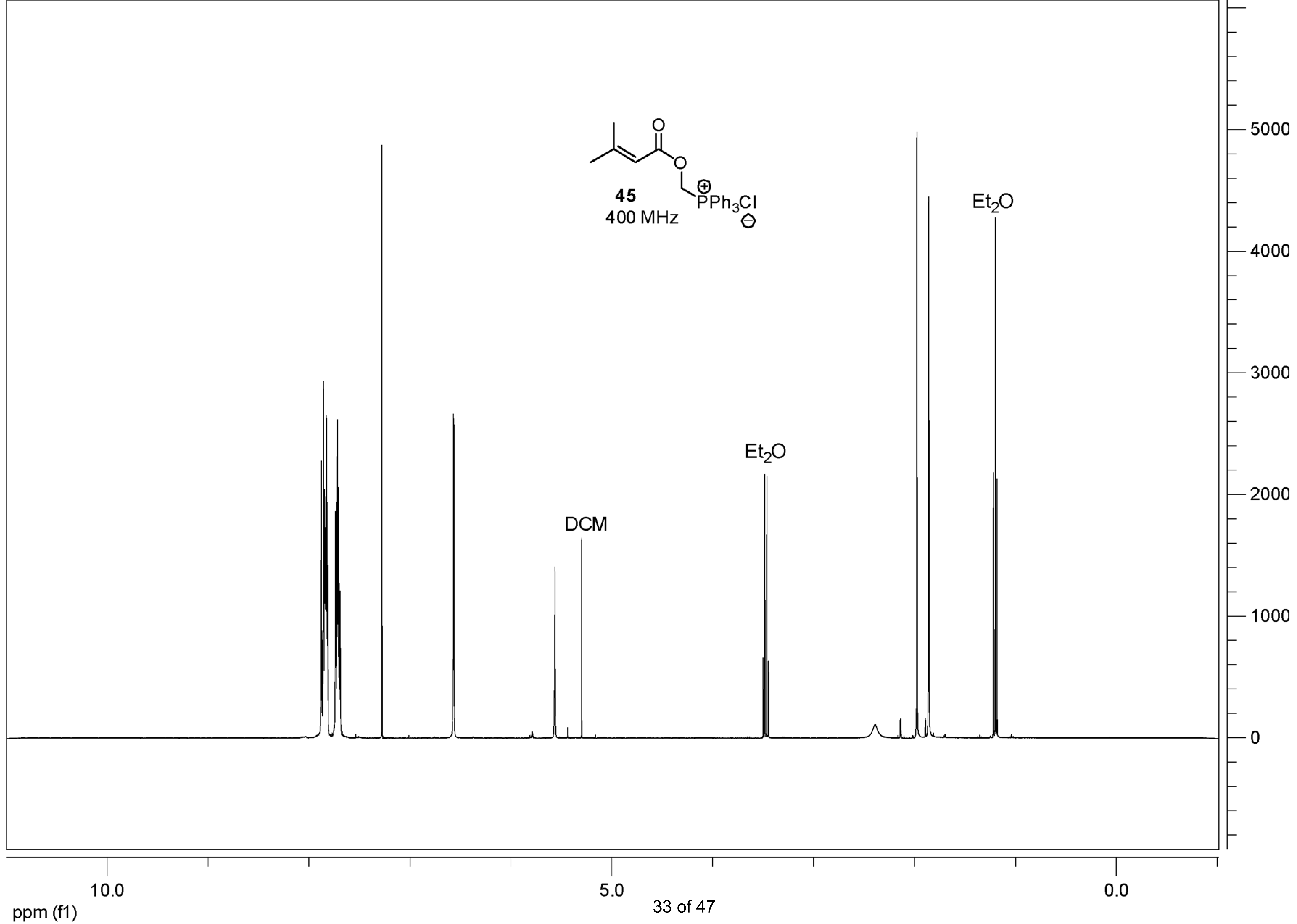
45
400 MHz
PPh3Cl
DCM
Et2O
Et2O
5000
4000
3000
2000
1000
0
10.0
5.0
0.0
ppm (f1)

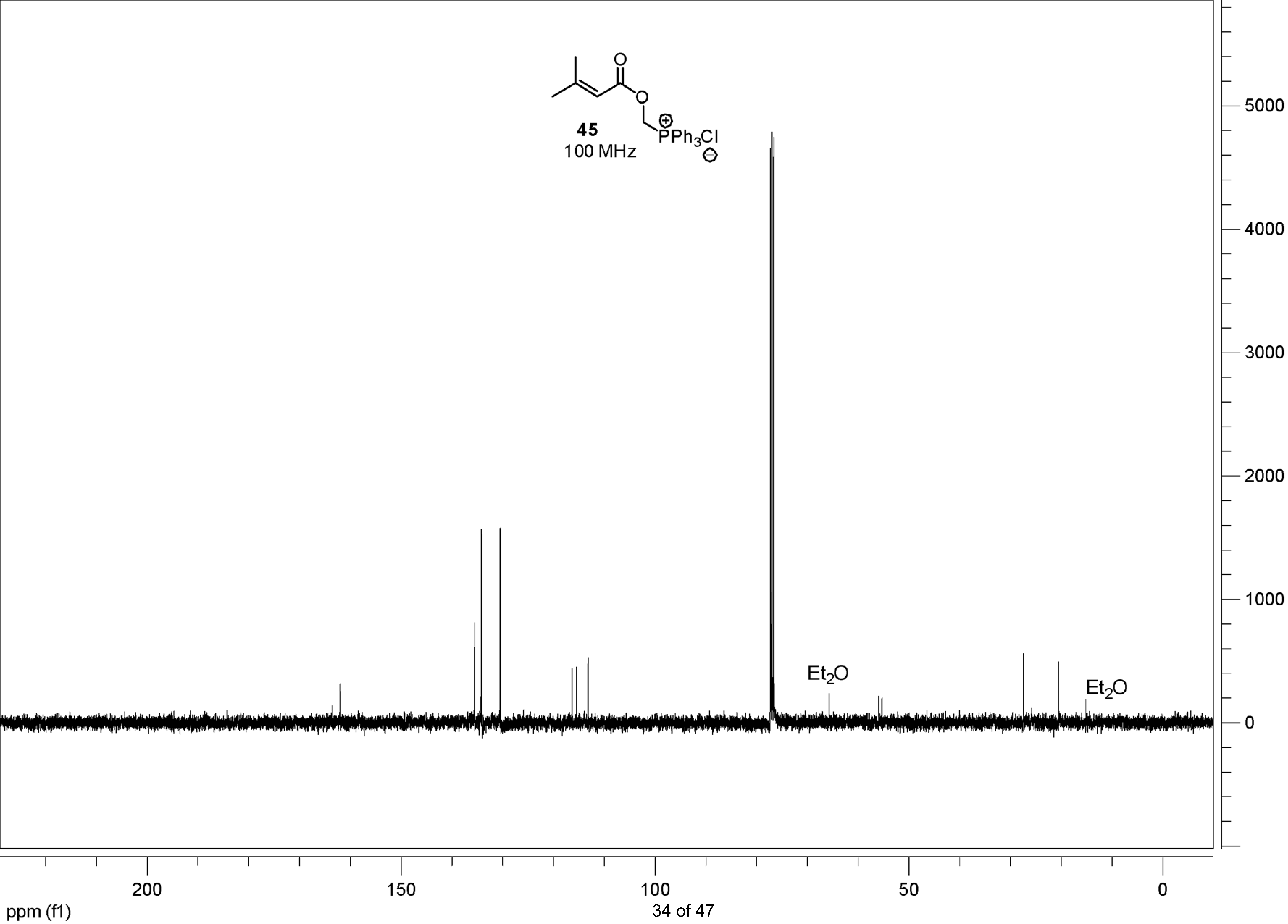
45
100 MHz
PPh3Cl
Et2O
Et2O
5000
4000
3000
2000
1000
0
200
150
100
50
0
ppm (f1)

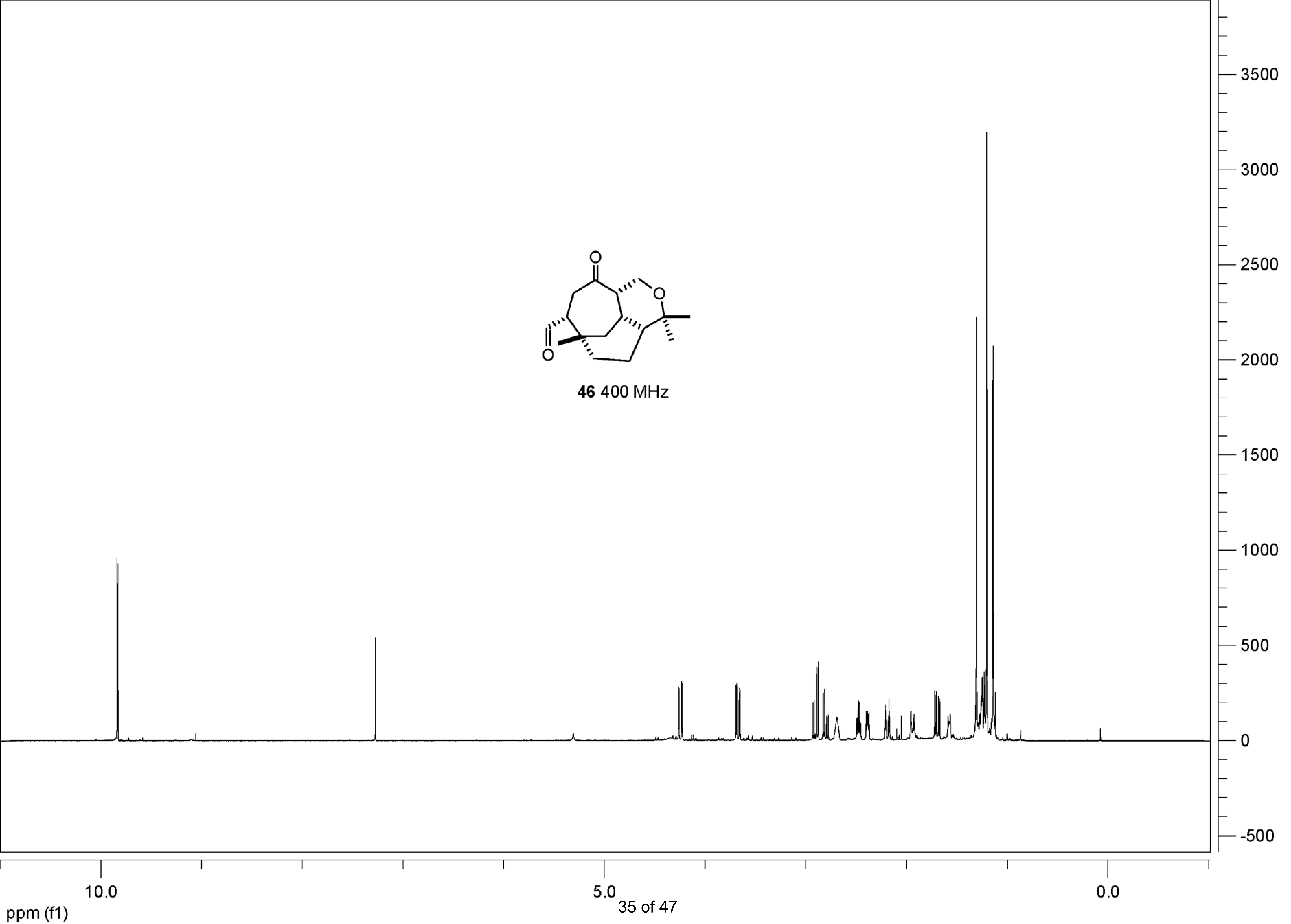

O
O
O
46 400 MHz
3500
3000
2500
2000
1500
1000
500
0
-500
10.0
5.0
0.0
ppm (f1)

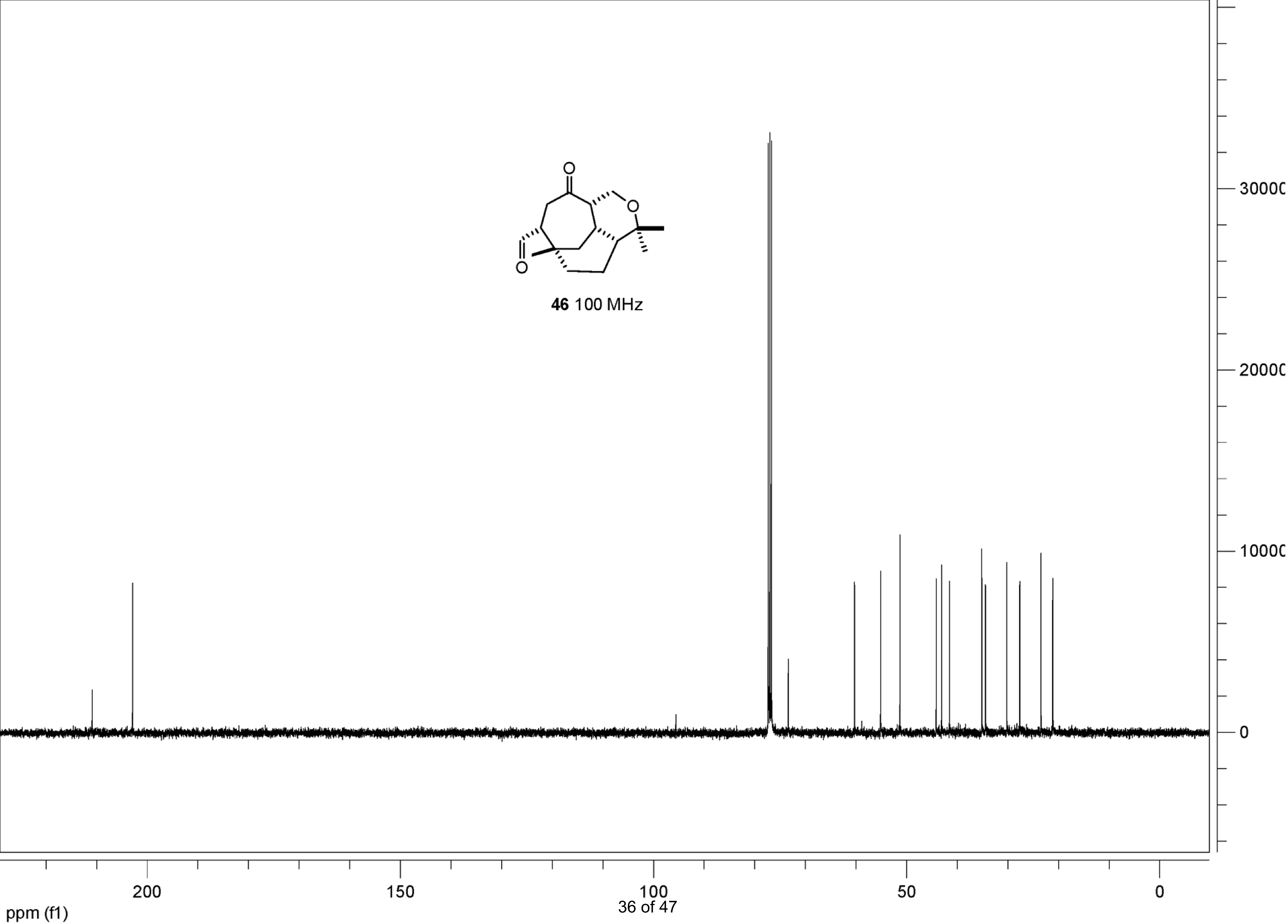
46 100 MHz
30000
20000
10000
0
200
150
100
50
0
ppm (f1)

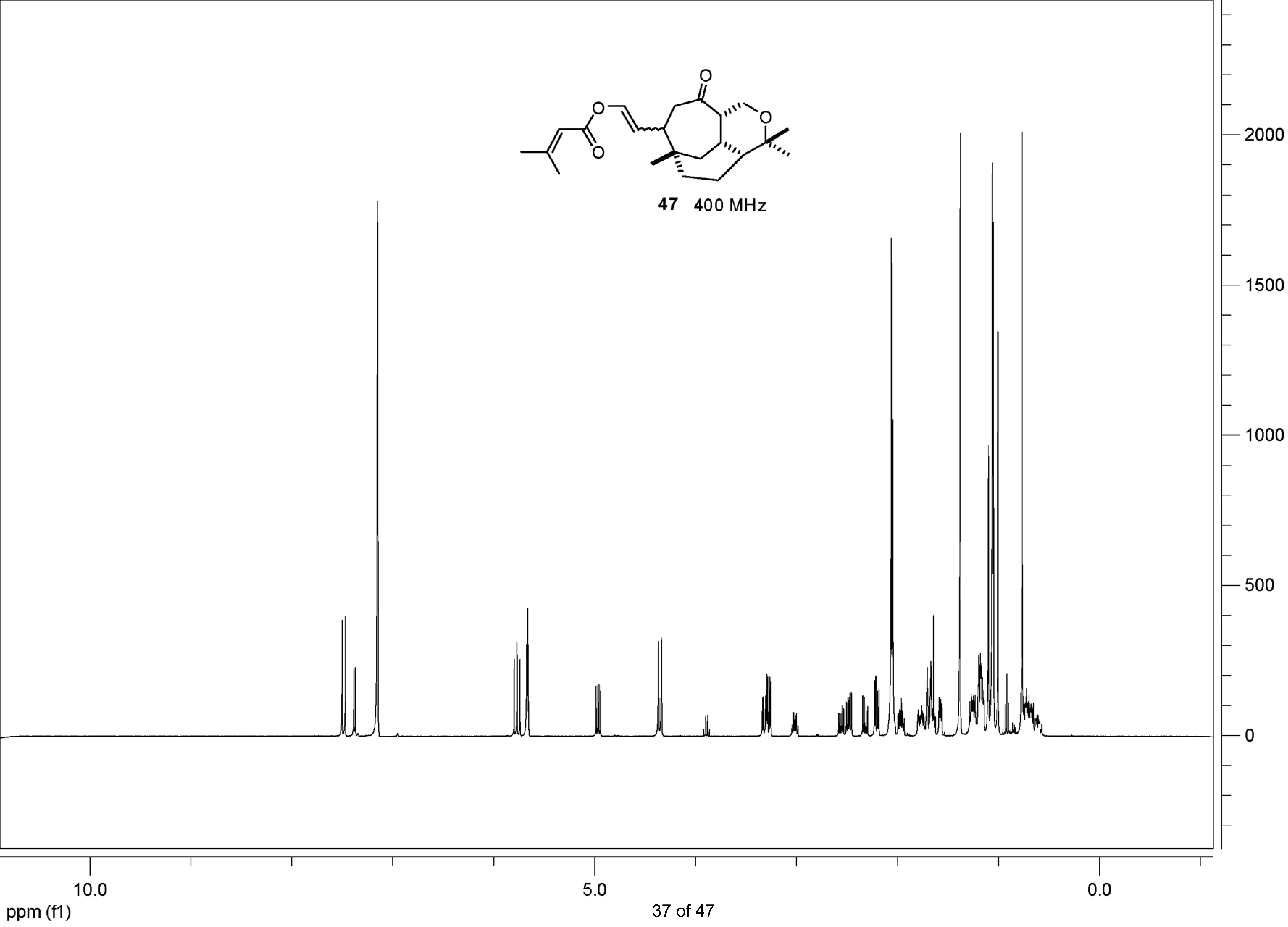
47 400 MHz
2000
1500
1000
500
0
10.0
5.0
0.0
ppm (f1)

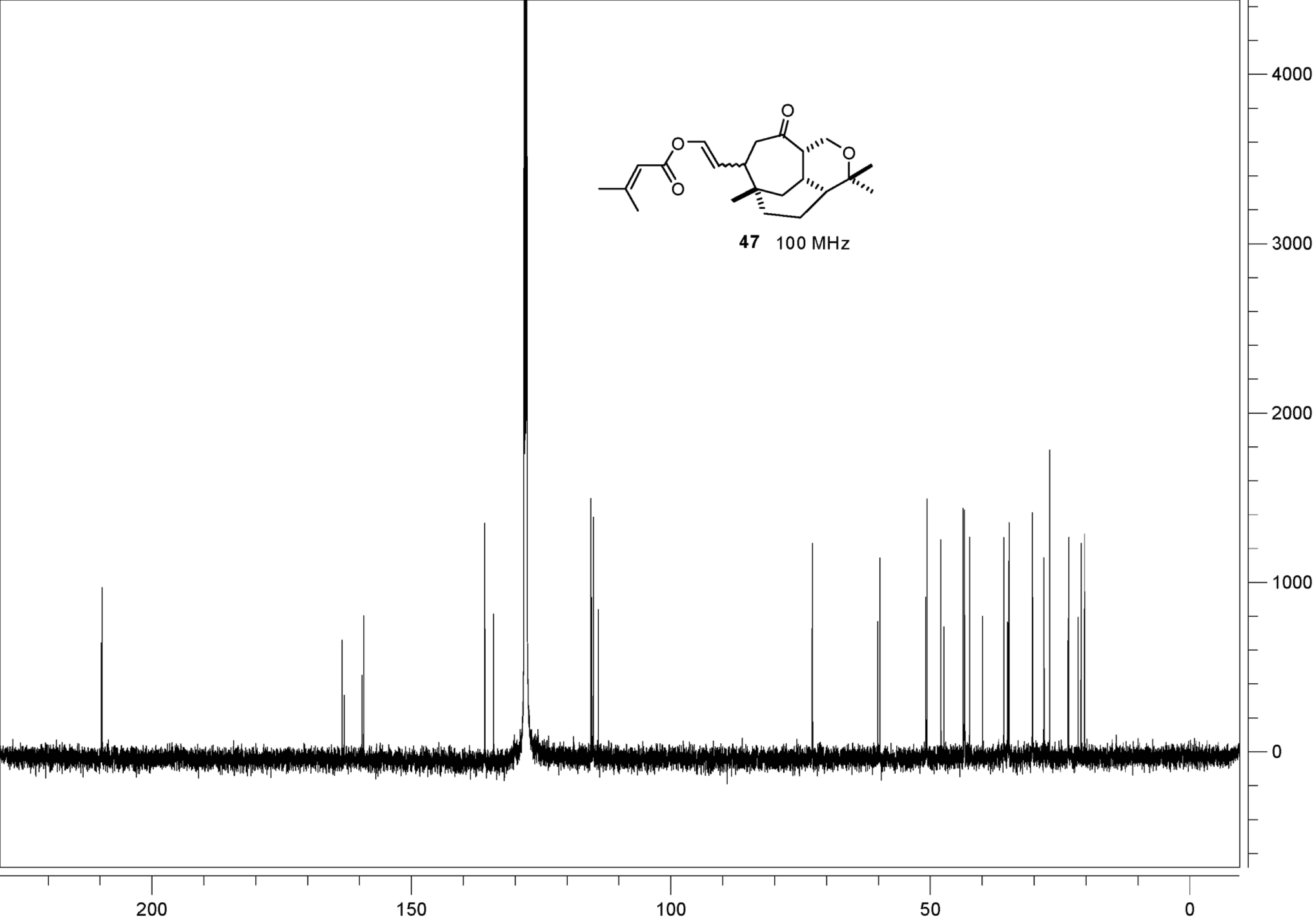
47 100 MHz
4000
3000
2000
1000
0
200
150
100
50
0
ppm (f1)

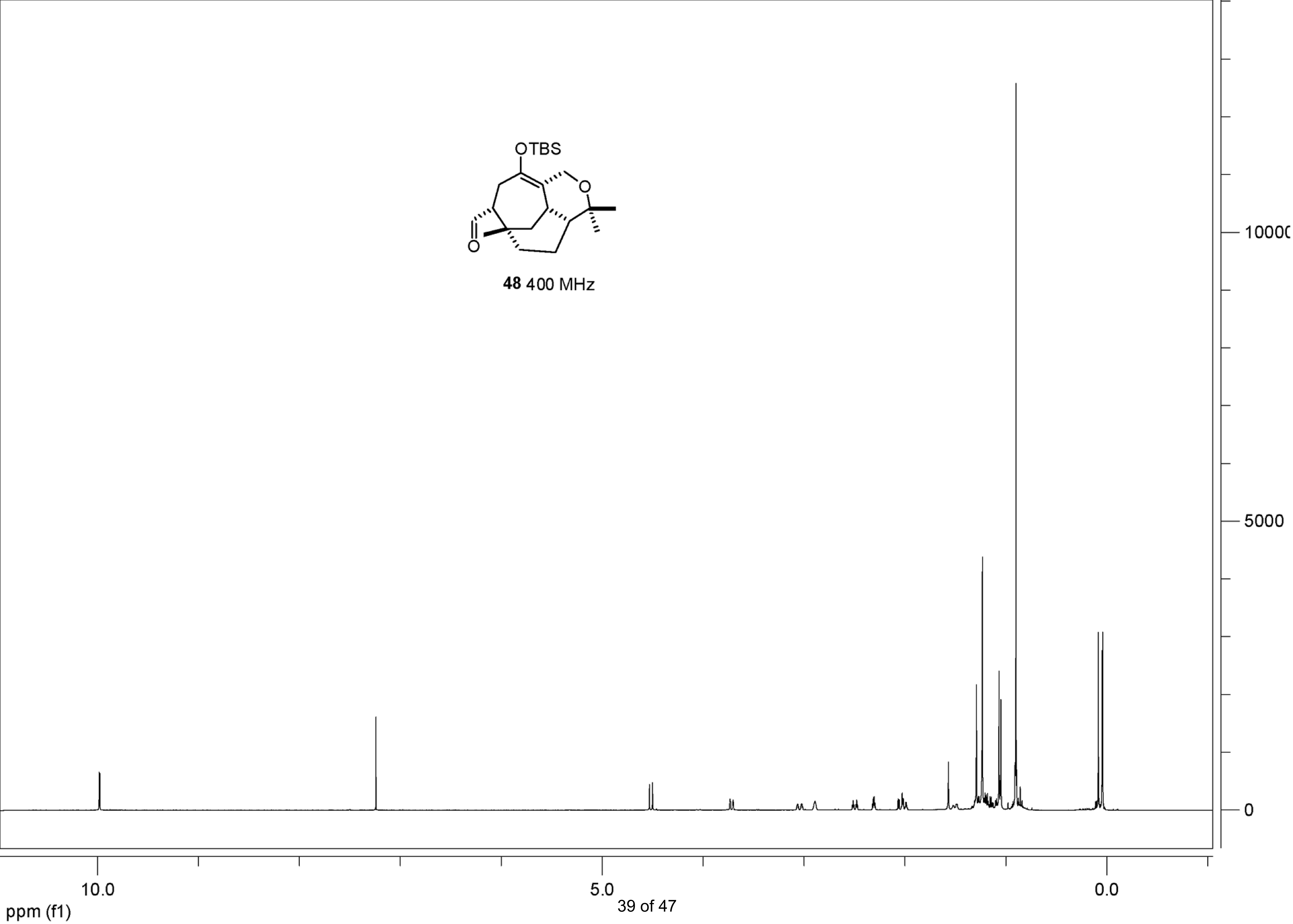
OTBS
O
O
48 400 MHz
10000
5000
0
10.0
5.0
0.0
ppm (f1)

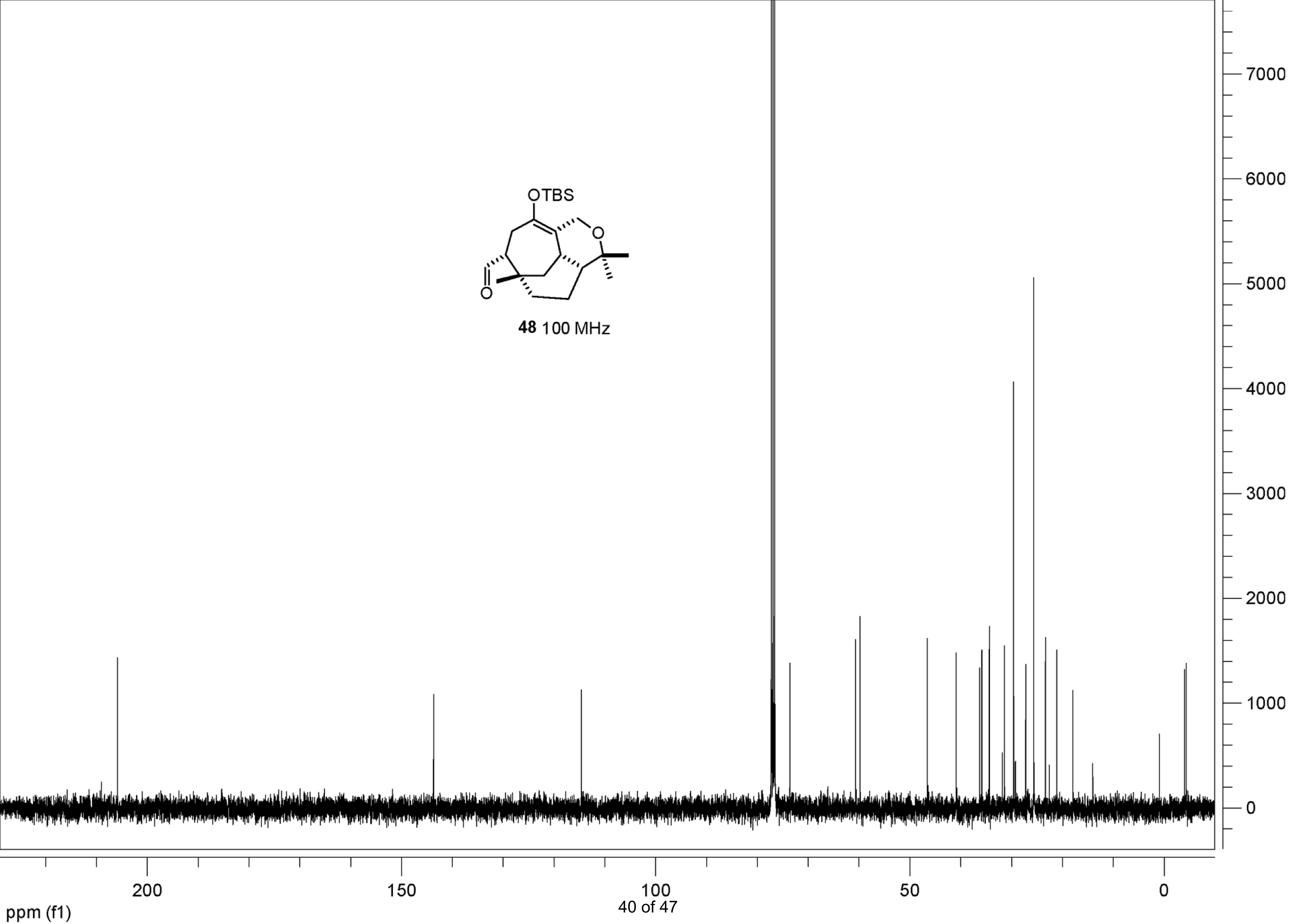
OTBS
O
O
48 100 MHz
7000
6000
5000
4000
3000
2000
1000
0
200
150
100
50
0
ppm (f1)

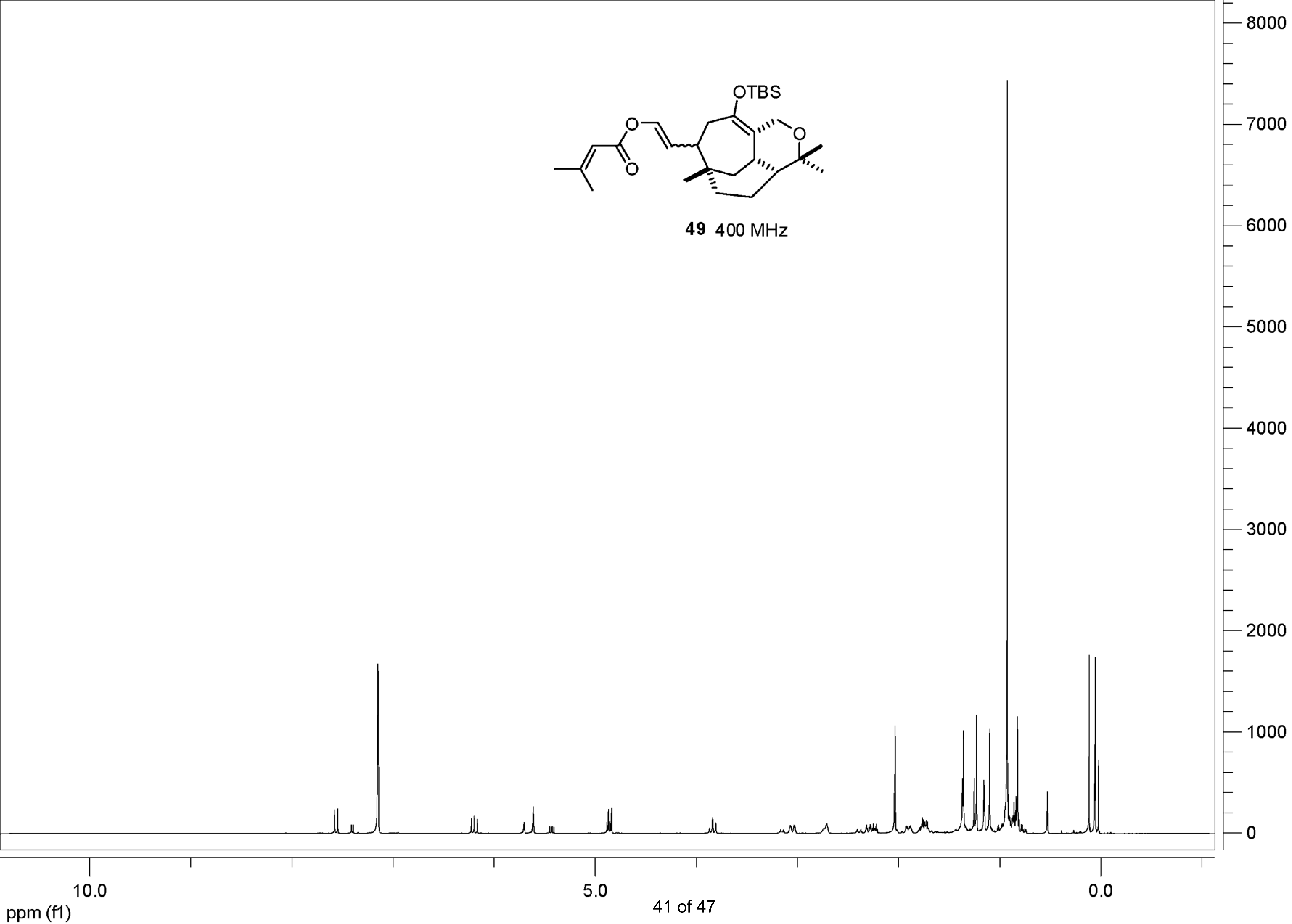
OTBS
O
O
O
49 400 MHz
8000
7000
6000
5000
4000
3000
2000
1000
0
10.0
5.0
0.0
ppm (f1)

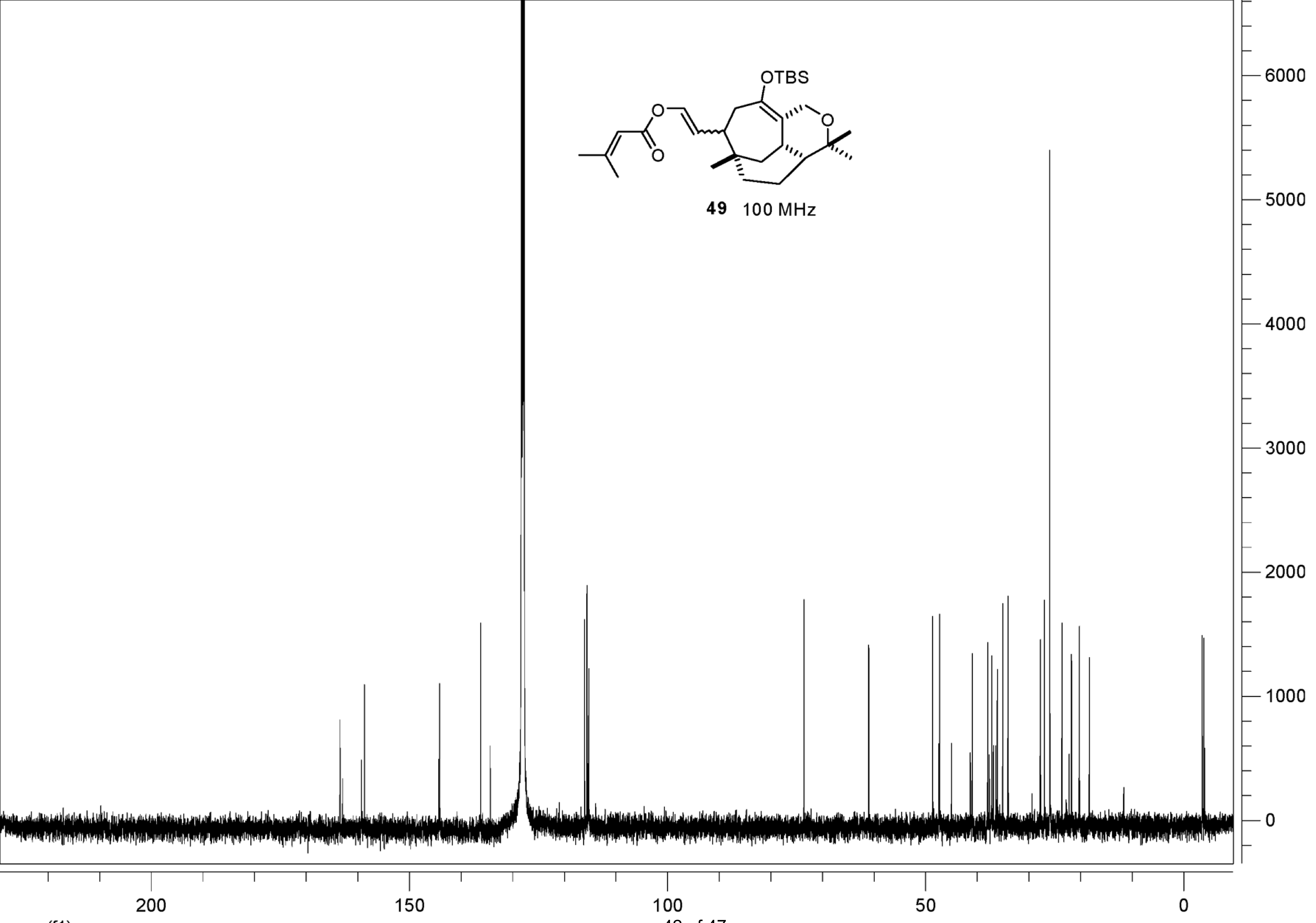
OTBS
49 100 MHz
6000
5000
4000
3000
2000
1000
0
200
150
100
50
0
ppm (f1)

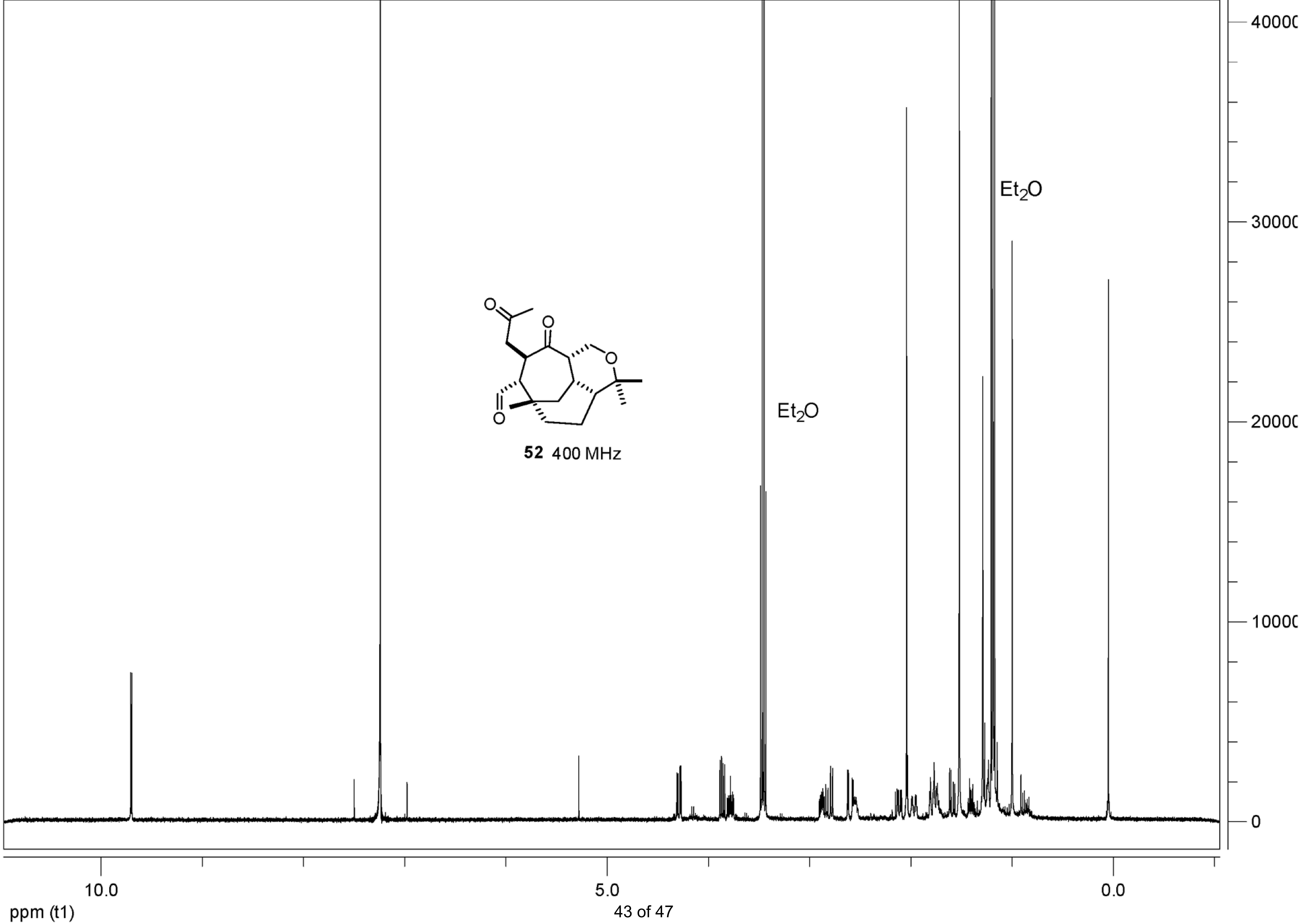
Et$_2$O
Et$_2$O
52 400 MHz
40000
30000
20000
10000
0
10.0
5.0
0.0
ppm (t1)

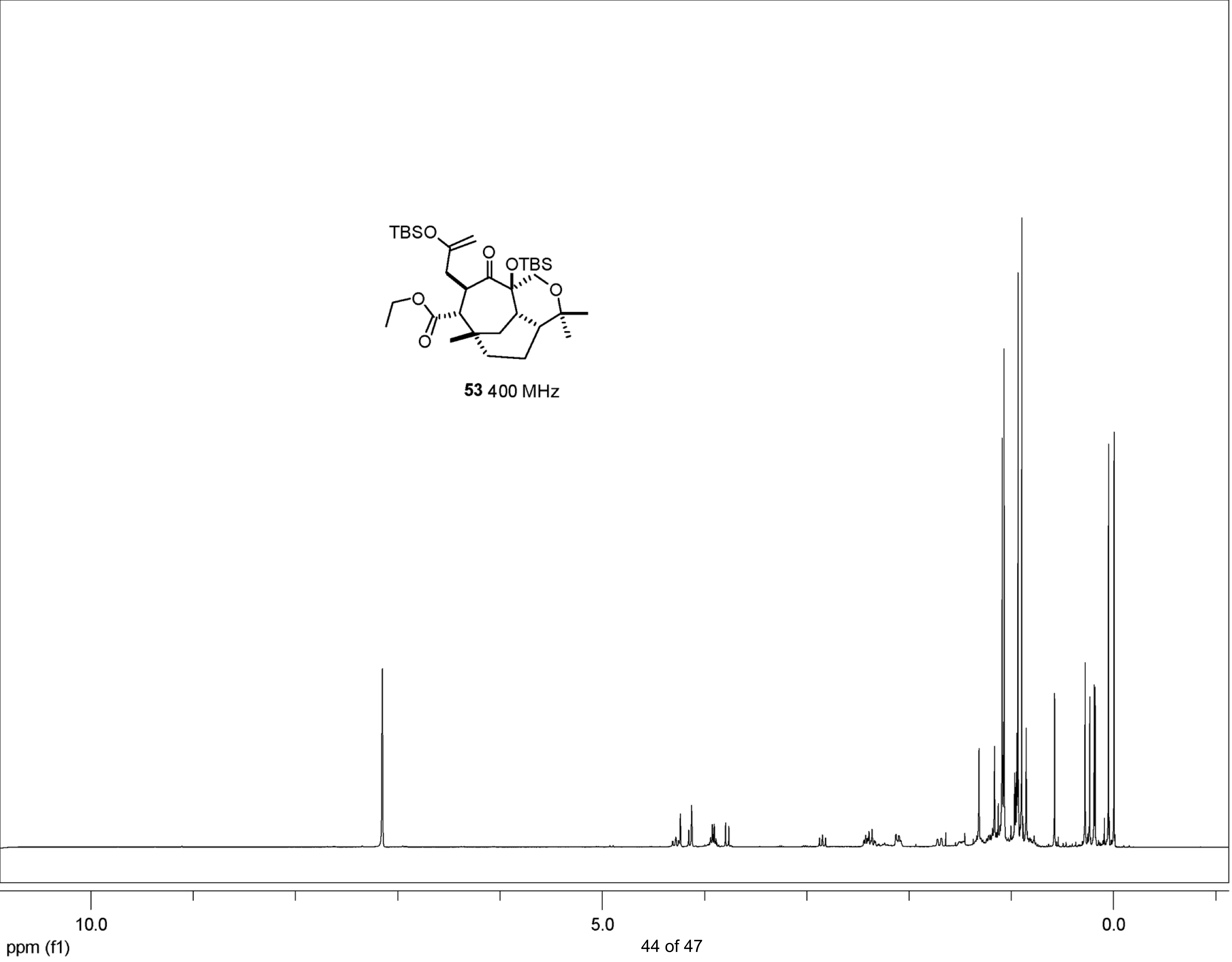
TBSO
OTBS
O
O
O
O
53 400 MHz
10.0
5.0
0.0
ppm (f1)

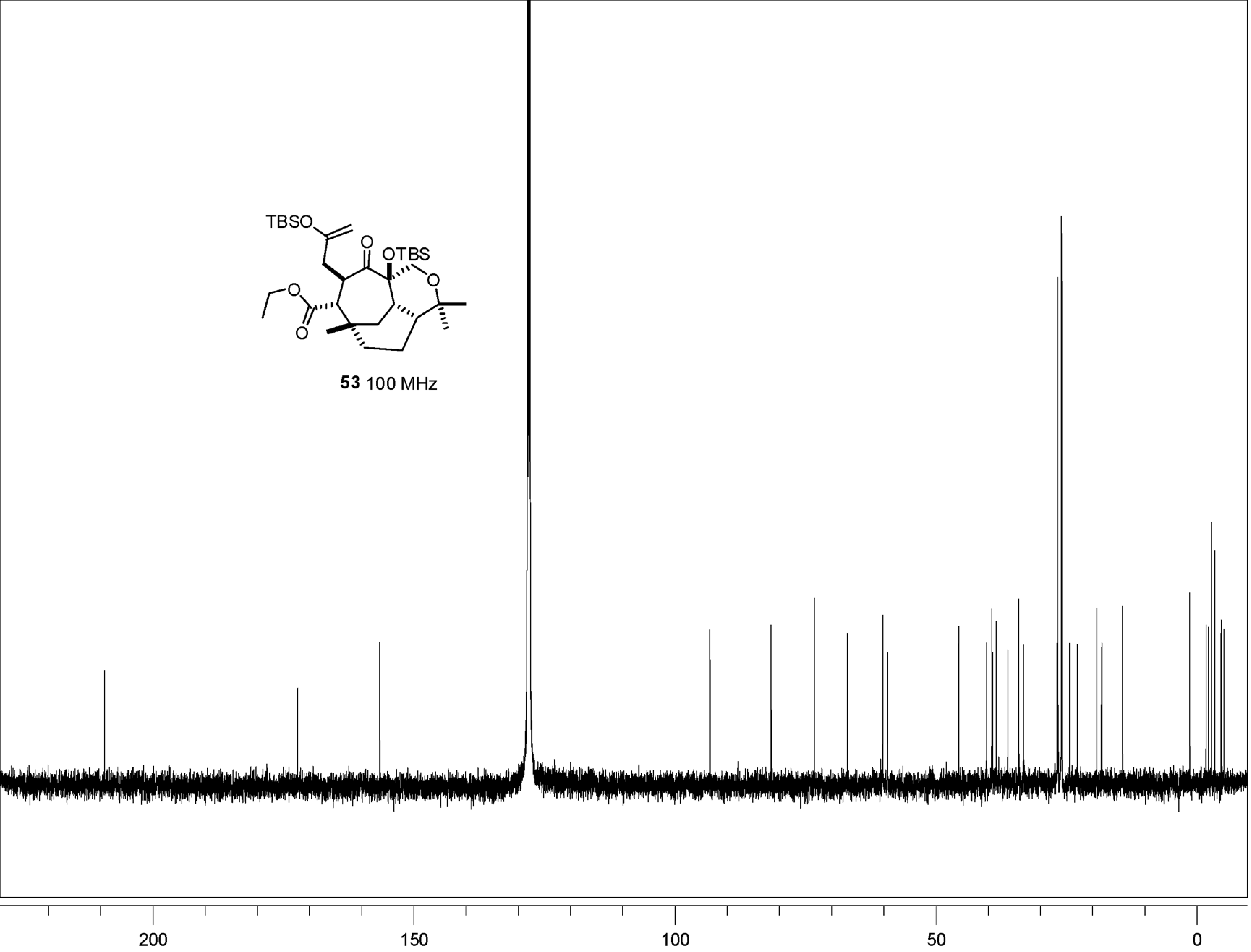

ppm (f1)

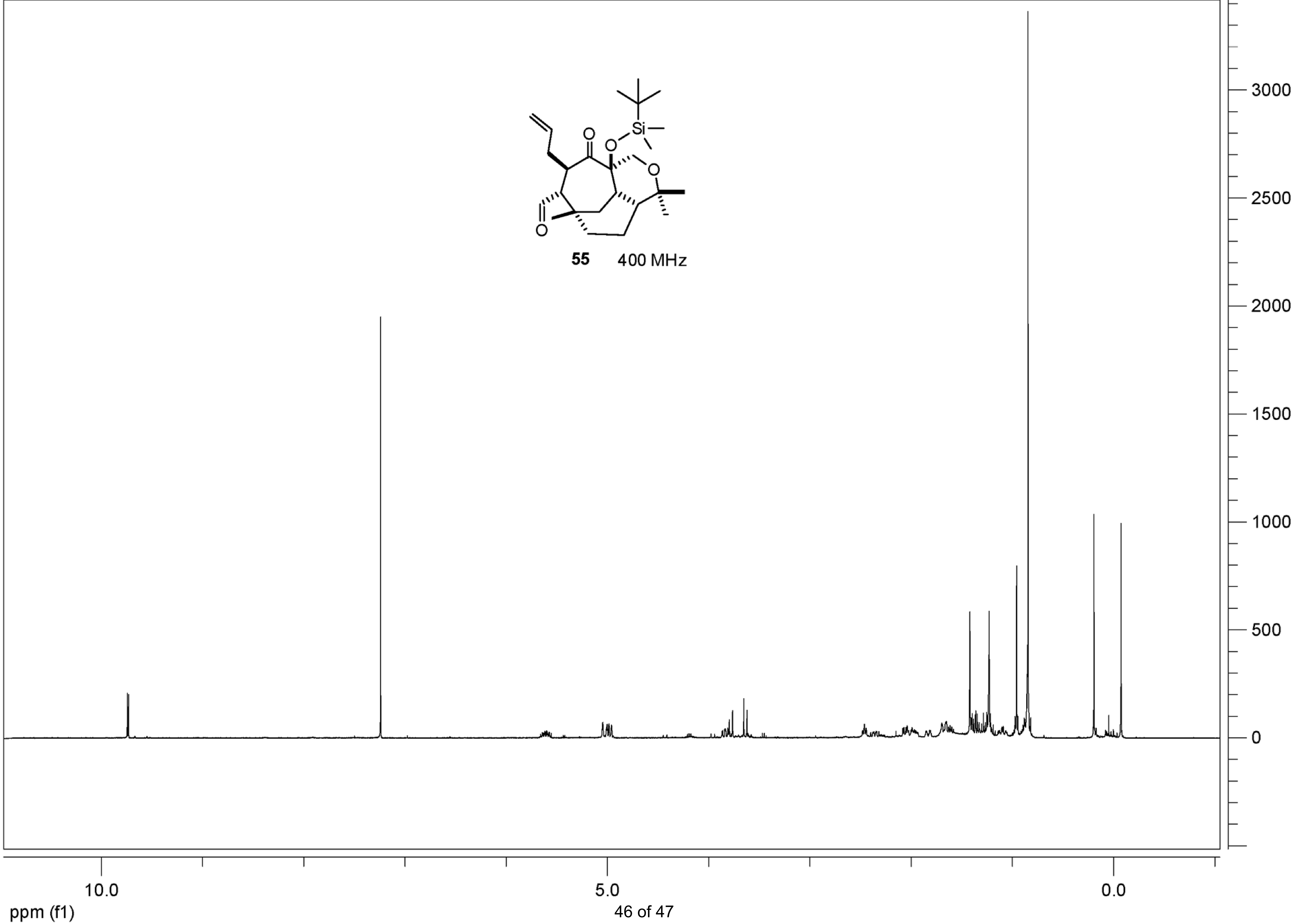
55 400 MHz
3000
2500
2000
1500
1000
500
0
10.0
5.0
0.0
ppm (f1)

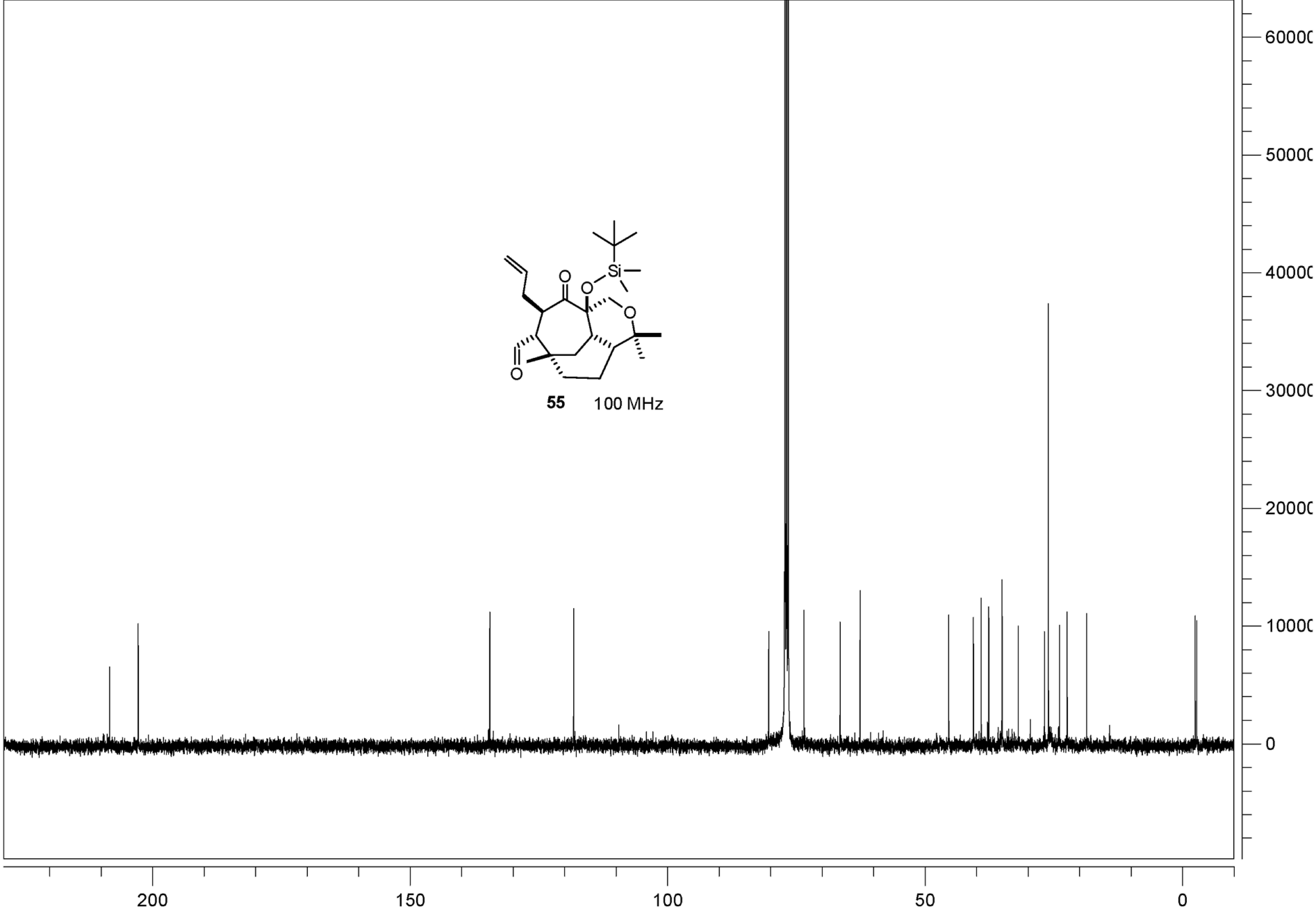
55
100 MHz
6000[illegible]
5000[illegible]
4000[illegible]
3000[illegible]
2000[illegible]
1000[illegible]
0
200
150
100
50
0
ppm (f1)
